# An efficient five-lncRNA signature for lung adenocarcinoma prognosis, with AL606489.1 showing sexual dimorphism

**DOI:** 10.3389/fgene.2022.1052092

**Published:** 2022-11-30

**Authors:** Jiali Liang, Weifeng Jin, Huaping Xu

**Affiliations:** ^1^ The First School of Clinical Medicine, Zhejiang Chinese Medical University, Hangzhou, China; ^2^ School of Pharmaceutical Sciences, Zhejiang Chinese Medical University, Hangzhou, China

**Keywords:** long noncoding RNAs (lncRNAs), The Cancer Genome Atlas (TCGA), gender dimorphism, prognostic prediction, lung adenocarcinoma (LUAD)

## Abstract

**Background:** Lung adenocarcinoma (LUAD) is a sex-biased and easily metastatic malignant disease. A signature based on 5 long non-coding RNAs (lncRNAs) has been established to promote the overall survival (OS) prediction effect on LUAD.

**Methods:** The RNA expression profiles of LUAD patients were obtained from The Cancer Genome Atlas. OS-associated lncRNAs were identified based on the differential expression analysis between LUAD and normal samples followed by survival analysis, univariate and multivariate Cox proportional hazards regression analyses. OS-associated lncRNA with sex dimorphism was determined based on the analysis of expression between males and females. Functional enrichment analysis of the Gene Ontology (GO) terms and the Kyoto Encyclopedia of Genes and Genomes (KEGG) pathways was performed to explore the possible mechanisms of 5-lncRNA signatures.

**Results:** A 5-lncRNA signature (composed of AC068228.1, SATB2-AS1, LINC01843, AC026355.1, and AL606489.1) was found to be effective in predicting high-risk LUAD patients as well as applicable to female and male subgroups and <65-year and ≥65-year age subgroups. The forecasted effect of the 5-lncRNA signature was more efficient and stable than the TNM stage and other clinical risk factors (such as sex and age). Functional enrichment analysis revealed that the mRNA co-expressed with these five OS-related lncRNAs was associated with RNA regulation within the nucleus. AL606489.1 demonstrated a sexual dimorphism that may be associated with microtubule activity.

**Conclusion:** Our 5-lncRNA signature could efficaciously predict the OS of LUAD patients. AL606489.1 demonstrated gender dimorphism, which provides a new direction for mechanistic studies on sexual dimorphism.

## Introduction

Lung cancer is the leading cause of death in cancer patients across the world (Bade and Cruz, 2020). Approximately 20% of the lung cancer cases are accounted for by small-cell lung cancer, while the remaining 80% are accounted for by non-small-cell lung cancer (Leung et al., 2016). Lung adenocarcinoma (LUAD) and lung squamous cell carcinoma are the most common subtypes of non-small-cell lung cancer (Herbst et al., 2018). LUAD shifts earlier than lung squamous cell carcinoma (Chen et al., 2017); therefore, it is very important to have effective early diagnostic methods for LUAD. Epidemiological studies have revealed that LUAD varies between males and females, with the highest incidence of occurrence among never-smokers and women (Couraud et al., 2012). Meanwhile, several other factors affect lung cancer (Paz-Ares et al., 2018). In addition to the well-known risk factors such as tobacco, a close association of genetic variants has been demonstrated in multiple studies with the risk of lung cancer (Li and Hemminki, 2004; Musolf et al., 2016; Cheng et al., 2019).

With the widespread development of the human genome program (Collins et al., 2003), several genes have been highlighted as being probably related to the onset of lung cancer, including AKT (Hyman et al., 2017), BRD4 (Zhang et al., 2021), FGFR1 (Yuan et al., 2017), BRAF (Lokhandwala et al., 2019), MET (Wu et al., 2020), PIK3CA (Wang et al., 2020), and EGFR (Zhao et al., 2021). However, the current reports on the long non-coding RNA (lncRNA) remain inadequate. LncRNA refers to any polyadenylated RNA of length >200 bp (Quinn and Chang, 2016), which forms a transcript of a large portion of the eukaryotic genome (Jathar et al., 2017). In recent years, lncRNA has received continuous attention from researchers for its important role in the eukaryotic gene expression and genome remodeling (Cech and Steitz, 2014; Fungtammasan et al., 2015), including from the tumor perspective (Hauptman and Glavač, 2013). Up to 37,595 non-coding genes (Snyder et al., 2020) have been identified, according to the latest data from the ENCODE Project Consortium 2018 (Davis et al., 2018). Clearly, the number of current studies on lncRNA are insufficient compared to this large number of genes.

In numerous lncRNA-related studies, lncRNA has been widely reported as a biomarker in the diseases of multiple systems (Zhang et al., 2018; Yu et al., 2019). Ideal biomarkers not only facilitate the early diagnosis of disease (Xu et al., 2020) but also predict patient prognosis (Chao and Zhou, 2019) as well as become potential drug therapeutic targets (Tamang et al., 2019). In the field of LUAD, the effect of lncRNA as a biomarker on tumor cells has been explored in terms of immunity (Li et al., 2020), ferroptosis (Lu et al., 2021), and cell pyroptosis (Li et al., 2018). However, for LUAD, as a typical sex-biased (Yuan et al., 2016) malignancy, no investigation has yet explored the possible mechanisms of sex-biased differences in LUAD through the biomarker role of lncRNA. The occurrence of cancer is affected by gender differences, which is a consistent finding in the field of cancer epidemiology (Dorak and Karpuzoglu, 2012). Liu et al. analyzed the sex differences in lncRNAs across different cancers and found that LINC 00263 acts as an oncogene associated with men and estrogens; these findings may help explore the differential gene regulatory mechanisms in sex-specific cancers (Liu et al., 2020).

In conclusion, this study identified the prognostic models of LUAD through information mining from public databases and explored the possible mechanisms of sex differences in LUAD. Alternatively, as The Cancer Genome Atlas (TCGA) contains the most extensive lncRNA expression matrix (Tomczak et al., 2015), we prefer to conduct experiments in the TCGA database.

## Materials and methods

### Data sources

The lncRNA and mRNA expression dataset in the FPKM format as well as the clinical characters for 535 LUAD patients and 59 normal patients were directly downloaded from the TCGA (https://portal.gdc.cancer.gov/), updated until 5 December 2021. GEO database (https://www.ncbi.nlm.nih.gov/geo/) was used to perform external validation.

### Isolation of differentially expressed lncRNA

DELs between the LUAD and normal samples were isolated from all lncRNAs using the R software. The *p*-value of each lncRNA in the LUAD and normal samples was calculated by the rank-sum test, and the *p*-values were rectified by the False Discovery Rate (FDR) method. Only the lncRNAs with adjusted *p*-values < 0.05 and log2 | fold change | values > 2 were defined as differentially expressed lncRNAs. Volcano plot and heatmaps were visualized by the “plot” function and the “heatmap” package of the R, respectively.

### Isolation of overall survival-related lncRNAs in LUAD patients

First, we removed LUAD patients with OS < 0 days. Next, we employed univariate Cox proportional hazards regression (CPHR) analysis and Kaplan-Meier analysis to assess the presence of any significant correlations between the expression of each DELs and the OS of LUAD patients. Only lncRNAs with *p* < 0.01 from both the analyses were considered with a logical agreement in expression and prognostic effect and selected as the candidate OS-related lncRNAs. Then, half of the patients were randomly assigned as the “primary dataset” after removing patients with incomplete clinical information; the original complete dataset was called the “entire dataset”. In addition to randomization, the criteria for grouping included no statistical differences in the clinical characteristics between the “primary” and “entire datasets. In order to fit the prediction model with the best-prediction effect, multivariate CPHR analysis (stepwise model) of candidate OS-associated lncRNAs was performed with the R software in the “primary dataset”. To ensure the goodness of fitting and to avoid overfitting, the Akaike information criterion (AIC) was computed, and the prediction model with the lowest AIC was considered as the most ideal. LncRNAs included in the best prediction model were selected as OS-related lncRNAs.

### Calculation and evaluation of the OS-related lncRNA signature

We determined the coefficients for each lncRNA by another multivariate CPHR analysis in the “primary dataset”. Until this point, we confirmed a risk score formula with the expressions of the OS-related lncRNAs as the independent variables and weighted by the regression coefficients corresponding to the lncRNAs. The risk scoring formula used is given below:

$$Risk\;Score=\left(\beta_{1}\times Expression\;{gene}_{1}\right)+\left(\beta_{2}\times Expression\;{gene}_{2}\right)+\cdots +\left(\beta_{n}\times Expression\;{gene}_{n}\right)$$

where βi correspond to the correlation coefficient.

To determine whether the OS-related lncRNA signature was an independent predictor of OS, we applied both univariate and multivariate CPHR analyses of OS-related lncRNA signature and the routine clinical risk factors (such as sex, age, TNM stage, tumor stage, lymph node metastasis, and distant metastasis) in the LUAD patients. Next, we assessed whether the predictive effect of the OS-related lncRNA signature on OS was independent of the routine clinical risk factors by stratified analysis. Meanwhile, to evaluate the prognostic effect of the lncRNA-based classifiers across different time ranges, we plotted the time-dependent receiver operating characteristic (ROC) curves and then calculated the area under the time-dependent ROC curve (AUC) values for each dataset. Finally, the predictive effects of the 5-lncRNA classifier and the classifiers based on the other clinical risk factors were compared by AUC.

### Identification of OS-related lncRNAs with gender dimorphism

Whether the OS-related lncRNA was differentially expressed between the male and female patients was determined by the rank-sum test using *p* < 0.05 as the significance threshold. In both the male and female groups, the patients were assigned into two groups of high or low expression bounded by the median expression of an OS-related lncRNA, and the Kaplan–Meier curve was applied to analyze whether there were differences in survival time between the high and low expression groups. The lncRNA was considered to be with gender dimorphism if an OS-related lncRNA was differentially expressed in males and females while showing different prognostic association in males and females.

### Functional enrichment analyses with co-expressed mRNA

The co-expression degree of OS-related lncRNAs and mRNA was determined by Pearson’s correlational analysis. The mRNAs with a positive correlation coefficient >0.5 with OS-related lncRNAs were employed in the next step of enrichment analysis. The “cluster profile” package in R software was used for the functional enrichment analysis using the Gene Ontology (GO) terms and Kyoto Encyclopedia of Gene and Genomes (KEGG) pathways (Kanehisa and Goto, 2000; Kanehisa, 2019; Kanehisa et al., 2021), with *p* < 0.01 set as a significance threshold.

### Statistical analysis

For the survival analysis, the survival curves were plotted by the Kaplan-Meier method, and the differential *p*-values were calculated by the log-rank test. The *t*-test was used to compare the presence of any significant differences between the “primary” and “entire datasets”. Unless otherwise specified, *p* < 0.05 was considered to indicate a statistical difference.

## Results

### Candidate OS-related lncRNAs in LUAD patients

**FIGURE 1:**
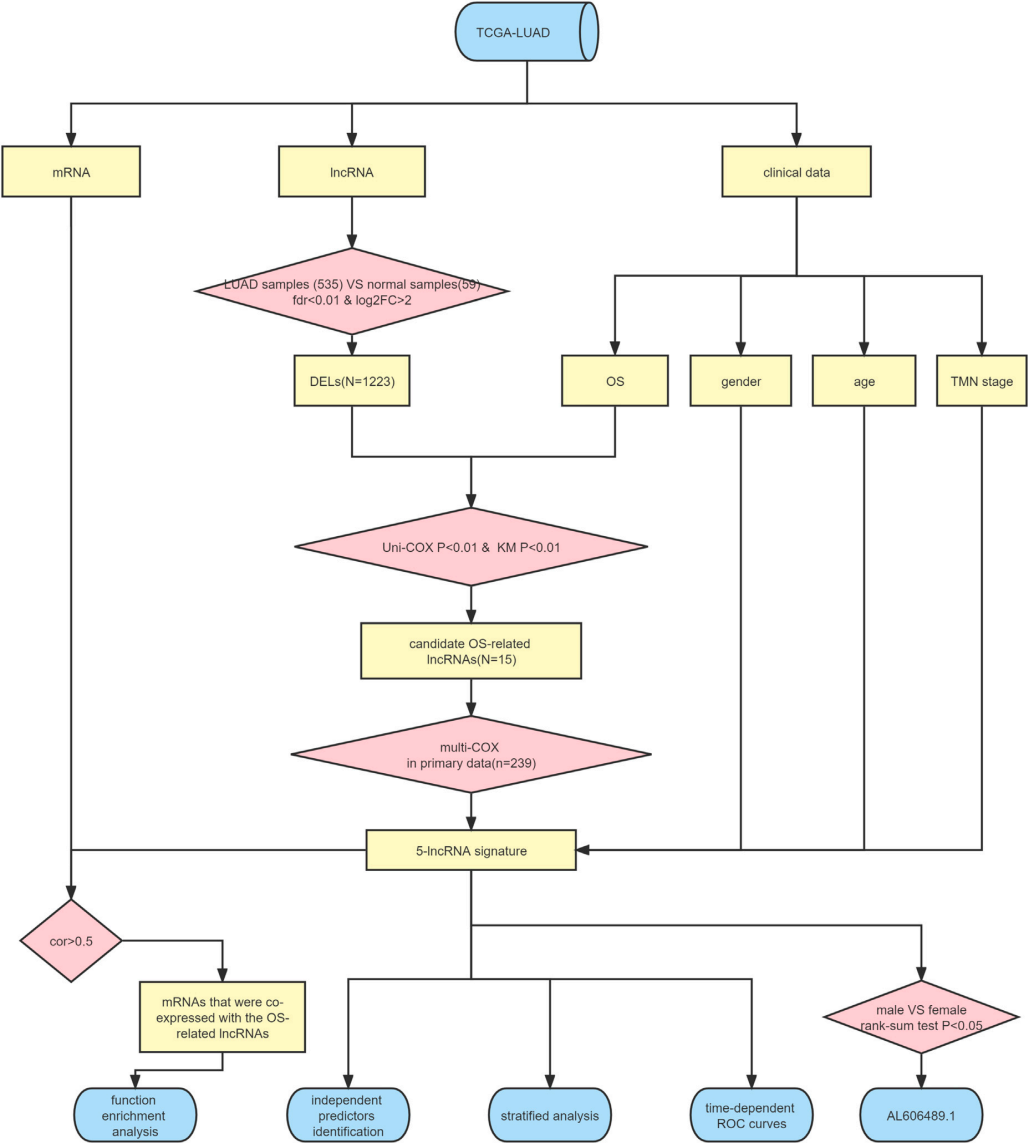
Flowchart depicting the study protocol.

**FIGURE 2:**
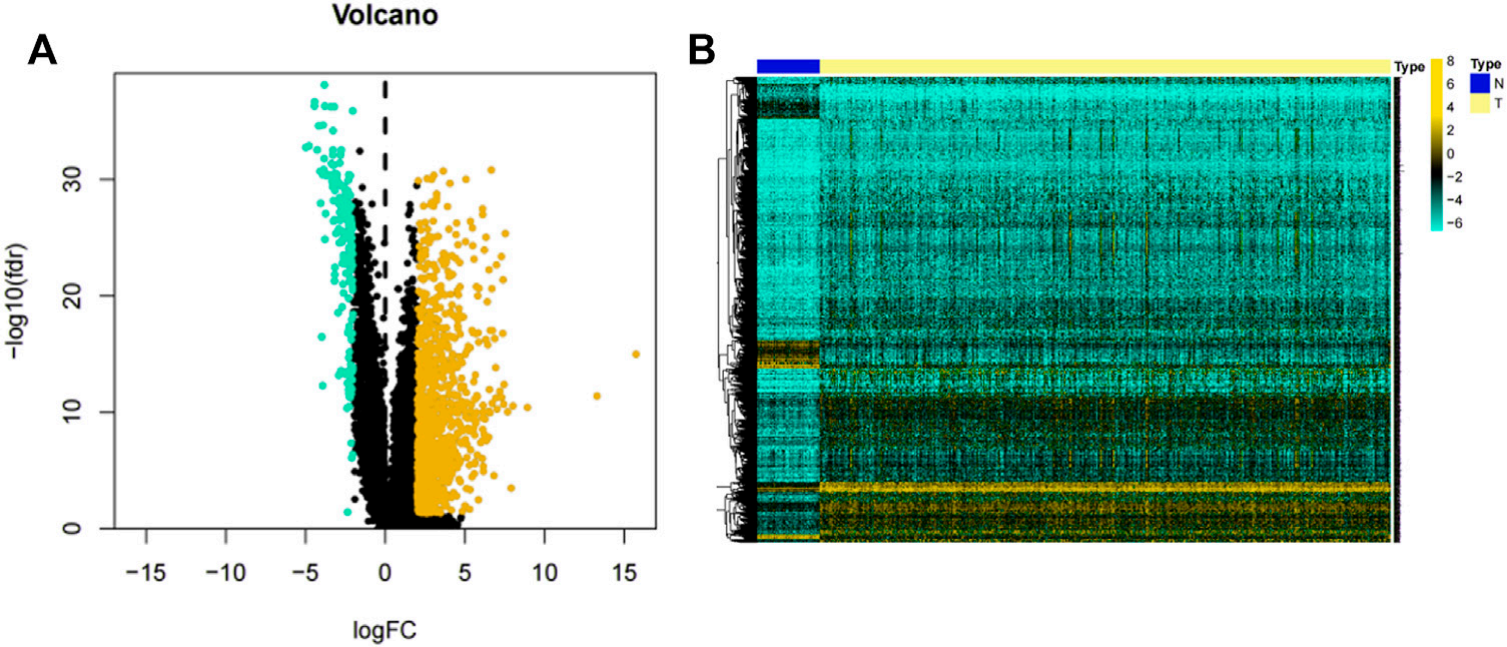
Volcano plot and heatmap of lncRNAs. **(A)** Volcano plot of 1,223 lncRNAs in the LUAD samples. Yellow dots represent 1,044 upregulated lncRNAs, while blue dots represent 179 downregulated lncRNAs. **(B)** Heatmap of 1,223 lncRNAs expression levels in LUAD samples from the TCGA-LUAD project. *N* = normal samples, *T* = LUAD samples.

The flow chart illustrated in Figure 1 shows the overall design of this study and some of the main results. After data collation, we obtained the expression data of 14,142 lncRNAs and 19,658 mRNAs for 535 LUAD samples and 59 normal samples from the TCGA-LUAD database. Through statistical comparison, 1,223 DELs in tumor samples and normal samples were identified with a log2 | fold change |> 2 and adjusted *p* < 0.05. Of these 1,223 DELs, 1,044 lncRNA were upregulated and 179 were downregulated in the LUAD patients. Next, volcano plots and heatmaps of the differential genes were drawn using the “plot” function and the “pheatmap” package in the R software, the results of which are illustrated in Figures 2A,B.

After the exclusion of 45 LUAD samples with incomplete survival data, 490 LUAD samples were finally enrolled in the study. In these 490 samples, 1,223 DELs were analyzed by the Kaplan-Meier method and univariate CPHR analysis, where OS served as the dependent variable and lncRNA expression as the independent variable. The results of the univariate CPHR analysis are depicted in Supplementary Table S1, and a total of 15 lncRNAs were found to be statistically significantly associated with OS in LUAD patients (all *p* < 0.01). Of this 15 lncRNA, the high expression of 13 lncRNAs (namely, LINC02081, AC010343.3, LINC02086, AC068228.1, AC022784.1, SATB2-AS1, AL138789.1, LINC01843, LINC00519, AL606489.1, DEPDC1-AS1, AC087588.2, and FAM83A-AS1) was associated with a shorter OS. In contrast, the high expression of AC026355.1 and AL031600.2 was associated with a higher OS. Moreover, as shown in Supplementary Figure S1, the results of the Kaplan-Meier analysis conformed to those of the univariate CPHR analyses. To this point, 15 lncRNAs with some correlation between the gene expression volume and prognosis were included as the candidate OS-related lncRNAs.

### Identification and evaluation of an OS-related lncRNA signature to predict the OS

After removing 11 samples without complete clinical features (such as TNM stage or age), 479 LUAD samples formed the “entire dataset”, of which 239 groups were randomly selected as the “primary dataset”. The differential analysis revealed no statistical differences in the baseline clinical risk factors and OS between the “entire” and “primary datasets” (all *p* > 0.05; Table 1).

**TABLE 1:** Baseline clinical characteristics and OS between the “entire dataset” and the “primary dataset”.

Character	Primary dataset	Entire dataset	*p*-value(t)
	*n* = 239	*n* = 479	
Age (year)			0.35
≥65	145 (60.67%)	266 (55.53%)	
<65	94 (39.33%)	213 (44.47%)	
Gender			0.80
Female	135 (56.49%)	260 (54.28%)	
Male	104 (43.51%)	219 (45.72%)	
TNM stage			0.68
I	130 (54.39%)	259 (54.07%)	
II-IV	109 (45.61%)	220 (45.93%)	
Tumor stage			0.80
TX	2 (0.84%)	3 (0.63%)	
T1-T2	212 (88.70%)	415 (86.64%)	
T3-T4	25 (10.46%)	61 (12.73%)	
Lymph node metastasis			0.78
NX	5 (2.09%)	9 (1.89%)	
No	152 (63.60%)	311 (64.92%)	
Yes	82 (34.31%)	159 (33.19%)	
Distant metastasis			0.80
MX	72 (30.13%)	139 (29.02%)	
No	156 (65.27%)	316 (65.97%)	
Yes	11 (4.60%)	24 (5.01%)	
OS (days)			0.85
Average	773	761	
Median	582	557	

Candidate prognosis lncRNAs were further screened by multivariate-CPHR analysis (stepwise model) in the “primary dataset” using AIC to avoid overfitting. Five OS-related lncRNAs were picked with the largest fit and the lowest AIC values (Table 2), namely, is AC068228.1, SATB2-AS1, LINC01843, AC026355.1, and AL606489.1. Next, these 5 OS-related lncRNAs and their risk coefficients were integrated into the predictive signature to obtain a risk scoring using the following formula:

$$Risk\;Score=\left(0.4537\times \mathrm{expression}\;\mathrm{AC}068228.1\right)+\left(2.1929\times expression\;\mathrm{SATB}2–\mathrm{AS}1\right)+\left(0.0824\times expression\;\mathrm{LINC}01843\right)+\left(–0.3963\times expression\;\mathrm{AC}026355.1\right)+\left(0.1383\times expression\;\mathrm{AL}606489.1\right)$$

**TABLE 2:** Five OS-related lncRNAs in the “primary dataset”.

lncRNA	Coefficients	HR (95% CI)	*p*-value
AC068228.1	0.4537	1.5741 (1.2184–2.0337)	<0.001
SATB2-AS1	2.1929	8.9619 (2.2255–36.0894)	0.002
LINC01843	0.0824	1.0859 (1.0109–1.1665)	0.023
AC026355.1	−0.3963	0.6727 (0.5325–0.8500)	<0.001
AL606489.1	0.1383	1.1483 (0.9741–1.3537)	0.099

Abbreviations: HR, hazard ratio; CI, confidence interval.

**FIGURE 3:**
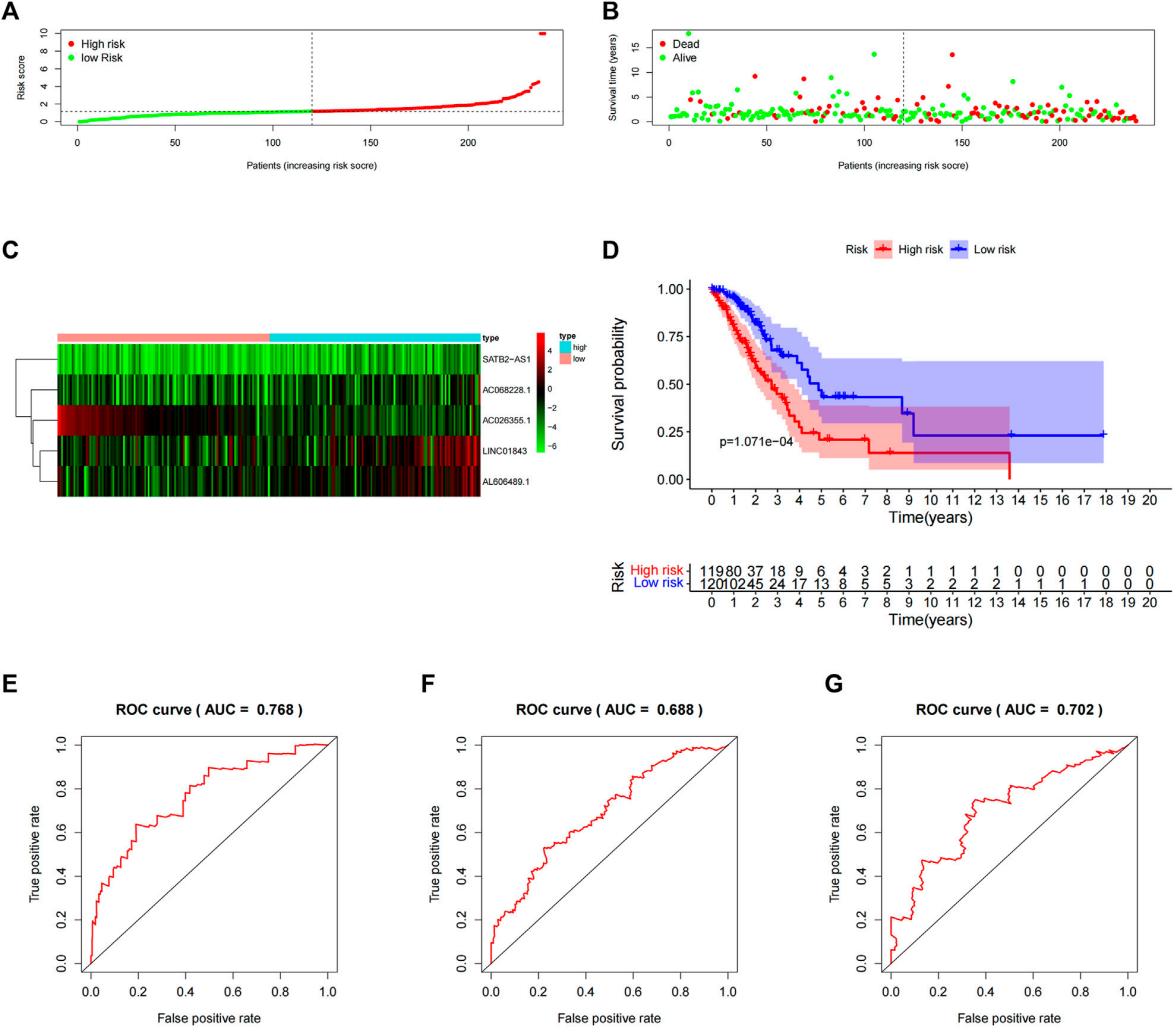
Assessment of the 5-lncRNA signature for predicting OS of LUAD in the “primary dataset”. **(A)** The risk score distribution in the “primary dataset”. **(B)** The OS status in the “primary dataset”. **(C)** The OS-related lncRNAs expression heatmaps of the 5-lncRNA signature in the “primary dataset”. **(D)** Kaplan–Meier curves comparing OS between the high-risk groups (*n* = 119) and low-risk groups (*n* = 120) in the “primary dataset”. Blue- and red-shaded sections indicate the confidence intervals for survival. Listed below the curve is the number of patients being at risk. **(E)** Time-dependent ROC curve based on 5-lncRNA signature predicting 1 year-OS in the “primary dataset”. **(F)** Time-dependent ROC curve based on 5-lncRNA signature predicting 3 years-OS in the “primary dataset”. **(G)** Time-dependent ROC curve based on 5-lncRNA signature predicting 5 years-OS in the “primary dataset”.

Next, we computed the risk score for LUAD patients in the “primary dataset” according to the 5 lncRNA signatures. Using the median risk score (0.09382005) as the cut-off value, 239 LUAD patients were classified into high- (*n* = 119) or low- (*n* = 120) risk groups. The risk score distributions, OS status, and the 5 lncRNA expression profiles in the “primary datasets” are depicted in Figure 3 (A–C). OS-related lncRNAs expression heatmaps revealed that the 4 upregulated lncRNA (i.e., AC068228.1, SATB2-AS1, LINC01843, and AL606489.1) demonstrated higher expression levels in the high-risk group, and the AC026355.1 expression levels were lower in the high-risk groups. As shown in Figure 3D, the Kaplan–Meier curve obviously showed that the OS time in the high-risk group was less than that in the low-risk group (*p* = 1.071E-04, log-rank test). Subsequently, in the “primary dataset”, as shown in Figures 3E–G, the AUC of the time-dependent ROC curve was 0.768 at 1 year, 0.668 at 3 years, and 0.702 at 5 years.

**FIGURE 4:**
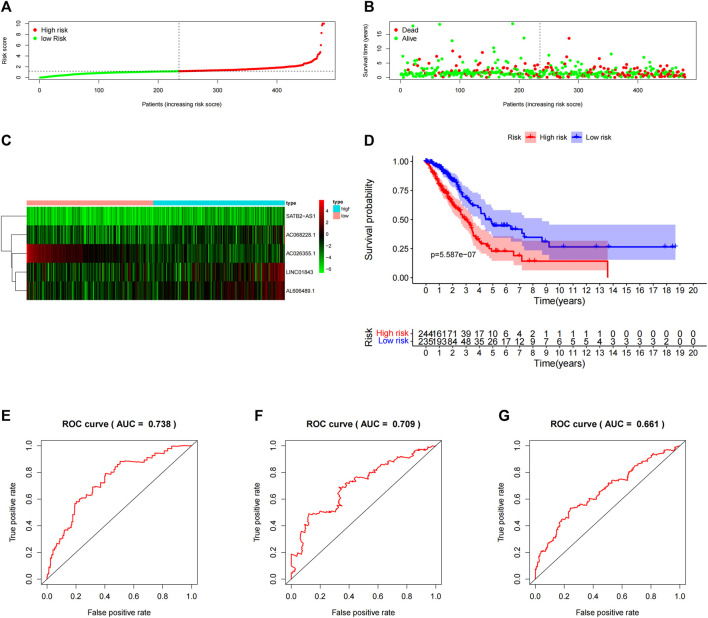
Assessment of the 5-lncRNA signature in the “entire dataset”. **(A)** The risk score distribution in the “entire dataset”. **(B)** The OS status in the “entire dataset”. **(C)** The OS-related lncRNAs expression heatmaps of the 5-lncRNA signature in the “entire dataset”. **(D)** The Kaplan–Meier curves comparing OS between the high-risk groups (*n* = 244) and the low-risk groups (*n* = 235) in the “entire dataset”. Blue- and red-shaded sections indicate the confidence intervals for survival. The number of patients at risk is listed below the curve. **(E)** Time-dependent ROC curve based on 5-lncRNA signature predicting 1 year-OS in the “entire dataset”. **(F)** Time-dependent ROC curve based on 5-lncRNA signature predicting 3 years-OS in the “entire dataset”. **(G)** Time-dependent ROC curve based on 5-lncRNA signature predicting the 5 years-OS in the “entire dataset”.

To verify the prediction of 5-lncRNA signatures obtained from the “primary dataset”, we applied 5-lncRNA signatures to the “entire dataset” (*n* = 479). Similarly, 479 patients were classified into the high-risk (*n* = 244) and low-risk (*n* = 235) groups according to the median risk score in the “primary dataset”. The risk score distributions, OS status, and the 5 lncRNA expression profiles in the “entire dataset” are depicted in Figures 4A–C. The results from the “entire dataset” are consistent with those from the “primary dataset”. Meanwhile, the Kaplan–Meier curve (Figure 4D) showed that the OS in the high-risk group (*n* = 244) was significantly shorter than that in the low-risk group (*n* = 235) (*p* = 5.587E-07, log-rank test). As shown in Figures 4E–G, the AUC of the time-dependent ROC curve was 0.738 at 1 year, 0.661 at 3 years, and 0.709 at 5 years. The 5-lncRNA signature showed a good prediction performance both in the “primary dataset” and the “entire dataset” of the LUAD patients. The prediction results of 5-lncRNA signature in “primary dataset” and the “entire dataset” were shown in Supplementary Table S2.

### The prognostic effect of the 5-lncRNA signature as an independent prognostic factor in LUAD patients.

Next, to examine whether the prognostic performance of the 5-lncRNA features was independent of other conventional clinical risk factors, we performed multivariate CPHR analyses. The hazard ratio (HR) in the “entire dataset” (Table 3) was 1.085 (*p* < 0.001, 95% CI = 1.052–1.118), and in the “primary dataset” (Supplementary Table S3) was 1.065 (*p* < 0.001, 95% CI = 1.028–1.102). The abovementioned data indicates that these 5 lncRNA signatures could independently predict the prognosis of LUAD patients as an independent prognostic factor for LUAD.

**TABLE 3:** Univariate and multivariate Cox proportional hazards regression analyses results of 5-lncRNA signature and other clinical risk factors in the “entire dataset”.

Characteristic	Univariate analysis		Multivariate analysis	
	HR (95%CI)	*p*-value	HR (95%CI)	*p*-value
Age	1.009 (0.992–1.025)	0.266	1.016 (1.000–1.033)	**0.049**
Gender (female vs. male)	1.009 (0.992–1.026)	0.855	0.923 (0.666–1.278)	0.631
TNM stage (I-Ⅳ)	1.659 (1.430–1.924)	**<0.001**	1.422 (1.138–1.776)	**0.001**
Tumor stage (T1-T4)	1.495 (1.223–1.828)	**<0.001**	1.149 (0.929–1.421)	0.199
Lymph node metastasis (N0-N3)	1.715 (1.435–2.049)	**<0.001**	1.221 (0.952–1.565)	0.114
Risk score	1.101 (1.070–1.133)	**<0.001**	1.085 (1.052–1.118)	**<0.001**

Notes: Bold values indicate statistical significance (*p* < 0.05). Abbreviations: HR, hazard ratio; CI, confidence interval.

**FIGURE 5:**
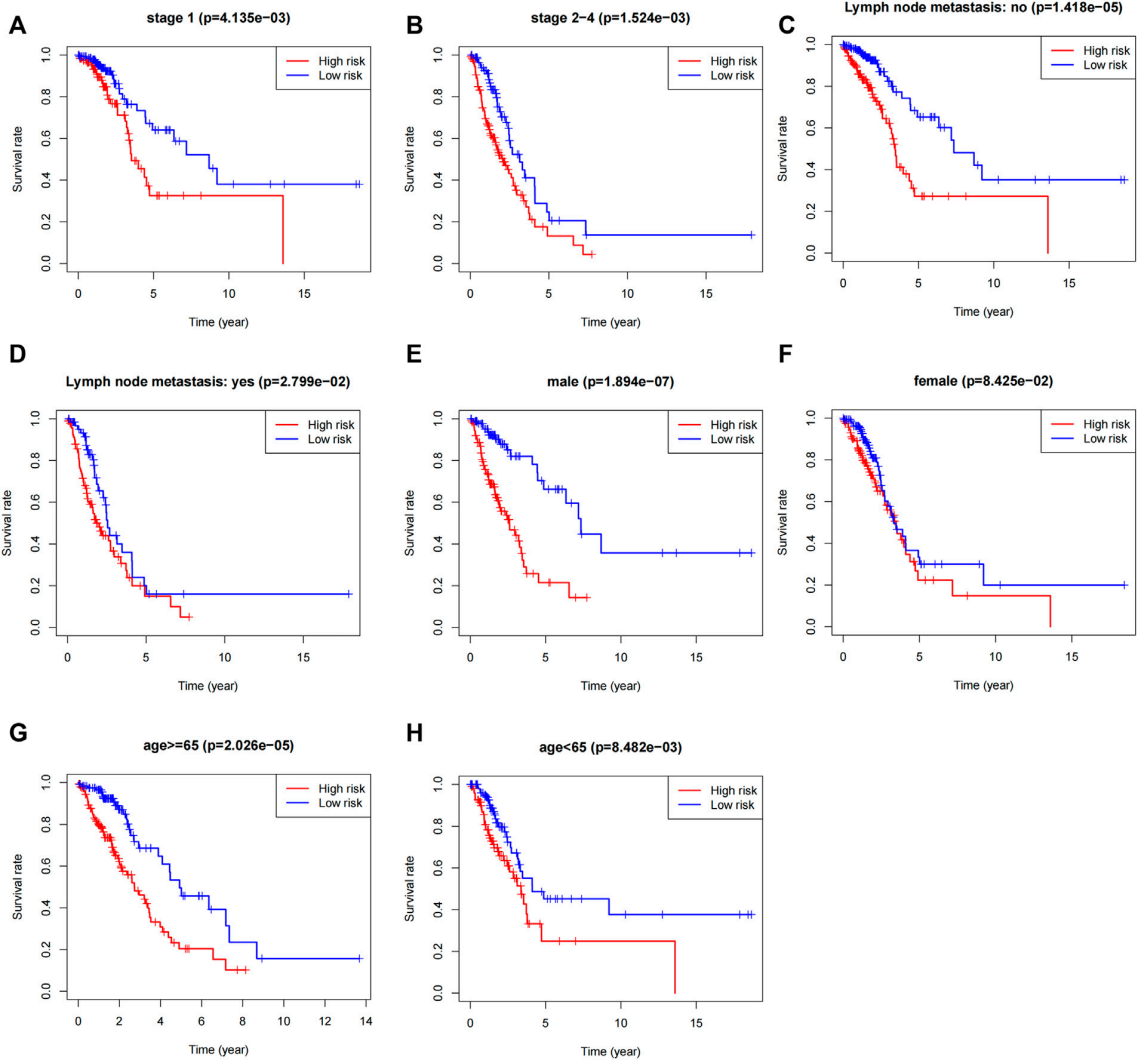
Stratified analysis of the 5-lncRNA signature in LUAD patients. **(A)** Kaplan-Meier analysis of patients in the stage I subgroup, **(B)** stage Ⅱ–IV subgroup, **(C)** without lymph node metastasis subgroup, **(D)** with lymph node metastasis subgroup, **(E)** male subgroup, **(F)** female subgroup, **(G)** age ≥65 years subgroups, and **(H)** age <65 years subgroups. The differences between the two risk groups were assessed by two-sided log-rank tests.

To validate the scope of applicability of the risk score prediction, we conducted a stratified analysis of the “entire dataset”. First, considering the number of people, 479 LUAD patients were classified into stage I (*n* = 259; Figure 5A) and stage Ⅱ–Ⅳ (*n* = 220; Figure 5B) based on the TNM stage. Each subgroup was classified as the high-risk and low-risk groups and then Kaplan–Meier curves were accordingly plotted. Second, 479 patients were classified into no (*n* = 311, Figure 5C) or yes (*n* = 159, Figure 5D) subgroups according to the absence or presence of lymphoid tract metastasis, respectively. Next, 479 patients with LUAD were assigned into male (*n* = 219, Figure 5E) and female subgroups (*n* = 260, Figure 5F). Then, 479 patients with LUAD were assigned to the age ≥ 65 years (*n* = 266, Figure 5G) and <65 years subgroups (*n* = 213, Figure 5H). Finally, we noted that, in all subgroups, the survival time was significantly lower in the high-risk group than that in the low-risk groups, albeit it was not statistically significant in the female (*p* = 0.08) subgroup. To further validate the association between OS and 5-lncRNA, GEO database (GSE3141 and GSE19188) was used to perform external validation. The Kaplan-Meier curves for OS associated with the SATB2-AS1 expression were shown in Supplementary Figure S2 (GSE3141: *p* = 0.6312, GSE19188: *p* = 0.0914, GSE3141 + GSE19188: *p* = 0.1322). It is a pity that all three statistics did not show a significant effect. However, SATB2-AS1 still showed a clear trend towards promoting oncogenes, which is consistent with our findings in the TCGA database. All case ID involved in this study were shown in Supplementary Table S4.

### Five-lncRNA signature-based signature has a better survival prediction effect than other clinical characters

**FIGURE 6:**
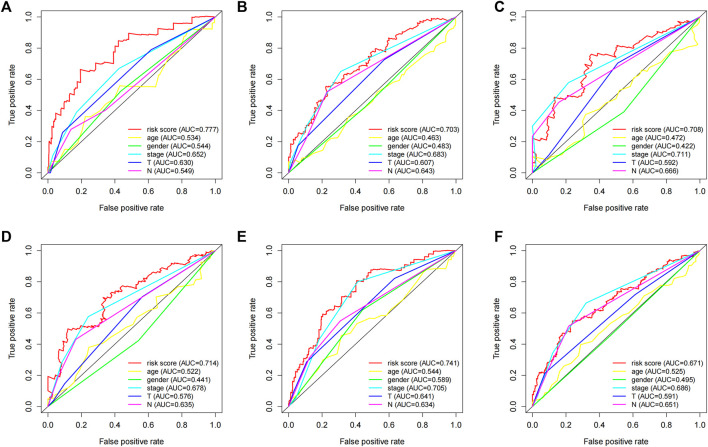
The prognostic value of the 5-lncRNA signature in comparison with other clinical factors. Time-dependent ROC curve analysis of the 5-lncRNA signature for predicting **(A)** 1 year-OS, **(B)** 3 years-OS, and **(C)** 5 years-OS in the “primary dataset”. Time-dependent ROC curve analysis of the 5-lncRNA signature for predicting **(D)** 1 year-OS, **(E)** 3 years-OS, and **(F)** 5 years-OS in the “entire dataset”.

We employed the time-dependent ROC curves to compare the predictive effects of different prognostic factors using the AUC as a comparison indicator. As shown in Figure 6, the stable predictive performance of the 5-lncRNA signature is more outstanding than the conventional clinical characters such as the TNM stage, and are efficient to predict the prognosis of LUAD patients.

### AL606489.1, an OS-related lncRNAs, demonstrating gender dimorphism

**FIGURE 7:**
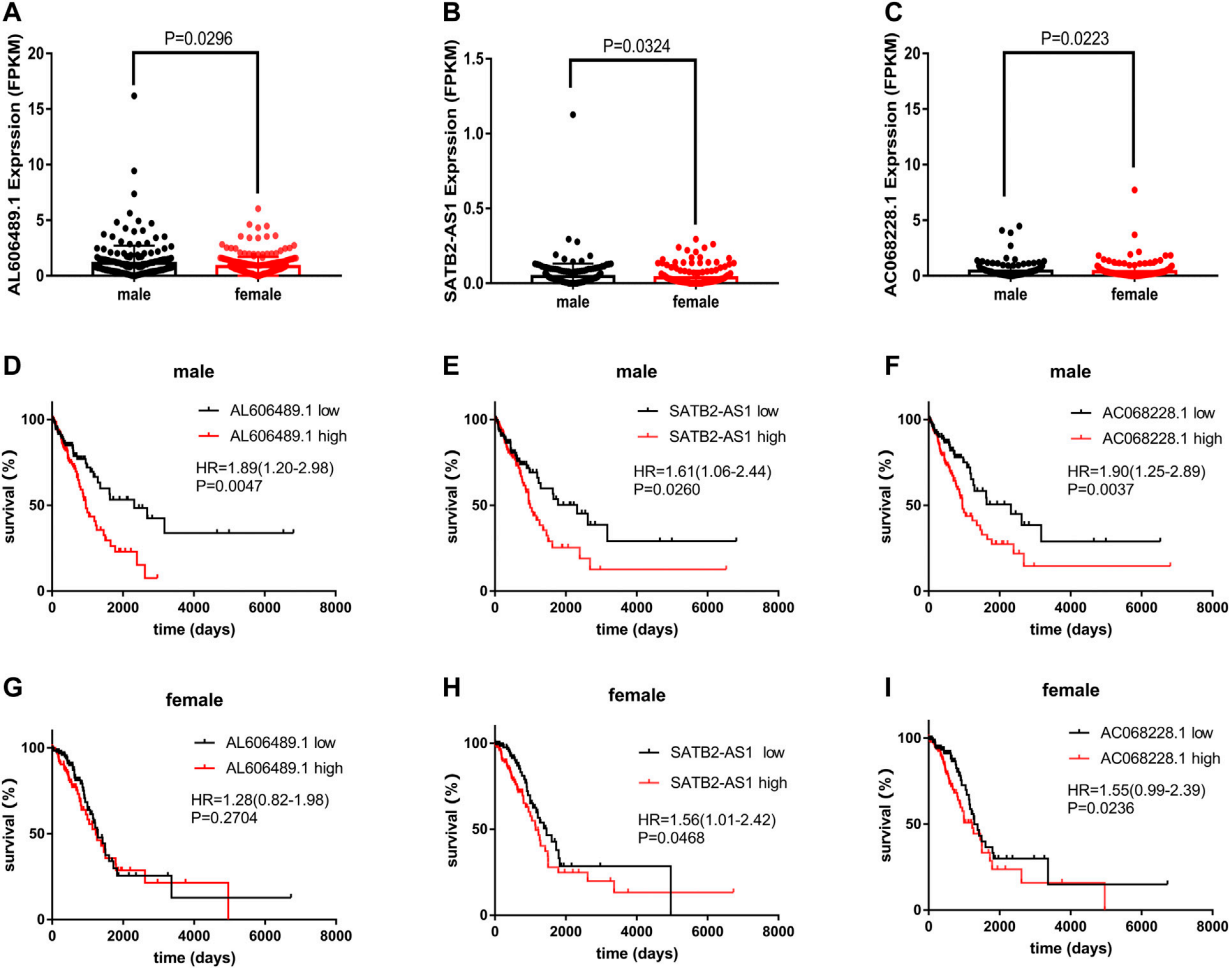
The expression of AL606489.1, SATB2-AS1 and AC068228.1 in LUAD. **(A)** Differentially expressed AL606489.1 between 260 female and 219 male tumor samples. **(B)** Differentially expressed SATB2-AS1 between 260 female and 219 male tumor samples. **(C)** Differentially expressed AC068228.1 between 260 female and 219 male tumor samples. Kaplan–Meier curves for OS associated with the AL606489.1 expression in **(D)** male and **(G)** female. Kaplan–Meier curves for OS associated with the SATB2-AS1 expression in **(E)** male and **(H)** female. Kaplan–Meier curves for OS associated with the AC068228.1 expression in **(F)** male and **(I)** female.

Among the 5 OS-related genes, AL606489.1, SATB2-AS1 and AC068228.1 was differentially expressed between males and females (Figures 7A–C. This significant difference was not shown in LINC01843 (*p* = 0.5833) and AC026355.1 (*p* = 0.5177), as shown in Supplementary Figures S3A,B. The Kaplan–Meier curves for the OS related with AL606489.1 expression in males (low = 109, high = 110) and females (low = 130, high = 130) are depicted in Figures 7D,G respectively. For SATB2-AS1, the Kaplan–Meier curves in males and females are depicted in Figures 7E,H respectively. For AC068228.1, the Kaplan–Meier curves in males and females are depicted in Figures 7F,I respectively. In males, the high expression of AL606489.1, SATB2-AS1 or AC068228.1 was associated with the shorter OS. In females, the high expression of SATB2-AS1 or AC068228.1 was associated with the shorter OS. Dissimilarly, the high expression of AL606489.1 in females was not significantly associated with the OS (*p* = 0.2704). Finally, to verify whether this discrepancy was attributable to AL606489.1 association with the gender, we noted no significant difference in the overall survival between males and females by the Kaplan-Meier analysis (Supplementary Figure S3).

### Functional characteristics of 5 OS-related lncRNAs

**FIGURE 8:**
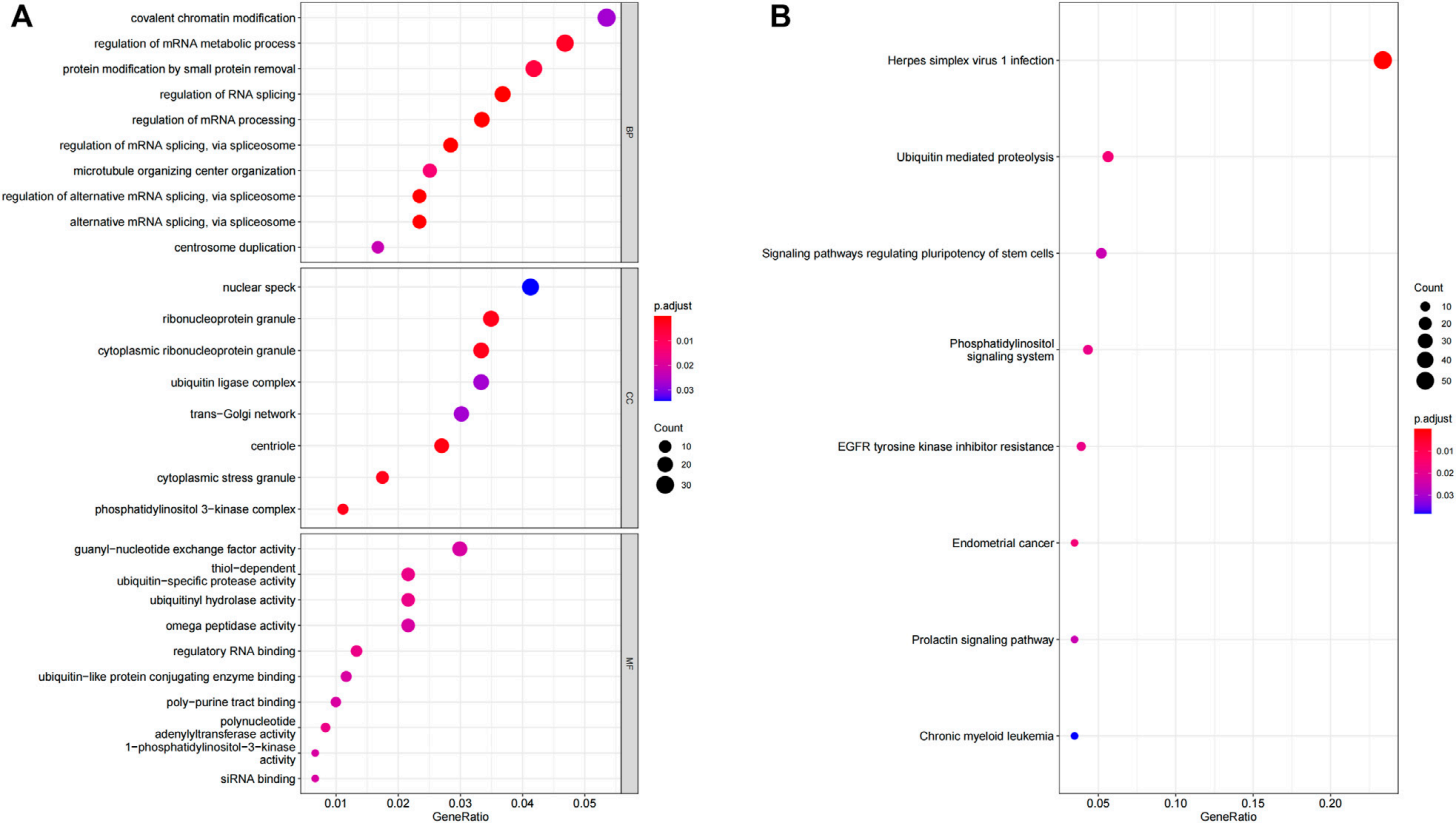
GO and KEGG functional enrichment analysis of the mRNA co-expressed with 5 OS-related lncRNA. **(A)** GO enrichment analysis. **(B)** KEGG enrichment analysis.

To determine the possible function of 5 OS-related lncRNAs in the tumorigenic development of LUAD tumors, we conducted an function enrichment analysis on mRNAs co-expressed with OS-associated lncRNAs in 490 LUAD samples. The levels of the 928 mRNA expressions were positively associated with the level of at least one OS-related lncRNA (co-expression coefficient >0.50). The GO analysis indicated that these co-expressed mRNAs were enriched in 52 GO terms (Supplementary Table S5). These GO terms were mainly enriched in regulating the mRNA metabolic processes, RNA splicing, and ubiquitin-specific protease activity (Figure 8A). Similar findings were obtained from the KEGG pathway enrichment analysis (Figure 8B), such as the ubiquitin-mediated proteolysis pathway. Therefore, the characteristics of 5-lncRNA mainly affected the gene expression within the nucleus and may be related to cell cycle regulation.

## Discission

LUAD is one of the most widely diagnosed subtypes of lung cancer (Fong et al., 1999). Owing to the unknown pathogenesis and unsatisfactory treatment effect, the mortality of LUAD patients remains high (Jiang et al., 2019). In recent years, lncRNA has been applied as a potential tumor marker with promising research progress in LUAD (Li et al., 2014).

In this study, both univariate and multivariate CPHR analyses were performed to establish a 5-lncRNA signature. This model showed high accuracy in both the “entire” and “primary datasets”. In contrast, our prognostic model outperformed the other prognostic features. Risk stratification analysis suggested that our prediction model applied to different subgroups. Finally, we employed GO and KEGG to detect the biological function of our predictive model. Our results seemingly explored how these 5 OS-related lncRNAs are involved in tumor progression. Finally, the lncRNA AL606489.1 showed a possible association with sex dimorphism.

Our prognostic model consisted of 5 LncRNAs, 4 (i.e., AC068228.1, SATB2-AS1, AC026355.1, AL606489.1) of which have been previously reported to be related to the prognosis of LUAD. For instance, SATB2-AS1 has been reported to promote tumor cell growth in osteosarcoma (Liu et al., 2017), and NSCLC (Wu et al., 2021). However, in colorectal cancer (Xu et al., 2019), SATB2-AS1 has the effect of inhibiting tumor cell metastasis. Similar to our result, AC026355.1 was reported to be an immune-related gene with tumor suppressor effects by Li et al. (Li et al., 2020) In past studies, AL606489.1 has been reported to be associated with autophagy (Liu et al., 2021), ferroptosis (Guo et al., 2021), cuproptosis (Mo et al., 2022) and pyroptosis (Li et al., 2018; Song et al., 2021) processes in LUAD tumor cells. LINC 01843 was first shown to be associated with LUAD progression. These reports provide a new direction for gene sequence studies in LUAD.

In the GO and KEGG analysis results, the mRNAs co-expressed with prognostic-associated lncRNAs were associated with processing and RNA transport in the nucleus, such as in the regulation of mRNA metabolic process and the regulation of RNA splicing. Past studies have demonstrated that one of the prognostic-related genes, SATB2-AS1, acts as a miR-299-3p sponge, promoted the development of NSCLC. The underlying mechanism is the promotion of tumor cell proliferation, cell cycle progression, and survival (Wu et al., 2021). Thus, the results of GO and KEGG seem to appropriately reflect the place of action that was associated with prognosis, lncRNA affects the prognostic effect in patients with LUAD.

In the risk stratification analysis, this predictive model showed a slightly better performance in male patients (*p* < 0.05) than in female patients (*p* = 0.08), which prompted us to further explore the reasons for this discrepancy.

In our study, AL606489.1 was highly expressed in males relative to that in females. Moreover, on the premise that there is no significant difference in the prognosis between males and females with LUAD, AL606489.1 exhibited high levels of OS association in male patients, while showing no significant OS association in female patients. Therefore, we suggest that AL606489.1 demonstrates a gender dimorphism in terms of the prognostic effects in patients with LUAD. Meanwhile, this difference of AL606489.1 expression in females compared to males may be why the 5-lncRNA signature did not show significance in females in Figure 5F.

A person’s gender is one of the key factors affecting the occurrence and development of cancer throughout his or her lifetime. In addition to the sex-specificity of ovarian cancer in women and prostate cancer in men, several tumors are associated with a significant sex bias in terms of incidence (Li et al., 2018), metastatic (Kim et al., 2020), prognosis (Song et al., 2021), and therapeutic efficacy (Freudenstein et al., 2020). As the attention to gender differences has increased, gender dimorphism has been mentioned in increasing studies (Yuan et al., 2016).

In LUAD, sex bias is also associated with patients’ acquired behavior. For instance, Henschke et al. reported that women smoking was associated with a higher risk of lung cancer compared to men smoking, but after diagnosis of lung cancer, they had better survival rates (Henschke et al., 2006). The difference in prognosis between male and female patients may be related to natural differences in hormone levels. Multiple studies have demonstrated that sexual dimorphism may be due to differences in the estrogen content between men and women, which develops into different prognostic effects between male and female patients with LUAD. For example, LncRNA LINC00263 has been implicated as an oncogene in men and estrogen by Liu et al. (Liu et al., 2020). However, the specific role of lncRNA in sex dimorphism has not been well studied. In the present case, AL606489.1 can hence be a breakthrough.

In our study, the action mechanism of AL606489.1 was explored by co-expression analyses. In the co-expression analysis, AL606489.1 was found to be highly correlated with the sarcolemmal membrane-associated protein (SLMAP) expression (correlation coefficient = 0.64) (Supplementary Table S5). A subform of the SLMAP has been reported to be a component of the microtubule (Mt) tissue center (Guzzo et al., 2004). Mts is an important therapeutic target for tumor cells (Dumontet and Jordan, 2010). Clinically, some compounds that break Mt dynamics are also some of the most effective chemotherapeutics for cancer, such as vincristine alkaloids and taxanes (Checchi et al., 2003). Similarly, the mt-targeted drugs (MTDs) form a major family of anticancer drugs with anti-mitotic and antiangiogenic properties that inhibit tumor progression, mainly by changing the Mt dynamics of the tumor and endothelial cells (Bhat and Setaluri, 2007). However, there are no reliable markers that can be used for the prediction of the development of cancer sensitivity and resistance during treatment. In this study, AL606489.1 was found to be highly co-expressed with SLMAP and highly correlated with LUAD prognosis, indicating its potential as a reliable marker. Alternatively, the differential expression of AL606489.1 in males and females may be responsible for the clinical emergence of sex-differential efficacy of anticancer drugs that disrupt the Mt dynamics (Moore et al., 2003).

The limitations of the present study include the lack of external validation considering that the most lncRNAs required in this study were inaccessible in the GEO database. Second, as RNA testing in the TCGA database is constantly updated, this study is slightly sample-limited. Finally, the preliminary conclusion that AL606489.1 demonstrates sexual dimorphism, as derived in this study, needs to be further validated through *in vitro* and *in vivo* biological experiments, if the external conditions support it.

## Conclusion

Our 5-lncRNA signature (composed of AC068228.1, SATB2-AS1, LINC01843, AC026355.1, and AL606489.1) could effectively predict the OS of LUAD patients, indicating its positive role in early screening and prognosis prediction of LUAD. Moreover, AL606489.1 demonstrated gender dimorphism, thereby providing a new direction for mechanistic studies on sexual dimorphism.

## Data Availability

Publicly available datasets were analyzed in this study. This data can be found here: https://portal.gdc.cancer.gov/. https://www.ncbi.nlm.nih.gov/geo/.

## Abbreviations

LUAD, lung adenocarcinoma; lncRNAs, long noncoding RNAs; OS, overall survival; GO, Gene Ontology; KEGG, Kyoto Encyclopedia of Genes and Genomes; TCGA, The Cancer Genome Atlas; DEL, differentially expressed lncRNA; FDR, False Discovery Rate; CPHR, Cox proportional hazards regression; AIC, Akaike information criterion; ROC, receiver operating characteristic; AUC, area under the time-dependent ROC curve; HR, hazard ratio; CI, confidence interval.
