# Supplementary material for: An efficient five-lncRNA signature for lung adenocarcinoma prognosis, with AL606489.1 showing sexual dimorphism
Source: Front Genet. 2022 Nov 30;13:1052092. doi: 10.3389/fgene.2022.1052092 (PMC9748423; doi:10.3389/fgene.2022.1052092)
Supplement: Supplementary file 1 [file DataSheet1.docx]

**
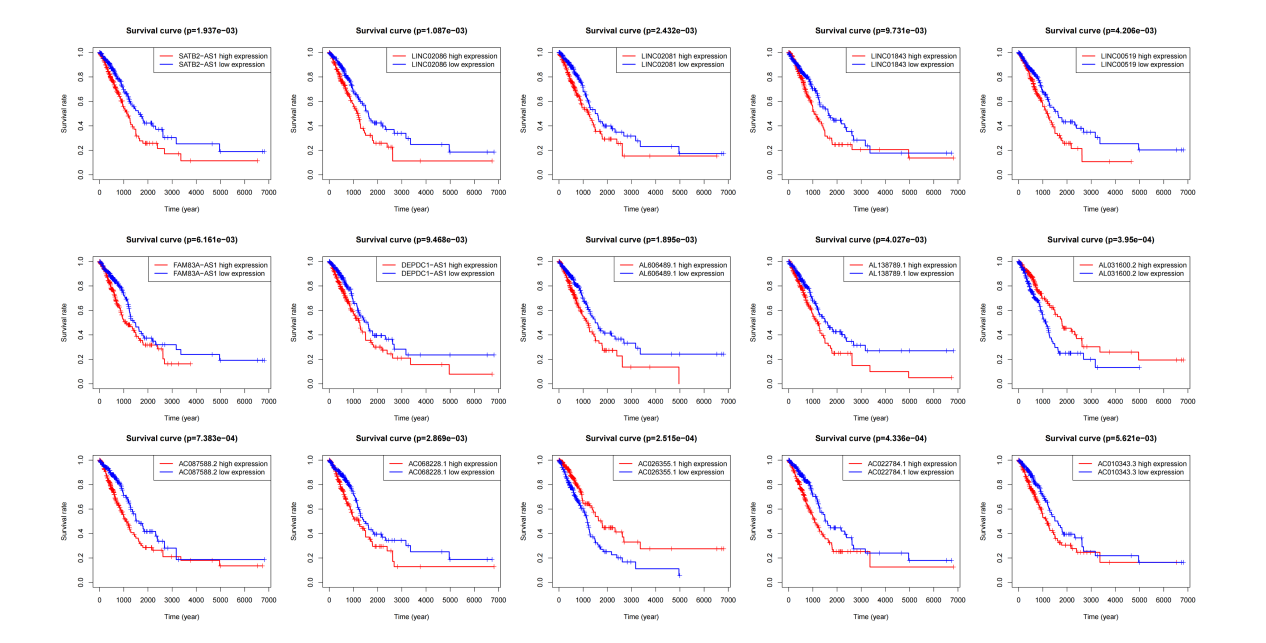
Supplementary Figure 1. the Kaplan-Meier analysis curves of 15 candidate OS-related lncRNA in the entire datase.**


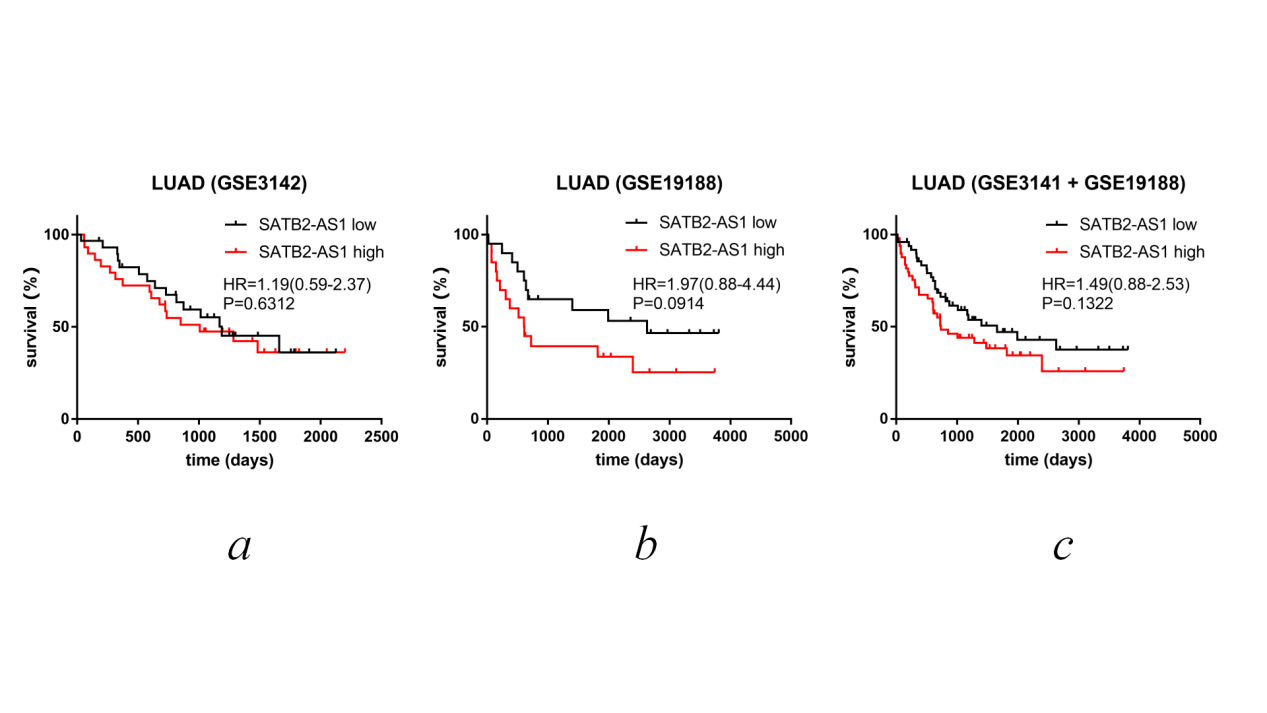


**Supplementary Figure 2. the Kaplan-Meier analysis curves of SATB2-AS1 in GEO datase. (A)** Kaplan–Meier curves for OS associated with the SATB2-AS1 expression in GSE3141 (low=29, high=29). **(B)** Kaplan–Meier curves for OS associated with the SATB2-AS1 expression in GSE19188 (low=20, high=20). **(C)** Kaplan–Meier curves for OS associated with the SATB2-AS1 expression in GSE3141 plus GSE19188 (low=49, high=49).


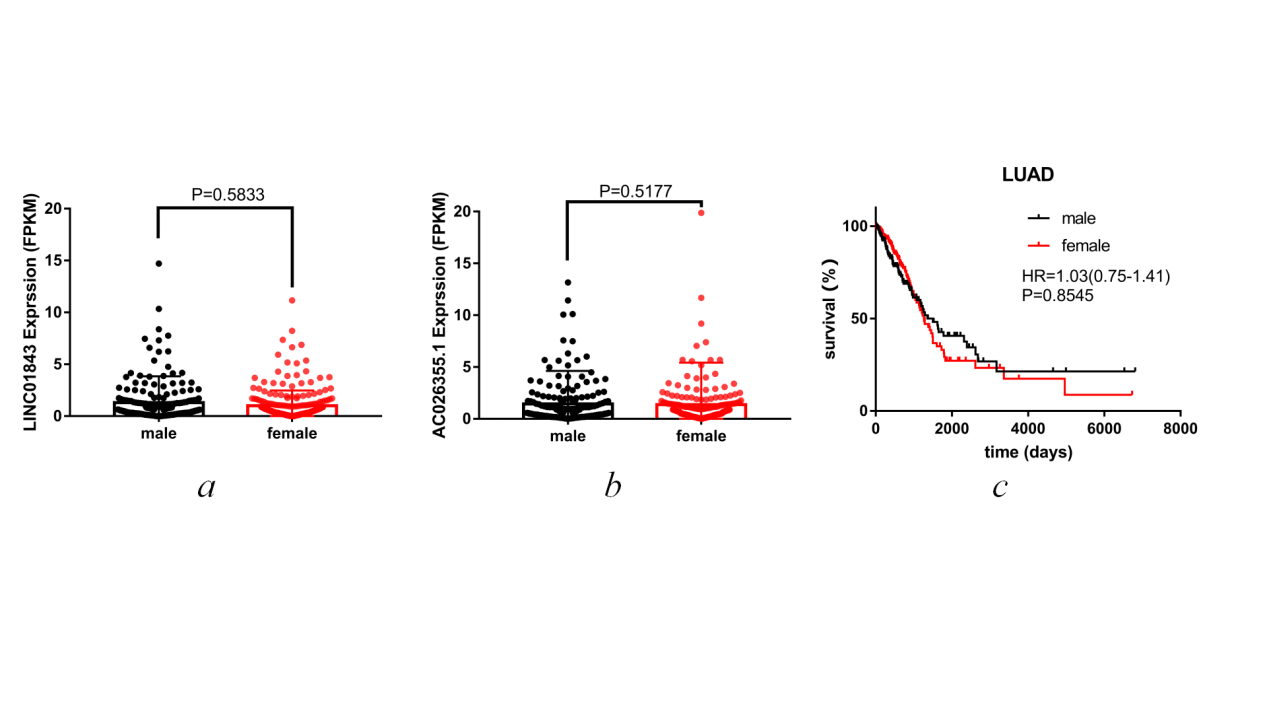


**Supplementary Figure 3. (A)** Differentially expressed LINC01843 between 260 female and 219 male tumor samples. **(B)** Differentially expressed AC026355.1 between 260 female and 219 male tumor samples. **(C)** Kaplan–Meier curves for the OS associated with gender.

**Supplementary Table 1. Univariate Cox proportional hazards regression analysis of 15 candidate OS-related lncRNA in the entire dataset.**

| Id | HR | Lower CI | Higher CI | P value |
| --- | --- | --- | --- | --- |
| LINC02081 | 1.1187 | 1.0329 | 1.2117 | 0.0058 |
| AC010343.3 | 1.4322 | 1.1804 | 1.7377 | 0.0002 |
| LINC02086 | 1.2027 | 1.0824 | 1.3364 | 0.0006 |
| AC068228.1 | 1.5809 | 1.2563 | 1.9894 | <0.0001 |
| AC022784.1 | 1.0236 | 1.007 | 1.0403 | 0.0044 |
| SATB2-AS1 | 9.5654 | 2.5295 | 36.1719 | 0.0008 |
| AL138789.1 | 1.1395 | 1.0476 | 1.2395 | 0.0023 |
| LINC01843 | 1.1057 | 1.0466 | 1.1682 | 0.0003 |
| LINC00519 | 1.1638 | 1.0610 | 1.2766 | 0.0013 |
| AC026355.1 | 0.7433 | 0.6293 | 0.8778 | 0.0004 |
| AL606489.1 | 1.1909 | 1.0864 | 1.3055 | 0.0002 |
| DEPDC1-AS1 | 84.9598 | 7.7797 | 927.8117 | 0.0003 |
| AC087588.2 | 1.3927 | 1.1478 | 1.6899 | 0.0008 |
| AL031600.2 | 0.0682 | 0.0113 | 0.4098 | 0.0033 |
| FAM83A-AS1 | 1.0348 | 1.0141 | 1.0558 | 0.0009 |

Abbreviations: HR, hazard ratio; CI, confidence interval.

**Supplementary Table 2. The prediction results of 5-lncRNA signature in "primary dataset" and the "entire dataset".**

| **TCGA Case ID** | **"primary dataset" or not** | **OS (days)** | **fustat** | **risk score** | **expression values for 5 OS-related LncRNAs** | | | | |
| --- | --- | --- | --- | --- | --- | --- | --- | --- | --- |
|  |  |  |  |  | **AC068228.1** | **SATB2-AS1** | **LINC01843** | **AC026355.1** | **AL606489.1** |
| TCGA-44-4112 | YES | 808 | 1 | -22.52519446 | 0.3148958 | 0 | 0.5746634 | 58.0215 | 2.013778 |
| TCGA-44-2662 | NOT | 1280 | 0 | -14.41084951 | 0.4081176 | 0.04256475 | 0.2864565 | 38.3554 | 3.523415 |
| TCGA-44-3917 | YES | 1183 | 0 | -6.902247011 | 0.1787545 | 0.0621442 | 1.672897 | 19.87556 | 4.477323 |
| TCGA-62-8399 | NOT | 2696 | 0 | -5.031820638 | 0.1714276 | 0.004256927 | 0.1890814 | 13.16108 | 0.5872982 |
| TCGA-35-3615 | NOT | 14 | 0 | -4.341122345 | 0.1781676 | 0.03378554 | 0.3524286 | 11.42737 | 0.02589529 |
| TCGA-49-6743 | NOT | 1621 | 0 | -4.108458077 | 0.4864918 | 0.02255058 | 0.9333435 | 11.67713 | 1.244458 |
| TCGA-86-8674 | YES | 806 | 0 | -3.7328453 | 0.3967569 | 0.01724162 | 0.02320687 | 10.06593 | 0.2643005 |
| TCGA-55-7995 | YES | 889 | 0 | -3.405425121 | 0.01780321 | 0.003094652 | 0.6872812 | 9.190643 | 1.195453 |
| TCGA-44-2655 | NOT | 1324 | 0 | -2.683547069 | 0.2572455 | 0.004968425 | 0.882737 | 7.396648 | 0.3427293 |
| TCGA-05-4384 | YES | 426 | 0 | -2.66852466 | 0.3012768 | 0.03273099 | 0.8194279 | 7.577085 | 0.421462 |
| TCGA-44-6146 | NOT | 728 | 0 | -2.524477936 | 0.1192262 | 0.01036228 | 1.171583 | 10.10031 | 9.435431 |
| TCGA-MP-A5C7 | NOT | 2248 | 0 | -2.491059914 | 0.1293043 | 0.01498426 | 0.347907 | 7.045986 | 1.309273 |
| TCGA-78-7156 | NOT | 976 | 1 | -2.426988478 | 0.03336814 | 0 | 0.02342099 | 6.316128 | 0.4267826 |
| TCGA-67-3773 | YES | 427 | 0 | -2.115647146 | 0.04333232 | 0.02259679 | 0.2433186 | 5.563737 | 0 |
| TCGA-05-4382 | NOT | 607 | 0 | -1.846680188 | 0.2261914 | 0.09583723 | 1.468559 | 6.006813 | 0.7232541 |
| TCGA-55-A492 | YES | 596 | 0 | -1.842041559 | 0.2378222 | 0.00918657 | 0.7048016 | 5.176718 | 0.1689875 |
| TCGA-64-1677 | NOT | 628 | 1 | -1.824293993 | 0.3914426 | 0.02916116 | 2.041018 | 5.699591 | 0.1788071 |
| TCGA-55-A48Y | NOT | 630 | 0 | -1.725224069 | 0.6211111 | 0 | 0.6435542 | 5.676765 | 1.371301 |
| TCGA-44-2656 | YES | 1429 | 0 | -1.696651146 | 0.6757191 | 0.00903517 | 0.2553843 | 5.607423 | 1.288069 |
| TCGA-44-A47A | YES | 466 | 0 | -1.658649089 | 0.1500518 | 0.1408473 | 0.442348 | 5.680549 | 1.295448 |
| TCGA-78-8640 | YES | 7062 | 0 | -1.622754533 | 0.175187936 | 0.030452131 | 0.073778334 | 4.498718767 | 0.056016883 |
| TCGA-73-4676 | NOT | 281 | 1 | -1.57827293 | 0.518168218 | 0.063579424 | 0.320912407 | 5.164338941 | 0.487311432 |
| TCGA-55-6972 | YES | 1632 | 1 | -1.489132527 | 0.1100045 | 0 | 0.1930296 | 4.0771 | 0.4396784 |
| TCGA-55-6987 | YES | 2137 | 0 | -1.364516552 | 0.017116085 | 0.008925636 | 0.204233393 | 3.602338134 | 0.136823025 |
| TCGA-64-1676 | NOT | 1728 | 0 | -1.334283036 | 0.2034982 | 0 | 0.119029 | 3.681342 | 0.162673 |
| TCGA-55-7727 | YES | 119 | 0 | -1.324646495 | 0.2800498 | 0.02920788 | 0.3145058 | 4.077659 | 0.5372808 |
| TCGA-86-8278 | YES | 944 | 0 | -1.324531856 | 0.209777699 | 0.002604622 | 0.347071234 | 3.986669711 | 0.910331619 |
| TCGA-62-A46V | YES | 2199 | 0 | -1.313606871 | 0.1161816 | 0.08583011 | 0.9377962 | 4.344488 | 0.6501148 |
| TCGA-50-5068 | YES | 1499 | 1 | -1.25751292 | 0.2687501 | 0.05450156 | 0.6287829 | 3.913129 | 0 |
| TCGA-86-8673 | NOT | 862 | 0 | -1.215322354 | 0.141700509 | 0.024631162 | 0.099459165 | 3.563755737 | 0.509728221 |
| TCGA-71-6725 | YES | 256 | 0 | -1.16311165 | 0.294858021 | 0.01507466 | 3.688757045 | 4.132562669 | 0.02772993 |
| TCGA-49-4486 | NOT | 2318 | 1 | -1.150714275 | 0.07475909 | 0.005198015 | 0.5142372 | 3.166627 | 0.1195223 |
| TCGA-05-5423 | NOT | 151 | 0 | -1.084161605 | 0.2268196 | 0.03285586 | 2.12272 | 3.702917 | 0.2417542 |
| TCGA-55-7911 | NOT | 537 | 0 | -1.077069594 | 0.4771704 | 0.1607048 | 5.923977 | 5.684588 | 0.8582441 |
| TCGA-67-3770 | YES | 610 | 0 | -1.070462122 | 0.04660008 | 0.01620057 | 2.387718 | 3.392597 | 0.1490052 |
| TCGA-MN-A4N4 | YES | 1175 | 0 | -1.049153392 | 1.117226 | 0.009710119 | 0.09802221 | 4.140779 | 0.4018911 |
| TCGA-69-7760 | NOT | 202 | 0 | -1.045048414 | 0.153416214 | 0 | 0.473802487 | 3.167953347 | 0.73582962 |
| TCGA-95-7948 | YES | 476 | 0 | -1.042424858 | 0.01995719 | 0.01734534 | 0.2241263 | 2.985113 | 0.5424168 |
| TCGA-53-7626 | NOT | 929 | 1 | -1.037199975 | 0.1759869 | 0.0183546 | 0.2717543 | 3.074943 | 0.2813617 |
| TCGA-78-7149 | YES | 3940 | 0 | -1.02429594 | 0.1099732 | 0.01911615 | 0.1157848 | 2.947778 | 0.3076874 |
| TCGA-L9-A444 | NOT | 307 | 0 | -0.974399601 | 0.3219237 | 0.05595855 | 0 | 3.249194 | 0.3216753 |
| TCGA-J2-A4AD | YES | 550 | 1 | -0.968792601 | 0.2621931 | 0.02900281 | 0.2007628 | 3.439046 | 1.409987 |
| TCGA-44-3398 | NOT | 1163 | 0 | -0.914594639 | 0.124913 | 0.007237686 | 1.154401 | 2.893533 | 0.4659819 |
| TCGA-55-7994 | YES | 603 | 0 | -0.905612934 | 0.700874121 | 0.115061475 | 3.867206448 | 4.973678974 | 1.276162945 |
| TCGA-64-1680 | YES | 1126 | 0 | -0.858949473 | 0.07462265 | 0.03891396 | 1.230869 | 2.765742 | 0.1193041 |
| TCGA-97-8179 | NOT | 435 | 0 | -0.843840277 | 0.214432 | 0 | 0.03344648 | 2.554553 | 0.4951937 |
| TCGA-44-6147 | NOT | 845 | 0 | -0.83493412 | 0.127456835 | 0.038771685 | 0.581500222 | 2.783748474 | 0.56037735 |
| TCGA-O1-A52J | NOT | 1798 | 1 | -0.82772529 | 0.1623277 | 0.004702782 | 1.063415 | 2.793308 | 0.7785717 |
| TCGA-86-A4JF | NOT | 737 | 1 | -0.772762173 | 0.1779758 | 0.0132586 | 0.678141 | 2.524104 | 0.4471368 |
| TCGA-44-5643 | NOT | 1013 | 0 | -0.749071335 | 0.06989944 | 0.006075152 | 0 | 2.081801 | 0.2235056 |
| TCGA-91-8496 | NOT | 505 | 0 | -0.732190464 | 0 | 0 | 0.1322937 | 1.896105 | 0.06026715 |
| TCGA-67-6217 | NOT | 422 | 0 | -0.721362195 | 0.131822337 | 0.051556689 | 0.277577117 | 2.44286778 | 0.368817744 |
| TCGA-73-7498 | YES | 1189 | 0 | -0.72123054 | 0.4582539 | 0 | 0.1952857 | 2.51298 | 0.3663203 |
| TCGA-91-6849 | YES | 35 | 0 | -0.686736421 | 0.107206331 | 0.018635195 | 0.188119511 | 2.057649288 | 0.171397777 |
| TCGA-97-A4M2 | YES | 624 | 0 | -0.661170074 | 0.13594201 | 0.004726038 | 0.419836082 | 2.195312924 | 0.738953912 |
| TCGA-86-A4P7 | NOT | 415 | 0 | -0.634190675 | 0.029166565 | 0.005069893 | 0.655101604 | 2.123395505 | 0.932609923 |
| TCGA-97-7937 | YES | 564 | 0 | -0.591355616 | 0.4566284 | 0.008355122 | 0.1518187 | 2.226846 | 0.3842324 |
| TCGA-62-A472 | YES | 910 | 0 | -0.585268911 | 0.5907883 | 0.1026941 | 0.7464105 | 3.206259 | 0.9445319 |
| TCGA-55-6979 | YES | 237 | 1 | -0.568764693 | 0.390383097 | 0.015079675 | 0.152227073 | 2.239216162 | 0.69347889 |
| TCGA-05-4424 | YES | 913 | 0 | -0.559508249 | 0.2687132 | 0.01556973 | 0.419131 | 1.976056 | 0.2386718 |
| TCGA-78-7167 | NOT | 2681 | 1 | -0.558970135 | 0.4689988 | 0.08433512 | 0.2610808 | 2.67588 | 0.5946841 |
| TCGA-64-5781 | NOT | 1559 | 0 | -0.550100314 | 0.2936911 | 0.01276275 | 1.932571 | 2.381111 | 0.528236 |
| TCGA-97-7546 | YES | 1285 | 0 | -0.541091935 | 0.027793434 | 0.004831208 | 0.331638185 | 1.508373817 | 0.044435182 |
| TCGA-50-5049 | NOT | 3094 | 0 | -0.537439459 | 0.134739705 | 0.017565898 | 0.236433492 | 1.694346047 | 0.107708591 |
| TCGA-MP-A4TK | NOT | 582 | 1 | -0.523717638 | 0.424940519 | 0.024621833 | 1.014099244 | 2.249940601 | 0.271752085 |
| TCGA-49-AARQ | NOT | 6732 | 0 | -0.515851573 | 0.214579 | 0.02131388 | 0.2581926 | 1.907084 | 0.5390965 |
| TCGA-50-5051 | YES | 478 | 1 | -0.510238737 | 0.8723103 | 0.006892263 | 0.4452887 | 2.571742 | 0.4437426 |
| TCGA-44-7659 | YES | 691 | 0 | -0.506116262 | 0 | 0.098943491 | 0.245863335 | 2.144445554 | 0.770030305 |
| TCGA-67-6215 | NOT | 174 | 0 | -0.473091584 | 0.1282478 | 0.008359775 | 0.2250418 | 1.782469 | 0.9995608 |
| TCGA-78-8655 | YES | 2360 | 0 | -0.472807335 | 0 | 0.136954155 | 0.331807627 | 2.085809446 | 0.18894601 |
| TCGA-49-AAQV | YES | 677 | 1 | -0.470526363 | 0.5275102 | 0 | 0.4113976 | 2.094764 | 0.6247149 |
| TCGA-MP-A4TC | NOT | 74 | 1 | -0.437907887 | 0.428138657 | 0.023256693 | 0.225381935 | 2.054354422 | 0.812835776 |
| TCGA-69-A59K | YES | 591 | 0 | -0.436627399 | 0.2085077 | 0.04077445 | 0.6585792 | 1.862983 | 0.458363 |
| TCGA-35-5375 | YES | 264 | 0 | -0.436193796 | 0 | 0 | 0 | 1.130446 | 0.08533589 |
| TCGA-55-8301 | YES | 534 | 0 | -0.422918861 | 0.151232054 | 0 | 0.300756429 | 1.401279626 | 0.28208201 |
| TCGA-97-8177 | YES | 499 | 0 | -0.402512332 | 0.018276452 | 0 | 0.5002993 | 1.354761466 | 0.613614954 |
| TCGA-MP-A4TH | NOT | 741 | 0 | -0.402124996 | 0.2101416 | 0 | 0.5162422 | 2.051431 | 1.973807 |
| TCGA-J2-8192 | YES | 739 | 0 | -0.391860511 | 0.161066117 | 0.025197659 | 0.972245714 | 1.577679657 | 0.180254858 |
| TCGA-86-8280 | NOT | 701 | 0 | -0.359510089 | 0.04348047 | 0.007558015 | 0.8697862 | 1.410078 | 0.6603932 |
| TCGA-55-8208 | NOT | 674 | 0 | -0.355573811 | 0.071217272 | 0.012379378 | 0.649833479 | 1.28834107 | 0.303625899 |
| TCGA-55-8514 | NOT | 520 | 0 | -0.353078449 | 0.329300281 | 0.012720175 | 0.256816539 | 1.452960353 | 0.175491302 |
| TCGA-67-6216 | YES | 141 | 0 | -0.349176575 | 0.161461168 | 0.011226428 | 0.113329113 | 1.367826399 | 0.619532482 |
| TCGA-MP-A4TD | NOT | 307 | 1 | -0.347240981 | 0.6033443 | 0.00998825 | 0.6856433 | 2.357874 | 1.699544 |
| TCGA-55-8508 | NOT | 617 | 0 | -0.343241898 | 0.4653791 | 0.008988307 | 0.3266482 | 1.574255 | 0.1653405 |
| TCGA-44-2665 | YES | 1301 | 0 | -0.337636689 | 0.3895193 | 0.04779416 | 0.3698974 | 1.728773 | 0.2564265 |
| TCGA-49-4488 | NOT | 869 | 1 | -0.331593837 | 0.4342372 | 0.1283186 | 0.1219159 | 2.069248 | 0 |
| TCGA-49-AAR0 | NOT | 4765 | 0 | -0.327248186 | 0.109822 | 0.004772466 | 0.3468768 | 1.126611 | 0.2194745 |
| TCGA-78-8662 | YES | 3361 | 1 | -0.326527095 | 0 | 0.1367453 | 0.05521692 | 1.614037 | 0.06288594 |
| TCGA-67-3771 | YES | 610 | 0 | -0.317359729 | 0.286727217 | 0.014240144 | 6.871351704 | 2.819402889 | 0.523896643 |
| TCGA-93-7347 | YES | 683 | 0 | -0.306063004 | 0.198036037 | 0.019124285 | 0.185334539 | 1.339807695 | 0.56286786 |
| TCGA-86-8073 | YES | 740 | 0 | -0.305003899 | 0.114857483 | 0.128347456 | 0.1382025 | 1.933010356 | 0.839452225 |
| TCGA-97-A4M3 | NOT | 540 | 0 | -0.30078787 | 0.1912719 | 0.004749706 | 0.07671612 | 1.157407 | 0.3931701 |
| TCGA-55-7281 | NOT | 872 | 0 | -0.293974664 | 0.201464813 | 0.010005635 | 0.50502697 | 1.180988792 | 0.138040705 |
| TCGA-MP-A4SY | NOT | 1501 | 1 | -0.289554502 | 0.503096 | 0.07870582 | 0.3178094 | 2.131001 | 0.9249823 |
| TCGA-44-A47G | YES | 351 | 0 | -0.271696787 | 0.1609411 | 0.0167854 | 0.8585268 | 1.320813 | 0.514614 |
| TCGA-NJ-A7XG | NOT | 617 | 0 | -0.270385554 | 0.2530252 | 0.08356624 | 0.7814296 | 1.808591 | 0.6067919 |
| TCGA-86-8076 | NOT | 993 | 0 | -0.263935724 | 0.1308737 | 0.02274919 | 0.1990297 | 1.068282 | 0.2441091 |
| TCGA-05-5428 | NOT | 670 | 0 | -0.258530765 | 0.279116 | 0.103966 | 0 | 1.636181 | 0.2549949 |
| TCGA-49-AAR4 | NOT | 879 | 1 | -0.257932066 | 0.4668613 | 0.0811524 | 0.5461476 | 2.008418 | 0.7464017 |
| TCGA-MP-A4SV | NOT | 2620 | 1 | -0.257447099 | 0.276698 | 0.01923887 | 0.6603259 | 1.611542 | 1.150176 |
| TCGA-97-A4M6 | YES | 568 | 0 | -0.256293374 | 0.02866544 | 0.00996557 | 1.247452 | 1.441867 | 1.283221 |
| TCGA-86-8074 | NOT | 24 | 0 | -0.255835426 | 0.1723561 | 0.01361813 | 0.4179178 | 1.223683 | 0.6262658 |
| TCGA-86-8585 | NOT | 353 | 0 | -0.245469166 | 3.882411 | 0.04131808 | 1.2513 | 5.663463 | 0.316687 |
| TCGA-44-2659 | YES | 1367 | 0 | -0.237044672 | 0.362781044 | 0.00315303 | 0.025463493 | 1.056453009 | 0.058000179 |
| TCGA-44-6776 | NOT | 2616 | 0 | -0.235324885 | 0.2775235 | 0.01378305 | 0.3617583 | 1.41693 | 1.01416 |
| TCGA-55-8510 | YES | 539 | 0 | -0.234771509 | 0.278648449 | 0 | 0.073343443 | 1.198734233 | 0.779613638 |
| TCGA-55-7726 | YES | 652 | 0 | -0.224796805 | 0 | 0.008540366 | 0.1724274 | 0.6503482 | 0 |
| TCGA-55-8085 | YES | 904 | 0 | -0.224744696 | 0.03930406 | 0.003416024 | 0.275874 | 0.9624807 | 0.7854746 |
| TCGA-55-7816 | YES | 468 | 1 | -0.212242593 | 0.022131727 | 0.003847059 | 0.062136773 | 0.644497003 | 0.14153376 |
| TCGA-55-7724 | NOT | 705 | 0 | -0.212150131 | 0 | 0 | 0.3190388 | 0.6016627 | 0 |
| TCGA-50-7109 | YES | 308 | 1 | -0.209195475 | 0.3703404 | 0.005364551 | 0.1083087 | 1.021275 | 0.04934062 |
| TCGA-69-8253 | YES | 426 | 0 | -0.208226505 | 0.1352748 | 0.02090153 | 0.3164967 | 0.8953029 | 0.09612122 |
| TCGA-55-8207 | YES | 977 | 0 | -0.196036564 | 0.05692441 | 0.03628135 | 0.2264121 | 0.8288466 | 0.06067252 |
| TCGA-86-8671 | YES | 839 | 0 | -0.18320362 | 0.096540572 | 0 | 0.587266677 | 0.766732788 | 0.205794306 |
| TCGA-93-A4JQ | YES | 526 | 0 | -0.175211024 | 0.4740771 | 0.03036036 | 0.8406399 | 1.71744 | 1.116961 |
| TCGA-J2-8194 | YES | 724 | 0 | -0.173169439 | 0.499483 | 0.0400721 | 0.06742032 | 1.373169 | 0.3685644 |
| TCGA-J2-A4AE | NOT | 1079 | 0 | -0.16725532 | 0.03311794 | 0.01151348 | 0.7206068 | 0.8767508 | 0.5824259 |
| TCGA-86-8075 | NOT | 694 | 1 | -0.164579894 | 0.05288313 | 0.003064146 | 0.8537258 | 0.8866708 | 0.6200166 |
| TCGA-44-6774 | NOT | 658 | 0 | -0.163009428 | 0.250985226 | 0 | 0.070466313 | 0.797337732 | 0.240759902 |
| TCGA-97-8174 | NOT | 164 | 1 | -0.161891679 | 0.01451092 | 0.007567101 | 0.2138885 | 0.5762341 | 0.1855964 |
| TCGA-J2-A4AG | YES | 988 | 0 | -0.161700108 | 0.069933068 | 0.012156151 | 0.515401023 | 0.740551871 | 0.223613142 |
| TCGA-44-3919 | YES | 1026 | 1 | -0.159875474 | 0.112353982 | 0.032549979 | 0.828039712 | 1.041044817 | 0.449069156 |
| TCGA-50-8457 | NOT | 1125 | 0 | -0.156038683 | 0.07832966 | 0.03630852 | 0.3298761 | 0.8985894 | 0.4174359 |
| TCGA-67-3774 | NOT | 385 | 0 | -0.154472772 | 0.1657824 | 0.005763443 | 0.5585386 | 0.7461057 | 0.05300945 |
| TCGA-49-4487 | YES | 855 | 1 | -0.140308542 | 0 | 0.017104309 | 0.03453309 | 0.455872087 | 0 |
| TCGA-64-5779 | YES | 864 | 0 | -0.137379422 | 0.4956429 | 0.02349692 | 0.4427694 | 1.312145 | 0.5042651 |
| TCGA-55-6980 | YES | 2109 | 0 | -0.135844066 | 0.397690197 | 0 | 0.558275127 | 1.052828903 | 0.397383337 |
| TCGA-55-7815 | NOT | 773 | 0 | -0.134255021 | 0.2211382 | 0.01921973 | 0.2716284 | 0.8781485 | 0.3535481 |
| TCGA-49-4512 | NOT | 905 | 1 | -0.123656253 | 0.234861728 | 0 | 0.274747911 | 0.725390653 | 0.250325875 |
| TCGA-55-7283 | YES | 609 | 0 | -0.119961753 | 0.02819177 | 0.02450224 | 0.4749052 | 0.8209712 | 0.7211524 |
| TCGA-78-7537 | NOT | 1622 | 1 | -0.119081709 | 0.03657284 | 0.03178646 | 0.01283518 | 0.6535448 | 0.380064 |
| TCGA-49-4505 | NOT | 428 | 1 | -0.115973323 | 0.05912748 | 0.01284733 | 0.5498936 | 0.5869938 | 0.1181637 |
| TCGA-97-8175 | NOT | 551 | 0 | -0.11351704 | 0.2721837 | 0.06420977 | 0.8597021 | 1.338198 | 0.5905714 |
| TCGA-50-5055 | YES | 1830 | 1 | -0.112775899 | 0 | 0.008457524 | 0.4439627 | 0.4508278 | 0.07778834 |
| TCGA-99-AA5R | YES | 658 | 0 | -0.110365022 | 0.092935717 | 0.010769732 | 0.217437676 | 0.697097039 | 0.594329647 |
| TCGA-78-7158 | NOT | 179 | 1 | -0.104893173 | 0.1417589 | 0.02053442 | 0.06633341 | 0.8443949 | 0.8310102 |
| TCGA-95-7567 | NOT | 568 | 0 | -0.1028407 | 0.5048021 | 0.09682481 | 1.74716 | 1.8433 | 0.3061263 |
| TCGA-78-7162 | YES | 3169 | 1 | -0.102538839 | 0.02060802 | 0.0035822 | 0.07232355 | 0.4091763 | 0.2635792 |
| TCGA-50-5935 | YES | 653 | 1 | -0.097652082 | 0.07643275 | 0.04650086 | 0.5364792 | 0.8093798 | 0.3054951 |
| TCGA-05-4430 | YES | 761 | 0 | -0.097019448 | 0.1084397 | 0.05026554 | 0.6723357 | 0.837313 | 0.1444746 |
| TCGA-67-3772 | YES | 573 | 0 | -0.095640456 | 0.2493498 | 0.03034034 | 5.355547 | 1.947349 | 0.3986518 |
| TCGA-38-7271 | YES | 800 | 1 | -0.092988649 | 0 | 0.052989841 | 0 | 0.576453126 | 0.1392502 |
| TCGA-55-8614 | YES | 536 | 0 | -0.090900517 | 0.03653096 | 0.02540005 | 0.05128194 | 0.4835529 | 0.1752133 |
| TCGA-97-A4M0 | NOT | 652 | 0 | -0.081765063 | 0.1821838 | 0 | 0.2009452 | 0.6890085 | 0.665758 |
| TCGA-97-7938 | YES | 18 | 1 | -0.081753828 | 0.245250759 | 0.018270359 | 0.245915395 | 0.834771749 | 0.560140622 |
| TCGA-55-8091 | YES | 600 | 0 | -0.081569751 | 0.02103402 | 0.003656249 | 1.122042 | 0.6125307 | 0.3699131 |
| TCGA-44-6777 | YES | 987 | 1 | -0.079844427 | 0.140757679 | 0.004893455 | 0.671822289 | 0.670745111 | 0.405069321 |
| TCGA-62-8397 | NOT | 1289 | 0 | -0.079247308 | 0 | 0 | 0.04811619 | 0.3629616 | 0.4383919 |
| TCGA-91-6835 | YES | 79 | 0 | -0.077530038 | 0.115586697 | 0.035160853 | 0.892429536 | 1.223998136 | 1.47836812 |
| TCGA-86-7714 | NOT | 625 | 1 | -0.074064134 | 0.05458518 | 0 | 0.1532527 | 0.4335198 | 0.4363445 |
| TCGA-44-A4SU | YES | 409 | 1 | -0.072982009 | 0.440703862 | 0.009575696 | 0.483326136 | 1.057323266 | 0.616509337 |
| TCGA-95-A4VP | YES | 605 | 0 | -0.071575597 | 0.5848308 | 0 | 0.3713966 | 0.9584451 | 0.08904832 |
| TCGA-73-4659 | YES | 711 | 1 | -0.068613791 | 0.2866953 | 0 | 0.1341537 | 0.5692391 | 0.1145896 |
| TCGA-50-5944 | NOT | 1750 | 0 | -0.068065639 | 0 | 0 | 0 | 0.36302 | 0.548078 |
| TCGA-38-4627 | YES | 1147 | 1 | -0.066331899 | 0.057552931 | 0.020008334 | 0.511685588 | 0.482483769 | 0.092013636 |
| TCGA-97-8552 | NOT | 626 | 0 | -0.065571455 | 0.06696922 | 0 | 1.034121 | 0.5318748 | 0.2141361 |
| TCGA-49-AARO | NOT | 3759 | 0 | -0.058387374 | 0.54041911 | 0.02935582 | 0.568977843 | 1.385974776 | 0.972003815 |
| TCGA-55-6986 | YES | 3261 | 0 | -0.057466046 | 0.1616668 | 0.05620364 | 0.0851051 | 0.7489829 | 0.2584673 |
| TCGA-97-A4M1 | NOT | 601 | 0 | -0.056276223 | 0 | 0 | 0.406380398 | 0.364941267 | 0.396704674 |
| TCGA-97-7553 | YES | 1870 | 0 | -0.055743901 | 0.074207662 | 0.008599456 | 0.069448182 | 0.425651102 | 0.395468815 |
| TCGA-73-7499 | NOT | 1531 | 1 | -0.054085471 | 0.064657838 | 0.02997115 | 2.874265762 | 1.454966575 | 1.37830288 |
| TCGA-86-7953 | YES | 997 | 0 | -0.050768663 | 0.231211435 | 0 | 0.013523885 | 0.739620566 | 0.985740934 |
| TCGA-78-7152 | YES | 1215 | 1 | -0.050348691 | 0.7514031 | 0.02798851 | 0.1883599 | 1.420882 | 0.6864671 |
| TCGA-49-AAR2 | YES | 2224 | 0 | -0.048850079 | 0.5707546 | 0.005221669 | 0.6536284 | 1.192889 | 0.7203969 |
| TCGA-69-7763 | NOT | 690 | 0 | -0.048244747 | 0.3860801 | 0.02237019 | 0.7678006 | 0.9369184 | 0.2571881 |
| TCGA-55-8616 | YES | 48 | 0 | -0.044825006 | 0.618000031 | 0.026856042 | 0.173508952 | 1.022542641 | 0.049401854 |
| TCGA-49-6744 | NOT | 1683 | 0 | -0.04466615 | 0.060016798 | 0.057378475 | 0.505507335 | 0.754708673 | 0.431787516 |
| TCGA-50-8460 | NOT | 829 | 0 | -0.044232914 | 0 | 0.02660394 | 0.1289102 | 0.4051776 | 0.3425668 |
| TCGA-91-A4BD | NOT | 603 | 0 | -0.036991586 | 0.08363556 | 0 | 1.262126 | 0.4981802 | 0.1337136 |
| TCGA-55-8097 | NOT | 476 | 0 | -0.036937195 | 0.214676195 | 0.009329049 | 0.188350727 | 0.639365687 | 0.60062954 |
| TCGA-55-8512 | YES | 607 | 1 | -0.03606041 | 0.09093264 | 0 | 0.7871793 | 0.5617066 | 0.5815199 |
| TCGA-L4-A4E6 | YES | 435 | 0 | -0.031301925 | 0 | 0.02006176 | 0.06075614 | 0.5728879 | 1.060982 |
| TCGA-78-8648 | NOT | 1209 | 1 | -0.027238706 | 0 | 0.007182954 | 0.087013019 | 0.218792532 | 0.264261762 |
| TCGA-MP-A4T9 | NOT | 1265 | 1 | -0.02321649 | 0.307942931 | 0.044606946 | 0.540360563 | 0.985076385 | 0.615410641 |
| TCGA-44-A47B | NOT | 287 | 0 | -0.019813027 | 0.3315092 | 0.03989406 | 0.7338537 | 0.9451335 | 0.4076965 |
| TCGA-44-7672 | NOT | 719 | 0 | -0.016821562 | 0.335755232 | 0.003890855 | 1.194039022 | 1.096266572 | 1.145160232 |
| TCGA-55-1596 | YES | 2065 | 0 | -0.010762856 | 1.310165 | 0.03693083 | 7.3071 | 3.843449 | 1.698362 |
| TCGA-86-A456 | NOT | 896 | 0 | -0.010176198 | 0.379341683 | 0.053950303 | 0.314669487 | 0.958605891 | 0.385940781 |
| TCGA-44-2661 | YES | 1159 | 0 | -0.00851062 | 0.07385027 | 0.03851118 | 0.7084155 | 0.5213552 | 0.1574257 |
| TCGA-64-1681 | NOT | 1167 | 1 | -0.0072729 | 0.123154791 | 0 | 0.691535844 | 0.326035 | 0.065631874 |
| TCGA-49-AAR3 | NOT | 1893 | 0 | -0.006111934 | 0.46514648 | 0.071870508 | 0.47158934 | 1.505056582 | 1.322062422 |
| TCGA-44-2657 | YES | 1351 | 0 | 0.002610392 | 0.1153804 | 0.00668535 | 0.3779306 | 0.3054533 | 0.1844661 |
| TCGA-55-7573 | YES | 487 | 0 | 0.0122189 | 0.038960955 | 0.006772408 | 0.300812362 | 0.30943096 | 0.560604856 |
| TCGA-93-A4JP | NOT | 578 | 0 | 0.013087288 | 0.04804691 | 0.004175889 | 0.168619913 | 0.25439488 | 0.499302297 |
| TCGA-55-8094 | YES | 541 | 0 | 0.013966851 | 0.1505481 | 0 | 0.1056693 | 0.4782659 | 0.9146262 |
| TCGA-MP-A4TI | NOT | 429 | 1 | 0.014317788 | 0.208707774 | 0.051826763 | 0.669674808 | 1.144515032 | 1.477702571 |
| TCGA-78-7145 | YES | 826 | 1 | 0.014511952 | 0.433626793 | 0.005383957 | 0 | 0.696979352 | 0.594229309 |
| TCGA-44-8117 | YES | 385 | 0 | 0.014755268 | 0.263274 | 0.04862397 | 1.074099 | 0.7841035 | 0.07892126 |
| TCGA-78-7163 | NOT | 7248 | 0 | 0.01991066 | 0.350038766 | 0.007158314 | 1.531956686 | 0.708636452 | 0 |
| TCGA-05-4422 | NOT | 365 | 0 | 0.022943127 | 0.03222525 | 0.0196055 | 1.379747 | 0.8957741 | 1.494098 |
| TCGA-69-7978 | YES | 134 | 0 | 0.023417805 | 0.3685828 | 0.03559393 | 4.757337 | 1.788914 | 0.6874903 |
| TCGA-99-8028 | NOT | 1118 | 0 | 0.02524156 | 0.309228466 | 0.01240426 | 0.417397189 | 0.472291675 | 0.076059043 |
| TCGA-86-8668 | NOT | 423 | 0 | 0.026817756 | 0 | 0 | 0.339302309 | 0.174511843 | 0.491816984 |
| TCGA-49-4501 | YES | 1421 | 1 | 0.028267871 | 0.1603374 | 0.03293814 | 3.877527 | 1.157648 | 0.1631267 |
| TCGA-55-6543 | NOT | 435 | 0 | 0.029934133 | 0.1960359 | 0.01135868 | 0.7109179 | 0.4324813 | 0.2089436 |
| TCGA-55-7574 | YES | 995 | 1 | 0.031905412 | 0.23549745 | 0.016374185 | 0.595061849 | 0.56110176 | 0.451806218 |
| TCGA-78-7633 | NOT | 1528 | 1 | 0.03596947 | 0.09149299 | 0.002650636 | 0.3210933 | 0.282584 | 0.5363447 |
| TCGA-86-A4P8 | YES | 805 | 0 | 0.036398249 | 0 | 0 | 0.018393311 | 0.277497715 | 1.047396852 |
| TCGA-55-8299 | NOT | 469 | 1 | 0.040893692 | 0.394147343 | 0.03425643 | 0.103744067 | 0.847802876 | 0.827070755 |
| TCGA-86-8056 | NOT | 139 | 0 | 0.042142866 | 0.1233364 | 0.05206617 | 0.5936191 | 0.839612 | 1.126777 |
| TCGA-50-5941 | YES | 1474 | 0 | 0.04379353 | 0.037827932 | 0 | 0.106205252 | 0.250360337 | 0.846691872 |
| TCGA-69-7979 | NOT | 408 | 0 | 0.046885385 | 0.109559004 | 0.00634805 | 0.205064369 | 0.410892925 | 0.934182124 |
| TCGA-55-6642 | NOT | 2449 | 0 | 0.04877707 | 0.234023431 | 0 | 0.766548292 | 0.413029428 | 0.311790476 |
| TCGA-L9-A50W | YES | 442 | 1 | 0.049928162 | 0.5598015 | 0.01556925 | 1.210203 | 1.452356 | 1.718383 |
| TCGA-44-A4SS | YES | 415 | 0 | 0.050488565 | 0.3208023 | 0.02788181 | 2.431838 | 1.741022 | 2.410572 |
| TCGA-53-7813 | NOT | 424 | 0 | 0.050815441 | 0.3827633 | 0 | 0.652461 | 0.506656 | 0.1748425 |
| TCGA-95-7043 | NOT | 503 | 1 | 0.052095561 | 0.2739223 | 0.1349083 | 0.9933705 | 1.389911 | 0.7298959 |
| TCGA-49-AARR | YES | 4992 | 0 | 0.052157287 | 0 | 0.005431278 | 1.293939528 | 0.289513972 | 0.349680456 |
| TCGA-49-4510 | YES | 896 | 1 | 0.053271353 | 0.3638654 | 0.002635379 | 0.3405281 | 0.4013677 | 0.09695591 |
| TCGA-MP-A4SW | YES | 1778 | 1 | 0.055319022 | 0.597433013 | 0.009030356 | 0.820440881 | 1.272172556 | 1.453497117 |
| TCGA-91-6847 | NOT | 842 | 0 | 0.056441414 | 0.04262055 | 0.003704269 | 0.02991523 | 0.02820798 | 0.272561 |
| TCGA-91-8497 | NOT | 434 | 1 | 0.058271392 | 0 | 0.013867045 | 0.335965806 | 0.422389718 | 1.211654457 |
| TCGA-05-4426 | YES | 791 | 0 | 0.05997396 | 0.2775039 | 0.02538803 | 0.2562884 | 0.4253252 | 0.1868058 |
| TCGA-73-4662 | YES | 2515 | 0 | 0.060367922 | 0.096498848 | 0.055114443 | 0.290281087 | 0.492686622 | 0.484876927 |
| TCGA-50-5942 | NOT | 1847 | 0 | 0.060483322 | 0.06820763 | 0 | 0.3351231 | 0.09028506 | 0.27262 |
| TCGA-05-4402 | YES | 244 | 1 | 0.061807084 | 0.5560429 | 0.02593168 | 1.237488 | 0.9873475 | 0.3035549 |
| TCGA-44-7671 | NOT | 889 | 0 | 0.063363056 | 0.1401671 | 0.02131903 | 0.2336593 | 0.2551124 | 0.252106 |
| TCGA-95-7039 | NOT | 1272 | 0 | 0.065308496 | 0.2447882 | 0.007091737 | 0.114544 | 0.7020457 | 1.500209 |
| TCGA-55-8513 | YES | 791 | 0 | 0.065700371 | 0.043100397 | 0.022475844 | 1.96638259 | 0.969869059 | 1.584870754 |
| TCGA-95-8039 | NOT | 830 | 0 | 0.067638592 | 0.2076608 | 0.01504031 | 0.6437579 | 0.6413783 | 1.02367 |
| TCGA-05-5420 | YES | 457 | 0 | 0.068133249 | 0.1888762 | 0.04103938 | 2.585147 | 1.125052 | 0.9059064 |
| TCGA-55-7570 | YES | 824 | 0 | 0.069123311 | 0.107684205 | 0.009359131 | 0.321228695 | 0.142539399 | 0.21520223 |
| TCGA-86-7954 | NOT | 605 | 0 | 0.071449812 | 0.350540001 | 0 | 1.909762191 | 1.148959049 | 1.521170498 |
| TCGA-55-7725 | YES | 442 | 0 | 0.071933626 | 0 | 0.00944792 | 0.03815014 | 0 | 0.3475901 |
| TCGA-L9-A443 | YES | 193 | 1 | 0.071953154 | 0.5864033 | 0.0283144 | 0.2743965 | 0.7762102 | 0.2083381 |
| TCGA-38-4628 | NOT | 1492 | 1 | 0.072342539 | 0.06542996 | 0.01990344 | 0.5740636 | 0.1948687 | 0.2092143 |
| TCGA-38-4630 | NOT | 1073 | 1 | 0.076075833 | 0.054271866 | 0.009433834 | 0 | 0.035919284 | 0.325379936 |
| TCGA-86-8279 | NOT | 949 | 0 | 0.077567333 | 0.3547624 | 0.03391671 | 0.04980134 | 0.9626632 | 1.588109 |
| TCGA-78-7539 | NOT | 791 | 0 | 0.077828614 | 0.2874873 | 0.03747947 | 0.4287962 | 0.4994602 | 0.2010858 |
| TCGA-49-4490 | YES | 385 | 1 | 0.081931631 | 0.2367144 | 0.0164588 | 0.2990684 | 0.3760008 | 0.4541409 |
| TCGA-73-4658 | YES | 1600 | 1 | 0.082663301 | 0.098708724 | 0 | 0.646644682 | 0.130658736 | 0.263020158 |
| TCGA-NJ-A55A | YES | 15 | 0 | 0.088217327 | 0 | 0.009858175 | 1.034974755 | 0.300279663 | 0.725366922 |
| TCGA-55-7914 | NOT | 187 | 1 | 0.089643405 | 0.215096493 | 0.026706612 | 0.107839705 | 0.447415362 | 0.736904651 |
| TCGA-91-6840 | YES | 372 | 0 | 0.092732391 | 0 | 0 | 0.3184728 | 0.06005953 | 0.6528693 |
| TCGA-86-8054 | YES | 1148 | 0 | 0.09382005 | 0.361767 | 0 | 0.04232052 | 0.1862247 | 0 |
| TCGA-78-7153 | YES | 3635 | 0 | 0.095027952 | 0.02923434 | 0.0203267 | 0.205195 | 0.1934845 | 0.7010828 |
| TCGA-05-4415 | NOT | 91 | 1 | 0.096655117 | 0.160375977 | 0.013938717 | 0.187612367 | 0.070762143 | 0.042733928 |
| TCGA-93-7348 | YES | 531 | 0 | 0.09744427 | 0.068707358 | 0.035829273 | 0.241127448 | 0.27283961 | 0.549234744 |
| TCGA-44-3918 | YES | 1036 | 0 | 0.098546139 | 1.119298 | 0.007483173 | 1.208664 | 1.481592 | 0.4473737 |
| TCGA-55-A48Z | YES | 651 | 0 | 0.09968037 | 0.230305392 | 0.030024713 | 0.646602673 | 0.457275868 | 0.414229837 |
| TCGA-78-7535 | NOT | 949 | 1 | 0.102512175 | 0.638362 | 0.03698787 | 2.11586 | 1.126649 | 0.02834975 |
| TCGA-44-6775 | YES | 705 | 0 | 0.107236599 | 0.156507906 | 0.010882026 | 0.263645838 | 0.372899415 | 1.000877717 |
| TCGA-86-7711 | YES | 1046 | 1 | 0.108522312 | 0.477196156 | 0.008887378 | 0.131584577 | 0.40606385 | 0.163483868 |
| TCGA-97-7554 | NOT | 775 | 0 | 0.108550596 | 0.33970964 | 0.027253938 | 0.38517422 | 0.345897511 | 0 |
| TCGA-50-6590 | YES | 1288 | 1 | 0.111882071 | 0.1370212 | 0.05359 | 0.312568 | 0.4080874 | 0.4928957 |
| TCGA-55-8206 | YES | 888 | 0 | 0.112282631 | 0 | 0.02207982 | 0.178314 | 0.04803932 | 0.4931938 |
| TCGA-93-A4JO | YES | 33 | 1 | 0.119243391 | 0.1527629 | 0.01062164 | 0.3216715 | 0.3235346 | 0.9280817 |
| TCGA-55-6968 | NOT | 1293 | 1 | 0.121811463 | 0.117024146 | 0.006780594 | 0.273796331 | 0.051634163 | 0.374188319 |
| TCGA-MP-A4T6 | YES | 1790 | 1 | 0.122595727 | 0.3673157 | 0.05586773 | 1.530793 | 1.033193 | 0.8441742 |
| TCGA-55-6983 | NOT | 2823 | 0 | 0.124500998 | 0.2815167 | 0.009786956 | 0.4478837 | 0.3229529 | 0.4800844 |
| TCGA-55-8619 | NOT | 416 | 0 | 0.128285641 | 0.03206176 | 0.011146303 | 0.157528378 | 0.254637013 | 1.281480855 |
| TCGA-L9-A8F4 | NOT | 476 | 0 | 0.131007971 | 0.96130302 | 0 | 0.195318236 | 0.93760013 | 0.364002167 |
| TCGA-05-4249 | NOT | 1523 | 0 | 0.131634796 | 0.098079001 | 0.051145895 | 0.068841359 | 0.493335706 | 1.191720409 |
| TCGA-50-6597 | NOT | 1268 | 1 | 0.131767519 | 0.1884125 | 0.008187725 | 0.2644924 | 0.06234945 | 0.2259206 |
| TCGA-05-4427 | NOT | 791 | 0 | 0.131846035 | 0.296157781 | 0.03431985 | 0.323356855 | 0.392018051 | 0.368267529 |
| TCGA-55-7227 | NOT | 952 | 1 | 0.132490069 | 0.4083494 | 0.0414059 | 0.2627343 | 0.7206985 | 0.8704733 |
| TCGA-55-A4DF | NOT | 440 | 1 | 0.137661671 | 0.144744048 | 0.025160207 | 0.833082411 | 0.421508477 | 0.833082411 |
| TCGA-55-A491 | YES | 626 | 0 | 0.13876006 | 0.150888185 | 0 | 0.953171705 | 0.239673158 | 0.627210517 |
| TCGA-44-6145 | NOT | 595 | 0 | 0.140755871 | 0.499654093 | 0 | 0.809320888 | 0.814008607 | 1.228968756 |
| TCGA-55-8087 | NOT | 462 | 0 | 0.143999157 | 0.1046659 | 0.07580669 | 0 | 0.4387231 | 0.7530131 |
| TCGA-44-6148 | YES | 704 | 0 | 0.146301027 | 0.065192511 | 0.022664242 | 0.823651728 | 0.129441008 | 0.364796367 |
| TCGA-55-6985 | NOT | 1233 | 0 | 0.150336164 | 0 | 0.021771019 | 0.586067169 | 0.165786122 | 0.867705003 |
| TCGA-44-7669 | YES | 574 | 1 | 0.150807063 | 0.0781439 | 0.01018755 | 0.3290938 | 0.05171875 | 0.6246689 |
| TCGA-MP-A4T7 | YES | 167 | 1 | 0.151020201 | 0.3484574 | 0.1124886 | 0.8385642 | 1.120169 | 0.8754455 |
| TCGA-86-8669 | YES | 938 | 0 | 0.159953583 | 0.4172489 | 0 | 0.5058591 | 0.6025134 | 1.212878 |
| TCGA-S2-AA1A | YES | 513 | 0 | 0.161591704 | 0 | 0.01331485 | 1.129056 | 0.4562661 | 1.592029 |
| TCGA-44-2666 | YES | 97 | 1 | 0.162549965 | 0.2488404 | 0.007209135 | 0.1455503 | 0.1646925 | 0.6299093 |
| TCGA-95-7562 | NOT | 87 | 1 | 0.162870987 | 0.3079502 | 0.0033456 | 0.3782613 | 0.178337 | 0.4000263 |
| TCGA-NJ-A4YI | YES | 4 | 1 | 0.169224608 | 0.1585792 | 0.06891277 | 0.2226125 | 0.3148621 | 0.3802964 |
| TCGA-97-A4LX | YES | 614 | 0 | 0.169301765 | 0.243831819 | 0.004709354 | 0.703595398 | 0.25103185 | 0.649716471 |
| TCGA-69-7980 | NOT | 411 | 0 | 0.170965778 | 0.186663907 | 0.058404506 | 0.786113037 | 0.518874781 | 0.716236323 |
| TCGA-NJ-A4YG | NOT | 2261 | 0 | 0.171567041 | 0.3119116 | 0.04337456 | 1.46683 | 0.6605938 | 0.5485409 |
| TCGA-62-8395 | YES | 1216 | 0 | 0.172066331 | 0.651013495 | 0.019680478 | 0.377475835 | 0.861733365 | 1.040817871 |
| TCGA-95-7944 | NOT | 377 | 0 | 0.172829383 | 0.118441 | 0.01372538 | 0.2771112 | 0.3396855 | 1.451755 |
| TCGA-69-7764 | YES | 414 | 0 | 0.173015375 | 0.4223635 | 0.02002297 | 0.2695052 | 0.4065993 | 0.5524856 |
| TCGA-64-1679 | NOT | 2488 | 0 | 0.178827628 | 0.5730775 | 0.01383548 | 0.1340805 | 0.4424998 | 0.3817569 |
| TCGA-62-A46R | NOT | 1725 | 1 | 0.179230499 | 0.273897959 | 0.0105801 | 0.170887254 | 0.040283686 | 0.243276994 |
| TCGA-55-A4DG | YES | 608 | 0 | 0.188157039 | 0.4228746 | 0.09612374 | 0 | 0.9042124 | 1.040119 |
| TCGA-55-6971 | NOT | 1400 | 0 | 0.188170564 | 0.449916113 | 0 | 0.140353235 | 0.264686594 | 0.559463589 |
| TCGA-MP-A4T4 | YES | 2617 | 1 | 0.188485093 | 0.385523918 | 0.020619658 | 0.478750465 | 0.510310195 | 0.948249713 |
| TCGA-55-8204 | YES | 515 | 0 | 0.193266456 | 0.3741917 | 0.008130508 | 0.4760424 | 0.1238275 | 0.1121709 |
| TCGA-86-8055 | NOT | 124 | 1 | 0.195583736 | 0.228569191 | 0.066218591 | 1.457257641 | 0.756381011 | 0.913571304 |
| TCGA-64-5775 | NOT | 62 | 1 | 0.20012085 | 0.217645 | 0.05201934 | 0.01909554 | 0.03601153 | 0 |
| TCGA-78-7143 | YES | 4961 | 1 | 0.200562883 | 0.243993806 | 0 | 1.370068202 | 0.466511724 | 1.170266589 |
| TCGA-50-5931 | YES | 434 | 1 | 0.202070267 | 0.3056783 | 0.03320917 | 0 | 0.1517325 | 0.3665309 |
| TCGA-NJ-A4YF | NOT | 2161 | 0 | 0.203964673 | 1.83002 | 0 | 0 | 1.599672 | 0.05520327 |
| TCGA-44-7660 | YES | 592 | 0 | 0.20663032 | 0.37240094 | 0.004045799 | 0.114356861 | 0.184852341 | 0.669804469 |
| TCGA-05-4432 | NOT | 761 | 0 | 0.207199296 | 0.4820909 | 0.01496423 | 0.4592275 | 0.3418575 | 0.3853751 |
| TCGA-55-8090 | NOT | 598 | 1 | 0.208536495 | 0.456639682 | 0.028348437 | 0.938648235 | 0.539682759 | 0.547544804 |
| TCGA-NJ-A55R | NOT | 603 | 0 | 0.21010382 | 0.07939374 | 0.1909091 | 3.807959 | 2.014261 | 1.734737 |
| TCGA-44-A479 | NOT | 486 | 0 | 0.214478424 | 0.1428166 | 0.01241258 | 1.628941 | 0.1890434 | 0.4566605 |
| TCGA-86-8358 | YES | 653 | 0 | 0.214539626 | 0 | 0.061988232 | 0.250304785 | 0.177014947 | 0.926475351 |
| TCGA-78-7160 | NOT | 697 | 1 | 0.216616044 | 0.522789 | 0.02423308 | 0.2935552 | 0.8304062 | 1.671634 |
| TCGA-L9-A743 | NOT | 664 | 0 | 0.217786331 | 0.198164749 | 0.059050435 | 0.337793027 | 0.674502842 | 1.719874301 |
| TCGA-05-4398 | NOT | 1431 | 0 | 0.218242539 | 0.8526284 | 0.008718151 | 0.01760169 | 0.9460382 | 1.343106 |
| TCGA-97-8547 | YES | 657 | 0 | 0.220251406 | 0.2958281 | 0.08815279 | 1.868767 | 0.8950436 | 0.6756567 |
| TCGA-44-3396 | NOT | 1130 | 0 | 0.222313558 | 0.57929485 | 0.059938226 | 0.435648566 | 0.693772197 | 0.485129638 |
| TCGA-NJ-A55O | YES | 13 | 0 | 0.223094976 | 0.3788182 | 0.01097471 | 1.130038 | 0.8357233 | 1.917865 |
| TCGA-97-7547 | YES | 1965 | 0 | 0.224546818 | 0.1808285 | 0.02750353 | 0.07932687 | 0.1495994 | 0.9757205 |
| TCGA-86-6851 | YES | 179 | 0 | 0.225134338 | 0.1965827 | 0.02174524 | 0.2257868 | 0.02365568 | 0.5714356 |
| TCGA-64-5778 | NOT | 1305 | 0 | 0.225904056 | 0.4219602 | 0.1152603 | 0.1480863 | 0.5984359 | 0.04818682 |
| TCGA-44-7667 | NOT | 1097 | 0 | 0.231745288 | 0.5414496 | 0.003619913 | 0 | 0.05513122 | 0 |
| TCGA-50-5066 | NOT | 1442 | 0 | 0.236938579 | 0.5161353 | 0.04013677 | 0.5910784 | 1.042776 | 2.019518 |
| TCGA-44-8120 | NOT | 260 | 0 | 0.239550027 | 0.4654736 | 0.04045559 | 0.08796161 | 0.4502547 | 0.801428 |
| TCGA-49-4514 | YES | 1700 | 0 | 0.241421374 | 0.5665559 | 0 | 0.8218387 | 0.3999672 | 0.543474 |
| TCGA-05-4417 | NOT | 455 | 0 | 0.243132117 | 0.37295097 | 0.012965678 | 0.026177317 | 0.098733528 | 0.596261119 |
| TCGA-55-6978 | NOT | 176 | 1 | 0.243242774 | 0.123644471 | 0.060895594 | 0.593035519 | 0.436441813 | 1.284910291 |
| TCGA-MP-A4TJ | NOT | 339 | 1 | 0.245470861 | 0.270763 | 0 | 0.4434448 | 0.05973393 | 0.7936254 |
| TCGA-35-4123 | NOT | 182 | 0 | 0.249677672 | 0.218279 | 0.008431657 | 2.740745 | 0.385242 | 0.4265273 |
| TCGA-55-A57B | NOT | 546 | 0 | 0.25086568 | 0.04282676 | 0.1042214 | 2.164318 | 0.7653004 | 0.9243443 |
| TCGA-50-6595 | NOT | 189 | 1 | 0.252062932 | 0.0755461 | 0.0328296 | 1.033999 | 0.09999884 | 0.724683 |
| TCGA-50-8459 | NOT | 1119 | 0 | 0.252349845 | 0.05636391 | 0.02939246 | 2.413261 | 0.1865194 | 0.270338 |
| TCGA-64-5815 | NOT | 866 | 0 | 0.258278629 | 0.252739108 | 0.073220838 | 3.222709014 | 0.947879295 | 0.673450915 |
| TCGA-05-4420 | NOT | 912 | 0 | 0.258803618 | 0.6280307 | 0.01015514 | 0.3177953 | 0.2126611 | 0.07005166 |
| TCGA-91-6831 | YES | 310 | 0 | 0.259078834 | 0.7526478 | 0.0218049 | 0.05869791 | 0.3874362 | 0.1337008 |
| TCGA-50-5044 | YES | 624 | 1 | 0.260781263 | 0 | 0.079345759 | 2.306832807 | 0.362530369 | 0.291914028 |
| TCGA-99-8032 | NOT | 44 | 0 | 0.263993189 | 0.4473817 | 0.01110948 | 0.3439221 | 0.1691972 | 0.5449588 |
| TCGA-99-8033 | YES | 656 | 1 | 0.264376031 | 1.809178 | 0.01797036 | 1.741518 | 2.75969 | 2.561886 |
| TCGA-99-7458 | NOT | 747 | 0 | 0.264783326 | 0.3512425 | 0.04070327 | 0.123268 | 0.1808071 | 0.5615544 |
| TCGA-55-6982 | YES | 995 | 1 | 0.265123974 | 0.559541753 | 0 | 4.287419183 | 1.851635188 | 2.83282404 |
| TCGA-55-8621 | NOT | 515 | 0 | 0.267216923 | 0.4855472 | 0.03692521 | 0.5112062 | 0.3615238 | 0.4851725 |
| TCGA-78-7147 | YES | 586 | 1 | 0.26993723 | 0.368920734 | 0 | 1.375641932 | 0.091562418 | 0.184318037 |
| TCGA-62-A46Y | YES | 414 | 1 | 0.273028582 | 0.3008585 | 0.07131392 | 0.5375279 | 0.2534256 | 0.2623648 |
| TCGA-86-7713 | YES | 1157 | 0 | 0.274186262 | 0.6099935 | 0 | 0.1352061 | 0.1062416 | 0.2053129 |
| TCGA-55-7910 | YES | 1040 | 0 | 0.279250324 | 0.7034291 | 0 | 0.8111358 | 0.4655574 | 0.5623091 |
| TCGA-62-A470 | NOT | 1194 | 1 | 0.280492618 | 0.283371 | 0.01231428 | 0.3977946 | 0.2813194 | 1.472392 |
| TCGA-38-4632 | NOT | 1357 | 1 | 0.280859506 | 0.7842757 | 0.03029492 | 1.743189 | 0.8651083 | 0.4179576 |
| TCGA-73-4668 | NOT | 467 | 0 | 0.28258576 | 0.3636416 | 0.06321019 | 0.1570701 | 0.1481062 | 0.1788854 |
| TCGA-78-7146 | YES | 173 | 1 | 0.285610871 | 1.359818 | 0.02675899 | 0.6122901 | 1.426386 | 0.9024275 |
| TCGA-35-4122 | YES | 225 | 0 | 0.289321682 | 0.422736 | 0.00408235 | 1.302258 | 0.2176094 | 0.488118 |
| TCGA-86-7955 | NOT | 1072 | 0 | 0.29664155 | 0.4764814 | 0.01911337 | 0.7975129 | 0.4366445 | 1.054775 |
| TCGA-91-A4BC | NOT | 44 | 0 | 0.297194397 | 0.2329259 | 0.08097691 | 0.5449666 | 0.3597058 | 0.8068533 |
| TCGA-91-6848 | YES | 224 | 0 | 0.29940527 | 0.141964966 | 0.098708527 | 0.033214929 | 0.250554863 | 0.832218494 |
| TCGA-97-A4M7 | YES | 629 | 0 | 0.303506422 | 0.072958376 | 0.088774185 | 1.365580437 | 0.418485266 | 0.933146632 |
| TCGA-50-5933 | NOT | 2393 | 1 | 0.306393505 | 0.5410906 | 0.033196 | 0.3127681 | 0.3791809 | 0.8141901 |
| TCGA-L4-A4E5 | YES | 578 | 0 | 0.308213568 | 0.3257585 | 0.1415628 | 0.7431089 | 0.7905331 | 0.7378161 |
| TCGA-55-6981 | YES | 1379 | 1 | 0.322976245 | 0.205016134 | 0 | 4.317006398 | 0.746283176 | 1.229147655 |
| TCGA-93-A4JN | NOT | 718 | 0 | 0.325325188 | 0.08028062 | 0.055819277 | 0.225394912 | 0 | 1.069582336 |
| TCGA-49-6745 | NOT | 522 | 0 | 0.328167922 | 0.295083345 | 0.025646505 | 1.159861472 | 0.156238337 | 0.754830482 |
| TCGA-86-8359 | NOT | 444 | 1 | 0.335675028 | 0.546696463 | 0.012670638 | 0.358142976 | 0.28946041 | 1.048847288 |
| TCGA-44-7662 | NOT | 218 | 0 | 0.335687936 | 0.5792605 | 0.02746098 | 0.517467 | 0.2323501 | 0.449019 |
| TCGA-86-6562 | YES | 376 | 1 | 0.335881521 | 0.373185947 | 0.047177626 | 1.333503074 | 0.269442912 | 0.433917667 |
| TCGA-78-7155 | YES | 1171 | 1 | 0.336901907 | 0 | 0.1622193 | 0 | 0.04751147 | 0 |
| TCGA-NJ-A4YP | YES | 50 | 0 | 0.338248126 | 0.08607224 | 0.06412093 | 0.957991 | 0.2929682 | 1.41541 |
| TCGA-69-7973 | NOT | 230 | 0 | 0.33963535 | 0.645539 | 0 | 0.2555959 | 0.1752794 | 0.6880436 |
| TCGA-50-5946 | NOT | 1617 | 0 | 0.341115361 | 0.248877 | 0.09012744 | 0.3348149 | 0.2470751 | 0.7294759 |
| TCGA-97-A4M5 | YES | 634 | 0 | 0.341930007 | 0.02406351 | 0.07947418 | 1.55389 | 0.2229667 | 0.846382 |
| TCGA-55-8506 | NOT | 11 | 0 | 0.342084558 | 0.405941753 | 0.058802525 | 1.49587886 | 0.313446507 | 0.216335214 |
| TCGA-MN-A4N1 | YES | 827 | 0 | 0.353943079 | 0.3940501 | 0.1070249 | 0.9853255 | 0.4237969 | 0.196873 |
| TCGA-49-4494 | YES | 1081 | 1 | 0.361566705 | 0.1407125 | 0.02989484 | 3.775043 | 0.4346025 | 0.6748986 |
| TCGA-78-7536 | NOT | 244 | 1 | 0.36716432 | 0.9555309 | 0.03436462 | 0.1618893 | 0.5015653 | 0.3160696 |
| TCGA-78-7148 | YES | 626 | 1 | 0.373865728 | 0.468203 | 0.01436217 | 0.7539166 | 0.1822797 | 1.01274 |
| TCGA-86-7701 | YES | 947 | 0 | 0.375351493 | 0.5713933 | 0 | 1.024929 | 0.168076 | 0.7105186 |
| TCGA-73-4675 | NOT | 922 | 1 | 0.379641449 | 0.3026045 | 0.04429506 | 0.5589401 | 0.2740615 | 1.502307 |
| TCGA-38-4625 | YES | 2973 | 0 | 0.38060907 | 0.1848762 | 0.009181773 | 0.6580893 | 0.2272372 | 2.259028 |
| TCGA-55-1592 | NOT | 701 | 1 | 0.382387289 | 0.3656403 | 0.06066862 | 0.7932565 | 0.4179919 | 1.328575 |
| TCGA-NJ-A4YQ | YES | 1432 | 0 | 0.384421226 | 1.297322 | 0.2383938 | 0.7024525 | 2.207879 | 0.6518643 |
| TCGA-91-7771 | NOT | 492 | 0 | 0.390324551 | 0.2549124 | 0.01661636 | 1.543205 | 0.2530668 | 1.528294 |
| TCGA-55-8203 | NOT | 547 | 0 | 0.391659606 | 0.6128311 | 0.01614025 | 1.016705 | 0.2212342 | 0.5938019 |
| TCGA-55-7903 | NOT | 567 | 0 | 0.395886395 | 0.1100624 | 0.110007 | 0.328323 | 0.2185311 | 1.187757 |
| TCGA-86-8672 | NOT | 19 | 1 | 0.399350272 | 0.5961569 | 0.03700974 | 0.478218 | 0.1690973 | 0.5446372 |
| TCGA-49-4506 | YES | 999 | 1 | 0.399711392 | 0.5780745 | 0.05303327 | 0.5522692 | 0.1062758 | 0.1283619 |
| TCGA-78-7159 | NOT | 1974 | 0 | 0.401449255 | 1.014972506 | 0 | 0.29334372 | 0.276602638 | 0.19090623 |
| TCGA-69-8453 | YES | 813 | 0 | 0.402403437 | 0.026331122 | 0.036616164 | 1.626392741 | 0.069707943 | 1.47340504 |
| TCGA-55-8615 | YES | 446 | 0 | 0.40372689 | 0.7997211 | 0 | 0.92834 | 0.2442864 | 0.4425807 |
| TCGA-55-7907 | NOT | 343 | 1 | 0.411957838 | 0.9105421 | 0.03956886 | 0.6071515 | 1.08474 | 2.110828 |
| TCGA-55-6970 | YES | 464 | 1 | 0.41368615 | 0.896213 | 0.03047961 | 0.09572488 | 0.464204 | 0.8410115 |
| TCGA-MP-A4TF | YES | 336 | 1 | 0.421860927 | 0.734286806 | 0.135145878 | 3.122679599 | 1.486527914 | 0.897728277 |
| TCGA-71-8520 | NOT | 210 | 1 | 0.423093197 | 0.07628411 | 0.05304049 | 2.034656 | 0.353415 | 1.768426 |
| TCGA-78-7161 | NOT | 291 | 1 | 0.434221592 | 0.8978869 | 0.04649062 | 0.3218166 | 0.6827638 | 1.221711 |
| TCGA-78-7542 | YES | 321 | 1 | 0.43869777 | 0.4257789 | 0.0797044 | 0.5172455 | 0.1300604 | 0.5759943 |
| TCGA-MN-A4N5 | NOT | 84 | 0 | 0.439873449 | 0.9230394 | 0.144403 | 0.3077423 | 1.221809 | 1.180579 |
| TCGA-97-7941 | YES | 484 | 0 | 0.441126761 | 0.0389813 | 0.03049175 | 1.436445 | 0.2579938 | 2.461717 |
| TCGA-97-8176 | NOT | 468 | 1 | 0.441663656 | 0.839536649 | 0.03648322 | 1.153983697 | 1.713219698 | 4.082592442 |
| TCGA-62-A46P | YES | 594 | 1 | 0.441725631 | 0.5718435 | 0.06390063 | 0.08600898 | 0.1622009 | 0.7183342 |
| TCGA-91-6829 | YES | 1258 | 1 | 0.449955675 | 0.159069503 | 0.149311817 | 0.267960984 | 0.336891323 | 1.169848187 |
| TCGA-05-4396 | NOT | 303 | 1 | 0.455605399 | 0.4078323 | 0.04726111 | 0.06361251 | 0.2999108 | 2.028532 |
| TCGA-55-6712 | NOT | 171 | 1 | 0.456396581 | 0.557766114 | 0.014915996 | 1.264827973 | 0 | 0.480166175 |
| TCGA-86-A4D0 | NOT | 116 | 1 | 0.45831442 | 0.3775637 | 0.004687871 | 1.268266 | 0.1070943 | 1.552206 |
| TCGA-50-5932 | NOT | 1235 | 1 | 0.459789439 | 0.3841141 | 0 | 0.4044128 | 0.127111 | 2.187761 |
| TCGA-62-A46S | NOT | 1653 | 1 | 0.461842818 | 1.074886 | 0.02607106 | 0.1052734 | 0.4963272 | 0.759333 |
| TCGA-05-4405 | NOT | 610 | 0 | 0.466445915 | 0.2946297 | 0.139009852 | 0.265885339 | 0.501422607 | 1.480423307 |
| TCGA-50-6593 | NOT | 336 | 1 | 0.467527321 | 0.564191097 | 0.032690267 | 0.13200135 | 0.199148885 | 1.503348706 |
| TCGA-95-7947 | NOT | 477 | 0 | 0.467881266 | 0.2101744 | 0.003653367 | 0.7080997 | 0.08346107 | 2.452942 |
| TCGA-55-A48X | YES | 689 | 0 | 0.486050915 | 0.246630744 | 0.107176783 | 0.757353554 | 0.081615029 | 0.788609415 |
| TCGA-50-5936 | YES | 257 | 1 | 0.486350979 | 0.3682094 | 0.01600105 | 1.163003 | 0.2436957 | 2.060382 |
| TCGA-55-8096 | YES | 719 | 1 | 0.493331977 | 0.3791438 | 0.005991352 | 2.721681 | 0.1824964 | 1.129666 |
| TCGA-99-8025 | YES | 1060 | 0 | 0.493841846 | 0.8212918 | 0.03059174 | 0.4666599 | 0.02588398 | 0.187579 |
| TCGA-97-8171 | NOT | 568 | 0 | 0.498229472 | 0 | 0 | 0.7138299 | 0.3817535 | 4.271141 |
| TCGA-91-8499 | YES | 36 | 0 | 0.503392265 | 0 | 0.2098156 | 0.3552871 | 0.05154014 | 0.2490046 |
| TCGA-05-5715 | NOT | 62 | 0 | 0.505178758 | 0.130504073 | 0.022684936 | 0.793870308 | 0.403073058 | 3.546971824 |
| TCGA-91-6836 | NOT | 417 | 0 | 0.505806487 | 0.7377199 | 0.004932097 | 0.9360292 | 0.07511576 | 0.8165361 |
| TCGA-49-6767 | YES | 677 | 0 | 0.506269523 | 0.3297426 | 0.03821179 | 0.66862 | 0.1454912 | 1.991573 |
| TCGA-49-AARN | NOT | 1135 | 1 | 0.510939078 | 0.141209791 | 0.081819543 | 2.676097749 | 0.24922205 | 1.053552886 |
| TCGA-44-7670 | NOT | 882 | 0 | 0.515977543 | 0.151985099 | 0.029721247 | 3.760395546 | 0.125737246 | 0.880833393 |
| TCGA-55-8507 | NOT | 418 | 0 | 0.516124765 | 1.380312 | 0.02856351 | 0.3690808 | 0.6960348 | 0.5254275 |
| TCGA-50-6673 | YES | 22 | 1 | 0.523888978 | 0.2612107 | 0.05675636 | 2.520967 | 0.2160996 | 1.14844 |
| TCGA-97-8172 | NOT | 545 | 0 | 0.53577175 | 0.5459181 | 0.04744725 | 0.1915892 | 0.1487749 | 1.642908 |
| TCGA-49-AARE | YES | 1229 | 1 | 0.53987275 | 0.726482666 | 0.037884365 | 1.070823637 | 0.320543459 | 1.200191219 |
| TCGA-55-7728 | NOT | 704 | 0 | 0.545394767 | 0.2307857 | 0.0267443 | 1.700872 | 0.0763716 | 1.967852 |
| TCGA-55-8205 | YES | 599 | 0 | 0.545650457 | 0.260934853 | 0.024423071 | 0.845304902 | 0.106275131 | 2.503041736 |
| TCGA-50-5939 | YES | 460 | 1 | 0.549123499 | 0.9163813 | 0 | 0.5554955 | 0.2205446 | 1.265295 |
| TCGA-83-5908 | YES | 824 | 0 | 0.551031115 | 0.770000125 | 0.019683185 | 1.700861906 | 0.509616778 | 1.59312299 |
| TCGA-38-4629 | YES | 864 | 1 | 0.552428005 | 0.3252802 | 0.1017756 | 1.118735 | 0.06458502 | 0.8320748 |
| TCGA-91-6830 | YES | 60 | 0 | 0.55480823 | 0.3713204 | 0.01613624 | 1.531193 | 0.6758252 | 3.561925 |
| TCGA-44-8119 | NOT | 285 | 0 | 0.55793516 | 1.208126 | 0.02500036 | 0.06056998 | 0.1903775 | 0.1839533 |
| TCGA-50-6594 | NOT | 370 | 1 | 0.564100949 | 0.8627853 | 0.1349767 | 0.756983 | 0.6852308 | 0.6207261 |
| TCGA-69-7974 | NOT | 184 | 0 | 0.565289114 | 1.386948 | 0.0662989 | 0.803133 | 1.101525 | 1.164137 |
| TCGA-55-8092 | YES | 154 | 1 | 0.568411921 | 0.276975863 | 0.125918918 | 0.912224653 | 0.42303154 | 1.873466843 |
| TCGA-64-5774 | YES | 2676 | 0 | 0.571387199 | 1.325759 | 0.005237515 | 0.655612 | 0.3589527 | 0.3372055 |
| TCGA-73-A9RS | NOT | 340 | 1 | 0.581762731 | 0.731149016 | 0.127092346 | 0.63162008 | 0.297786809 | 0.269754409 |
| TCGA-55-6984 | YES | 760 | 1 | 0.582775641 | 0.041765463 | 0.014519805 | 1.201917219 | 0.165852291 | 3.605751657 |
| TCGA-95-A4VN | NOT | 553 | 0 | 0.585332375 | 1.171835129 | 0.123164317 | 0.40168975 | 1.298624152 | 1.91705902 |
| TCGA-55-1594 | YES | 1178 | 0 | 0.598888062 | 0.523095 | 0.03463896 | 3.16456 | 0.1318877 | 0.5575375 |
| TCGA-50-5930 | NOT | 282 | 1 | 0.613794282 | 1.150106227 | 0 | 1.210884199 | 0.380593074 | 1.03429692 |
| TCGA-55-8511 | NOT | 552 | 0 | 0.616790283 | 1.844444456 | 0.023748999 | 0.095897042 | 0.859030018 | 0.436864302 |
| TCGA-55-7913 | NOT | 561 | 1 | 0.618044114 | 0.5607339 | 0.01476816 | 2.087153 | 0.1574432 | 1.602801 |
| TCGA-55-A493 | NOT | 28 | 0 | 0.628634472 | 0.651690035 | 0.029551395 | 0.696072837 | 0.337550435 | 2.491498807 |
| TCGA-93-8067 | YES | 186 | 0 | 0.637987088 | 0.9225583 | 0.03805251 | 1.701167 | 0.2897696 | 0.7999752 |
| TCGA-97-7552 | YES | 1932 | 0 | 0.643067739 | 0.05191604 | 0.02256083 | 2.259262 | 0.1030803 | 3.071058 |
| TCGA-05-4389 | NOT | 1369 | 0 | 0.650633704 | 0.2594352 | 0.06244124 | 6.205298 | 1.21514 | 2.648185 |
| TCGA-05-5425 | YES | 882 | 0 | 0.651807116 | 0.06583722 | 0.03433256 | 4.158985 | 0.3485896 | 2.473569 |
| TCGA-05-4434 | NOT | 457 | 1 | 0.652163152 | 1.09679 | 0.03215784 | 1.762269 | 0.4197971 | 0.7605584 |
| TCGA-53-A4EZ | NOT | 1071 | 0 | 0.653626226 | 1.47511 | 0 | 0.3559103 | 0.6406882 | 1.510821 |
| TCGA-78-7166 | NOT | 258 | 1 | 0.66218978 | 0.9726259 | 0.03719478 | 1.774976 | 0.1802423 | 0.4665002 |
| TCGA-38-A44F | YES | 133 | 0 | 0.663533701 | 0.09949621 | 0.03458996 | 6.238691 | 0.1317011 | 0.5832607 |
| TCGA-44-6778 | NOT | 1864 | 0 | 0.663762526 | 0 | 0.27751427 | 0 | 0.10566344 | 0.701922666 |
| TCGA-55-A490 | YES | 99 | 1 | 0.666453515 | 1.590496367 | 0 | 0.119610499 | 0.338353039 | 0.499484584 |
| TCGA-91-6828 | NOT | 323 | 0 | 0.666852044 | 0.634155 | 0.03674411 | 0.5563894 | 0.06995151 | 2.02773 |
| TCGA-L9-A7SV | YES | 565 | 0 | 0.669993791 | 0.06783146 | 0.2947709 | 0.07935117 | 0.05985809 | 0.07229773 |
| TCGA-55-7576 | NOT | 670 | 0 | 0.675421158 | 0.4710664 | 0.122825 | 1.849518 | 0.1948566 | 0.8472652 |
| TCGA-55-8302 | YES | 478 | 0 | 0.685229537 | 1.127336 | 0.00559885 | 3.210311 | 0.5116223 | 0.7209383 |
| TCGA-78-7540 | YES | 1197 | 1 | 0.700337393 | 0.1397012 | 0.009106361 | 6.643284 | 0.1849196 | 1.032991 |
| TCGA-49-6761 | NOT | 354 | 0 | 0.701839774 | 1.829805 | 0.02765798 | 0.2512828 | 0.6844996 | 0.4451739 |
| TCGA-50-6592 | NOT | 777 | 1 | 0.706515318 | 0.1512537 | 0.04381957 | 1.627855 | 0.1668428 | 3.425771 |
| TCGA-4B-A93V | YES | 300 | 1 | 0.719219815 | 1.925861 | 0.004523836 | 0.01826698 | 0.4478366 | 0.08321623 |
| TCGA-50-6591 | YES | 119 | 1 | 0.727135472 | 0.1442852 | 0.2946952 | 0.1265917 | 0.04774684 | 0.1730086 |
| TCGA-62-A46O | YES | 1454 | 1 | 0.738118568 | 0.266931404 | 0.017399807 | 3.957945279 | 0.088332922 | 2.080458416 |
| TCGA-62-8398 | YES | 444 | 1 | 0.739764219 | 0.68545997 | 0.023061372 | 3.057454677 | 0.878060774 | 3.429074239 |
| TCGA-55-6975 | NOT | 118 | 1 | 0.740285864 | 0.652890872 | 0.130948944 | 1.51579021 | 0.199435018 | 0.802937966 |
| TCGA-78-7154 | NOT | 593 | 1 | 0.760344987 | 0 | 0.0462784 | 7.765462 | 0.4307228 | 1.371529 |
| TCGA-MP-A4TE | YES | 896 | 1 | 0.774965644 | 1.163841 | 0 | 0.7998782 | 0.1925692 | 1.86071 |
| TCGA-05-4403 | YES | 578 | 0 | 0.776705407 | 0.1051931 | 0.02742786 | 6.598978 | 0.139242 | 1.303388 |
| TCGA-53-7624 | NOT | 1043 | 1 | 0.783675228 | 0.1093837 | 0.1045752 | 0.5758208 | 0.03619725 | 3.410139 |
| TCGA-62-8394 | NOT | 139 | 1 | 0.860726171 | 0.259627383 | 0.004512986 | 2.642359446 | 0.206198102 | 4.31686616 |
| TCGA-55-8505 | NOT | 440 | 0 | 0.894448708 | 0.689581921 | 0.09589354 | 0.629220143 | 0.502032085 | 3.748431108 |
| TCGA-05-4433 | YES | 730 | 0 | 0.897644238 | 0 | 0.027960633 | 2.568551643 | 0.106459991 | 4.821914714 |
| TCGA-05-4425 | NOT | 669 | 0 | 0.90212339 | 0.275145108 | 0.221200971 | 1.400145602 | 0.091051 | 1.539623708 |
| TCGA-62-8402 | YES | 1498 | 1 | 0.90464455 | 0.8777076 | 0.1736118 | 1.189634 | 0.200311 | 0.7742061 |
| TCGA-38-4631 | YES | 354 | 1 | 0.909715984 | 0 | 0.1677609 | 8.221278 | 0.4645447 | 0.3506787 |
| TCGA-MP-A4T8 | NOT | 161 | 1 | 0.920274895 | 4.08541 | 0.01753453 | 0.05310253 | 2.603743 | 0.4031859 |
| TCGA-44-6779 | YES | 500 | 1 | 0.928572587 | 1.156910335 | 0.05027513 | 3.281962325 | 0.829496827 | 2.543238846 |
| TCGA-73-4666 | NOT | 800 | 0 | 0.951981521 | 0.219277 | 0.2613664 | 3.188133 | 0.4561112 | 1.427331 |
| TCGA-69-8255 | YES | 129 | 0 | 0.95429539 | 0 | 0.1838951 | 7.463655 | 0.3052066 | 0.4120031 |
| TCGA-78-7220 | YES | 807 | 1 | 0.968851859 | 0.8271506 | 0.0743689 | 1.721704 | 0.2265274 | 2.736041 |
| TCGA-78-8660 | YES | 321 | 1 | 0.991058729 | 0.909014648 | 0.052669976 | 3.190173357 | 0.490210625 | 2.852776422 |
| TCGA-69-7761 | NOT | 186 | 0 | 0.991354674 | 0.06279376 | 0.02728789 | 3.239494 | 0.04155941 | 4.718447 |
| TCGA-05-4390 | NOT | 1126 | 0 | 0.997922234 | 1.46821 | 0.006716112 | 3.172954 | 0.2045726 | 0.9883464 |
| TCGA-95-A4VK | YES | 651 | 0 | 1.039895574 | 0.95264 | 0.1405033 | 0.7497043 | 0.07642365 | 1.938425 |
| TCGA-44-7661 | YES | 557 | 1 | 1.066036421 | 1.118835712 | 0.082894044 | 5.085187594 | 0.485567263 | 1.084983063 |
| TCGA-05-4250 | YES | 121 | 1 | 1.082079665 | 0.2147779 | 0.1730934 | 5.180386 | 0.258452 | 2.029061 |
| TCGA-05-4418 | YES | 274 | 1 | 1.126180694 | 1.184592 | 0.07759014 | 4.169358 | 0.1363496 | 0.9332186 |
| TCGA-49-4507 | NOT | 268 | 1 | 1.142748016 | 2.15734 | 0.02151643 | 3.289107 | 0.4681356 | 0.2261692 |
| TCGA-62-A471 | YES | 1246 | 0 | 1.163185848 | 2.709875 | 0 | 1.5783 | 1.10663 | 1.751418 |
| TCGA-55-7284 | YES | 243 | 1 | 1.176463005 | 0.055723077 | 0.08394613 | 2.503159145 | 0.049173005 | 5.642248188 |
| TCGA-78-7150 | YES | 666 | 1 | 1.178906719 | 0.693217092 | 0.067479334 | 2.880476701 | 0.183519482 | 3.989849485 |
| TCGA-55-8089 | NOT | 702 | 1 | 1.204110562 | 0.903915023 | 0.130379061 | 3.914723944 | 0.025457319 | 1.41440026 |
| TCGA-05-4397 | YES | 731 | 1 | 1.294929008 | 0.1428907 | 0.05645012 | 5.352084 | 0.8941238 | 7.372665 |
| TCGA-95-8494 | YES | 84 | 0 | 1.380843836 | 0.6194766 | 0.02019016 | 14.70197 | 0.71749 | 0.9284979 |
| TCGA-49-AAR9 | YES | 260 | 1 | 1.399808937 | 0.3767896 | 0.0935652 | 4.118137 | 0 | 4.948271 |
| TCGA-44-2668 | YES | 761 | 1 | 1.416650061 | 4.469731 | 0.03744353 | 0.4157856 | 7.48472 | 16.18624 |
| TCGA-50-5072 | YES | 250 | 1 | 1.445259119 | 0.7316897 | 0.1135592 | 8.382209 | 0.5534416 | 2.840943 |
| TCGA-49-6742 | NOT | 488 | 1 | 1.490462656 | 0.6550786 | 0.1067525 | 10.34545 | 0.758725 | 2.945579 |
| TCGA-55-A494 | NOT | 481 | 0 | 1.732321699 | 1.230685 | 0.01860212 | 7.361197 | 0.28331 | 4.619526 |
| TCGA-73-4670 | NOT | 131 | 0 | 2.052884573 | 3.699574 | 0.01418559 | 3.685051 | 0.3780811 | 1.369961 |
| TCGA-MP-A4TA | NOT | 950 | 1 | 2.209973492 | 0.848345487 | 0.24130486 | 11.15117642 | 1.156966138 | 6.041724226 |
| TCGA-05-5429 | YES | 275 | 1 | 2.686244846 | 0.041683342 | 0.050719396 | 26.41944455 | 0.275876976 | 3.532019995 |
| TCGA-55-8620 | YES | 375 | 1 | 2.803281721 | 0.03663383 | 1.127117 | 2.519891 | 0.09698291 | 1.05424 |
| TCGA-L9-A5IP | YES | 58 | 1 | 4.001290832 | 7.720560948 | 0.055210111 | 2.160755943 | 0.355743829 | 2.460860935 |

**Supplementary Table 3. Univariate and multivariate Cox proportional hazards regression analysis of 5-lncRNA signature and clinical risk factors in the “primary dataset”.**

| **Characteristic** | **Univariate analysis** | | **Multivariate analysis** | |
| --- | --- | --- | --- | --- |
|  | **HR (95%CI)** | **P-Value** | **HR (95%CI)** | **P Value** |
| Age | 0.990(0.967-1.014) | 0.454 | 1.004(0.980-1.030) | 0.707 |
| Gender (female *vs.* male) | 0.867(0.556-1.352) | 0.530 | 0.847(0.537-1.335) | 0.475 |
| TNM stage (I-Ⅳ) | 1.853(1.468-2.340) | **<0.001** | 1.736(1.128-2.672) | **0.012** |
| Tumor stage (T1-T4) | 1.503(1.100-2.054) | **0.010** | 1.120(0.798-1.572) | 0.509 |
| Lymph node metastasis (N0-N3) | 1.703(1.312-2.211) | **<0.001** | 0.980(0.623-1.542) | 0.933 |
| Risk score | 1.090(1.056-1.125) | **<0.001** | 1.065(1.028-1.102) | **<0.001** |

Notes: Bold values indicate statistical significance (*P* < 0.05).

Abbreviations: HR, hazard ratio; CI, confidence interval.

**Supplementary Table 4. The Case ID involved in this study.**

| **TCGA Case ID of 59 normal samples** | **TCGA Case ID of 479 LUAD samples** | **GEO Case ID of 58 LUAD samples (GSE3141)** | **GEO Case ID of 40 LUAD samples (GSE19188)** |
| --- | --- | --- | --- |
| TCGA-38-4626-11A | TCGA-44-4112 | GSM70234 | GSM475679 |
| TCGA-44-6148-11A | TCGA-44-2662 | GSM70203 | GSM475799 |
| TCGA-44-5645-11A | TCGA-44-3917 | GSM70179 | GSM475710 |
| TCGA-44-2668-11A | TCGA-62-8399 | GSM70140 | GSM475773 |
| TCGA-91-6829-11A | TCGA-35-3615 | GSM70171 | GSM475706 |
| TCGA-91-6849-11A | TCGA-49-6743 | GSM70131 | GSM475698 |
| TCGA-50-5935-11A | TCGA-86-8674 | GSM70150 | GSM475683 |
| TCGA-44-6146-11A | TCGA-55-7995 | GSM70197 | GSM475747 |
| TCGA-49-6744-11A | TCGA-44-2655 | GSM70229 | GSM475779 |
| TCGA-49-6745-11A | TCGA-05-4384 | GSM70177 | GSM475792 |
| TCGA-91-6836-11A | TCGA-44-6146 | GSM70170 | GSM475796 |
| TCGA-55-6968-11A | TCGA-MP-A5C7 | GSM70219 | GSM475709 |
| TCGA-44-6778-11A | TCGA-78-7156 | GSM70161 | GSM475772 |
| TCGA-55-6982-11A | TCGA-67-3773 | GSM70181 | GSM475788 |
| TCGA-55-6984-11A | TCGA-05-4382 | GSM70136 | GSM475700 |
| TCGA-55-6981-11A | TCGA-55-A492 | GSM70184 | GSM475687 |
| TCGA-91-6835-11A | TCGA-64-1677 | GSM70220 | GSM475765 |
| TCGA-44-6776-11A | TCGA-55-A48Y | GSM70190 | GSM475795 |
| TCGA-44-2665-11A | TCGA-44-2656 | GSM70223 | GSM475696 |
| TCGA-91-6847-11A | TCGA-44-A47A | GSM70129 | GSM475685 |
| TCGA-44-3396-11A | TCGA-78-8640 | GSM70195 | GSM475794 |
| TCGA-50-5931-11A | TCGA-73-4676 | GSM70163 | GSM475780 |
| TCGA-44-6145-11A | TCGA-55-6972 | GSM70144 | GSM475787 |
| TCGA-44-6144-11A | TCGA-55-6987 | GSM70201 | GSM475808 |
| TCGA-55-6970-11A | TCGA-64-1676 | GSM70183 | GSM475803 |
| TCGA-55-6969-11A | TCGA-55-7727 | GSM70175 | GSM475768 |
| TCGA-91-6828-11A | TCGA-86-8278 | GSM70224 | GSM475784 |
| TCGA-49-6742-11A | TCGA-62-A46V | GSM70155 | GSM475703 |
| TCGA-38-4627-11A | TCGA-50-5068 | GSM70196 | GSM475797 |
| TCGA-49-4512-11A | TCGA-86-8673 | GSM70225 | GSM475712 |
| TCGA-55-6983-11A | TCGA-71-6725 | GSM70199 | GSM475761 |
| TCGA-49-6761-11A | TCGA-49-4486 | GSM70174 | GSM475701 |
| TCGA-55-6975-11A | TCGA-05-5423 | GSM70209 | GSM475692 |
| TCGA-44-2655-11A | TCGA-55-7911 | GSM70172 | GSM475708 |
| TCGA-44-2657-11A | TCGA-67-3770 | GSM70130 | GSM475770 |
| TCGA-50-5936-11A | TCGA-MN-A4N4 | GSM70222 | GSM475691 |
| TCGA-50-5932-11A | TCGA-69-7760 | GSM70145 | GSM475689 |
| TCGA-44-2661-11A | TCGA-95-7948 | GSM70208 | GSM475715 |
| TCGA-49-6743-11A | TCGA-53-7626 | GSM70214 | GSM475681 |
| TCGA-55-6971-11A | TCGA-78-7149 | GSM70226 | GSM475810 |
| TCGA-50-6595-11A | TCGA-L9-A444 | GSM70193 |  |
| TCGA-55-6980-11A | TCGA-J2-A4AD | GSM70204 |  |
| TCGA-44-2662-11A | TCGA-44-3398 | GSM70156 |  |
| TCGA-55-6986-11A | TCGA-55-7994 | GSM70151 |  |
| TCGA-38-4625-11A | TCGA-64-1680 | GSM70154 |  |
| TCGA-91-6831-11A | TCGA-97-8179 | GSM70173 |  |
| TCGA-50-5933-11A | TCGA-44-6147 | GSM70233 |  |
| TCGA-50-5939-11A | TCGA-O1-A52J | GSM70164 |  |
| TCGA-73-4676-11A | TCGA-86-A4JF | GSM70235 |  |
| TCGA-55-6979-11A | TCGA-44-5643 | GSM70138 |  |
| TCGA-50-5930-11A | TCGA-91-8496 | GSM70148 |  |
| TCGA-38-4632-11A | TCGA-67-6217 | GSM70194 |  |
| TCGA-49-4490-11A | TCGA-73-7498 | GSM70127 |  |
| TCGA-44-3398-11B | TCGA-91-6849 | GSM70218 |  |
| TCGA-44-6777-11A | TCGA-97-A4M2 | GSM70153 |  |
| TCGA-55-6978-11A | TCGA-86-A4P7 | GSM70159 |  |
| TCGA-55-6972-11A | TCGA-97-7937 | GSM70169 |  |
| TCGA-55-6985-11A | TCGA-62-A472 | GSM70227 |  |
| TCGA-44-6147-11A | TCGA-55-6979 |  |  |
|  | TCGA-05-4424 |  |  |
|  | TCGA-78-7167 |  |  |
|  | TCGA-64-5781 |  |  |
|  | TCGA-97-7546 |  |  |
|  | TCGA-50-5049 |  |  |
|  | TCGA-MP-A4TK |  |  |
|  | TCGA-49-AARQ |  |  |
|  | TCGA-50-5051 |  |  |
|  | TCGA-44-7659 |  |  |
|  | TCGA-67-6215 |  |  |
|  | TCGA-78-8655 |  |  |
|  | TCGA-49-AAQV |  |  |
|  | TCGA-MP-A4TC |  |  |
|  | TCGA-69-A59K |  |  |
|  | TCGA-35-5375 |  |  |
|  | TCGA-55-8301 |  |  |
|  | TCGA-97-8177 |  |  |
|  | TCGA-MP-A4TH |  |  |
|  | TCGA-J2-8192 |  |  |
|  | TCGA-86-8280 |  |  |
|  | TCGA-55-8208 |  |  |
|  | TCGA-55-8514 |  |  |
|  | TCGA-67-6216 |  |  |
|  | TCGA-MP-A4TD |  |  |
|  | TCGA-55-8508 |  |  |
|  | TCGA-44-2665 |  |  |
|  | TCGA-49-4488 |  |  |
|  | TCGA-49-AAR0 |  |  |
|  | TCGA-78-8662 |  |  |
|  | TCGA-67-3771 |  |  |
|  | TCGA-93-7347 |  |  |
|  | TCGA-86-8073 |  |  |
|  | TCGA-97-A4M3 |  |  |
|  | TCGA-55-7281 |  |  |
|  | TCGA-MP-A4SY |  |  |
|  | TCGA-44-A47G |  |  |
|  | TCGA-NJ-A7XG |  |  |
|  | TCGA-86-8076 |  |  |
|  | TCGA-05-5428 |  |  |
|  | TCGA-49-AAR4 |  |  |
|  | TCGA-MP-A4SV |  |  |
|  | TCGA-97-A4M6 |  |  |
|  | TCGA-86-8074 |  |  |
|  | TCGA-86-8585 |  |  |
|  | TCGA-44-2659 |  |  |
|  | TCGA-44-6776 |  |  |
|  | TCGA-55-8510 |  |  |
|  | TCGA-55-7726 |  |  |
|  | TCGA-55-8085 |  |  |
|  | TCGA-55-7816 |  |  |
|  | TCGA-55-7724 |  |  |
|  | TCGA-50-7109 |  |  |
|  | TCGA-69-8253 |  |  |
|  | TCGA-55-8207 |  |  |
|  | TCGA-86-8671 |  |  |
|  | TCGA-93-A4JQ |  |  |
|  | TCGA-J2-8194 |  |  |
|  | TCGA-J2-A4AE |  |  |
|  | TCGA-86-8075 |  |  |
|  | TCGA-44-6774 |  |  |
|  | TCGA-97-8174 |  |  |
|  | TCGA-J2-A4AG |  |  |
|  | TCGA-44-3919 |  |  |
|  | TCGA-50-8457 |  |  |
|  | TCGA-67-3774 |  |  |
|  | TCGA-49-4487 |  |  |
|  | TCGA-64-5779 |  |  |
|  | TCGA-55-6980 |  |  |
|  | TCGA-55-7815 |  |  |
|  | TCGA-49-4512 |  |  |
|  | TCGA-55-7283 |  |  |
|  | TCGA-78-7537 |  |  |
|  | TCGA-49-4505 |  |  |
|  | TCGA-97-8175 |  |  |
|  | TCGA-50-5055 |  |  |
|  | TCGA-99-AA5R |  |  |
|  | TCGA-78-7158 |  |  |
|  | TCGA-95-7567 |  |  |
|  | TCGA-78-7162 |  |  |
|  | TCGA-50-5935 |  |  |
|  | TCGA-05-4430 |  |  |
|  | TCGA-67-3772 |  |  |
|  | TCGA-38-7271 |  |  |
|  | TCGA-55-8614 |  |  |
|  | TCGA-97-A4M0 |  |  |
|  | TCGA-97-7938 |  |  |
|  | TCGA-55-8091 |  |  |
|  | TCGA-44-6777 |  |  |
|  | TCGA-62-8397 |  |  |
|  | TCGA-91-6835 |  |  |
|  | TCGA-86-7714 |  |  |
|  | TCGA-44-A4SU |  |  |
|  | TCGA-95-A4VP |  |  |
|  | TCGA-73-4659 |  |  |
|  | TCGA-50-5944 |  |  |
|  | TCGA-38-4627 |  |  |
|  | TCGA-97-8552 |  |  |
|  | TCGA-49-AARO |  |  |
|  | TCGA-55-6986 |  |  |
|  | TCGA-97-A4M1 |  |  |
|  | TCGA-97-7553 |  |  |
|  | TCGA-73-7499 |  |  |
|  | TCGA-86-7953 |  |  |
|  | TCGA-78-7152 |  |  |
|  | TCGA-49-AAR2 |  |  |
|  | TCGA-69-7763 |  |  |
|  | TCGA-55-8616 |  |  |
|  | TCGA-49-6744 |  |  |
|  | TCGA-50-8460 |  |  |
|  | TCGA-91-A4BD |  |  |
|  | TCGA-55-8097 |  |  |
|  | TCGA-55-8512 |  |  |
|  | TCGA-L4-A4E6 |  |  |
|  | TCGA-78-8648 |  |  |
|  | TCGA-MP-A4T9 |  |  |
|  | TCGA-44-A47B |  |  |
|  | TCGA-44-7672 |  |  |
|  | TCGA-55-1596 |  |  |
|  | TCGA-86-A456 |  |  |
|  | TCGA-44-2661 |  |  |
|  | TCGA-64-1681 |  |  |
|  | TCGA-49-AAR3 |  |  |
|  | TCGA-44-2657 |  |  |
|  | TCGA-55-7573 |  |  |
|  | TCGA-93-A4JP |  |  |
|  | TCGA-55-8094 |  |  |
|  | TCGA-MP-A4TI |  |  |
|  | TCGA-78-7145 |  |  |
|  | TCGA-44-8117 |  |  |
|  | TCGA-78-7163 |  |  |
|  | TCGA-05-4422 |  |  |
|  | TCGA-69-7978 |  |  |
|  | TCGA-99-8028 |  |  |
|  | TCGA-86-8668 |  |  |
|  | TCGA-49-4501 |  |  |
|  | TCGA-55-6543 |  |  |
|  | TCGA-55-7574 |  |  |
|  | TCGA-78-7633 |  |  |
|  | TCGA-86-A4P8 |  |  |
|  | TCGA-55-8299 |  |  |
|  | TCGA-86-8056 |  |  |
|  | TCGA-50-5941 |  |  |
|  | TCGA-69-7979 |  |  |
|  | TCGA-55-6642 |  |  |
|  | TCGA-L9-A50W |  |  |
|  | TCGA-44-A4SS |  |  |
|  | TCGA-53-7813 |  |  |
|  | TCGA-95-7043 |  |  |
|  | TCGA-49-AARR |  |  |
|  | TCGA-49-4510 |  |  |
|  | TCGA-MP-A4SW |  |  |
|  | TCGA-91-6847 |  |  |
|  | TCGA-91-8497 |  |  |
|  | TCGA-05-4426 |  |  |
|  | TCGA-73-4662 |  |  |
|  | TCGA-50-5942 |  |  |
|  | TCGA-05-4402 |  |  |
|  | TCGA-44-7671 |  |  |
|  | TCGA-95-7039 |  |  |
|  | TCGA-55-8513 |  |  |
|  | TCGA-95-8039 |  |  |
|  | TCGA-05-5420 |  |  |
|  | TCGA-55-7570 |  |  |
|  | TCGA-86-7954 |  |  |
|  | TCGA-55-7725 |  |  |
|  | TCGA-L9-A443 |  |  |
|  | TCGA-38-4628 |  |  |
|  | TCGA-38-4630 |  |  |
|  | TCGA-86-8279 |  |  |
|  | TCGA-78-7539 |  |  |
|  | TCGA-49-4490 |  |  |
|  | TCGA-73-4658 |  |  |
|  | TCGA-NJ-A55A |  |  |
|  | TCGA-55-7914 |  |  |
|  | TCGA-91-6840 |  |  |
|  | TCGA-86-8054 |  |  |
|  | TCGA-78-7153 |  |  |
|  | TCGA-05-4415 |  |  |
|  | TCGA-93-7348 |  |  |
|  | TCGA-44-3918 |  |  |
|  | TCGA-55-A48Z |  |  |
|  | TCGA-78-7535 |  |  |
|  | TCGA-44-6775 |  |  |
|  | TCGA-86-7711 |  |  |
|  | TCGA-97-7554 |  |  |
|  | TCGA-50-6590 |  |  |
|  | TCGA-55-8206 |  |  |
|  | TCGA-93-A4JO |  |  |
|  | TCGA-55-6968 |  |  |
|  | TCGA-MP-A4T6 |  |  |
|  | TCGA-55-6983 |  |  |
|  | TCGA-55-8619 |  |  |
|  | TCGA-L9-A8F4 |  |  |
|  | TCGA-05-4249 |  |  |
|  | TCGA-50-6597 |  |  |
|  | TCGA-05-4427 |  |  |
|  | TCGA-55-7227 |  |  |
|  | TCGA-55-A4DF |  |  |
|  | TCGA-55-A491 |  |  |
|  | TCGA-44-6145 |  |  |
|  | TCGA-55-8087 |  |  |
|  | TCGA-44-6148 |  |  |
|  | TCGA-55-6985 |  |  |
|  | TCGA-44-7669 |  |  |
|  | TCGA-MP-A4T7 |  |  |
|  | TCGA-86-8669 |  |  |
|  | TCGA-S2-AA1A |  |  |
|  | TCGA-44-2666 |  |  |
|  | TCGA-95-7562 |  |  |
|  | TCGA-NJ-A4YI |  |  |
|  | TCGA-97-A4LX |  |  |
|  | TCGA-69-7980 |  |  |
|  | TCGA-NJ-A4YG |  |  |
|  | TCGA-62-8395 |  |  |
|  | TCGA-95-7944 |  |  |
|  | TCGA-69-7764 |  |  |
|  | TCGA-64-1679 |  |  |
|  | TCGA-62-A46R |  |  |
|  | TCGA-55-A4DG |  |  |
|  | TCGA-55-6971 |  |  |
|  | TCGA-MP-A4T4 |  |  |
|  | TCGA-55-8204 |  |  |
|  | TCGA-86-8055 |  |  |
|  | TCGA-64-5775 |  |  |
|  | TCGA-78-7143 |  |  |
|  | TCGA-50-5931 |  |  |
|  | TCGA-NJ-A4YF |  |  |
|  | TCGA-44-7660 |  |  |
|  | TCGA-05-4432 |  |  |
|  | TCGA-55-8090 |  |  |
|  | TCGA-NJ-A55R |  |  |
|  | TCGA-44-A479 |  |  |
|  | TCGA-86-8358 |  |  |
|  | TCGA-78-7160 |  |  |
|  | TCGA-L9-A743 |  |  |
|  | TCGA-05-4398 |  |  |
|  | TCGA-97-8547 |  |  |
|  | TCGA-44-3396 |  |  |
|  | TCGA-NJ-A55O |  |  |
|  | TCGA-97-7547 |  |  |
|  | TCGA-86-6851 |  |  |
|  | TCGA-64-5778 |  |  |
|  | TCGA-44-7667 |  |  |
|  | TCGA-50-5066 |  |  |
|  | TCGA-44-8120 |  |  |
|  | TCGA-49-4514 |  |  |
|  | TCGA-05-4417 |  |  |
|  | TCGA-55-6978 |  |  |
|  | TCGA-MP-A4TJ |  |  |
|  | TCGA-35-4123 |  |  |
|  | TCGA-55-A57B |  |  |
|  | TCGA-50-6595 |  |  |
|  | TCGA-50-8459 |  |  |
|  | TCGA-64-5815 |  |  |
|  | TCGA-05-4420 |  |  |
|  | TCGA-91-6831 |  |  |
|  | TCGA-50-5044 |  |  |
|  | TCGA-99-8032 |  |  |
|  | TCGA-99-8033 |  |  |
|  | TCGA-99-7458 |  |  |
|  | TCGA-55-6982 |  |  |
|  | TCGA-55-8621 |  |  |
|  | TCGA-78-7147 |  |  |
|  | TCGA-62-A46Y |  |  |
|  | TCGA-86-7713 |  |  |
|  | TCGA-55-7910 |  |  |
|  | TCGA-62-A470 |  |  |
|  | TCGA-38-4632 |  |  |
|  | TCGA-73-4668 |  |  |
|  | TCGA-78-7146 |  |  |
|  | TCGA-35-4122 |  |  |
|  | TCGA-86-7955 |  |  |
|  | TCGA-91-A4BC |  |  |
|  | TCGA-91-6848 |  |  |
|  | TCGA-97-A4M7 |  |  |
|  | TCGA-50-5933 |  |  |
|  | TCGA-L4-A4E5 |  |  |
|  | TCGA-55-6981 |  |  |
|  | TCGA-93-A4JN |  |  |
|  | TCGA-49-6745 |  |  |
|  | TCGA-86-8359 |  |  |
|  | TCGA-44-7662 |  |  |
|  | TCGA-86-6562 |  |  |
|  | TCGA-78-7155 |  |  |
|  | TCGA-NJ-A4YP |  |  |
|  | TCGA-69-7973 |  |  |
|  | TCGA-50-5946 |  |  |
|  | TCGA-97-A4M5 |  |  |
|  | TCGA-55-8506 |  |  |
|  | TCGA-MN-A4N1 |  |  |
|  | TCGA-49-4494 |  |  |
|  | TCGA-78-7536 |  |  |
|  | TCGA-78-7148 |  |  |
|  | TCGA-86-7701 |  |  |
|  | TCGA-73-4675 |  |  |
|  | TCGA-38-4625 |  |  |
|  | TCGA-55-1592 |  |  |
|  | TCGA-NJ-A4YQ |  |  |
|  | TCGA-91-7771 |  |  |
|  | TCGA-55-8203 |  |  |
|  | TCGA-55-7903 |  |  |
|  | TCGA-86-8672 |  |  |
|  | TCGA-49-4506 |  |  |
|  | TCGA-78-7159 |  |  |
|  | TCGA-69-8453 |  |  |
|  | TCGA-55-8615 |  |  |
|  | TCGA-55-7907 |  |  |
|  | TCGA-55-6970 |  |  |
|  | TCGA-MP-A4TF |  |  |
|  | TCGA-71-8520 |  |  |
|  | TCGA-78-7161 |  |  |
|  | TCGA-78-7542 |  |  |
|  | TCGA-MN-A4N5 |  |  |
|  | TCGA-97-7941 |  |  |
|  | TCGA-97-8176 |  |  |
|  | TCGA-62-A46P |  |  |
|  | TCGA-91-6829 |  |  |
|  | TCGA-05-4396 |  |  |
|  | TCGA-55-6712 |  |  |
|  | TCGA-86-A4D0 |  |  |
|  | TCGA-50-5932 |  |  |
|  | TCGA-62-A46S |  |  |
|  | TCGA-05-4405 |  |  |
|  | TCGA-50-6593 |  |  |
|  | TCGA-95-7947 |  |  |
|  | TCGA-55-A48X |  |  |
|  | TCGA-50-5936 |  |  |
|  | TCGA-55-8096 |  |  |
|  | TCGA-99-8025 |  |  |
|  | TCGA-97-8171 |  |  |
|  | TCGA-91-8499 |  |  |
|  | TCGA-05-5715 |  |  |
|  | TCGA-91-6836 |  |  |
|  | TCGA-49-6767 |  |  |
|  | TCGA-49-AARN |  |  |
|  | TCGA-44-7670 |  |  |
|  | TCGA-55-8507 |  |  |
|  | TCGA-50-6673 |  |  |
|  | TCGA-97-8172 |  |  |
|  | TCGA-49-AARE |  |  |
|  | TCGA-55-7728 |  |  |
|  | TCGA-55-8205 |  |  |
|  | TCGA-50-5939 |  |  |
|  | TCGA-83-5908 |  |  |
|  | TCGA-38-4629 |  |  |
|  | TCGA-91-6830 |  |  |
|  | TCGA-44-8119 |  |  |
|  | TCGA-50-6594 |  |  |
|  | TCGA-69-7974 |  |  |
|  | TCGA-55-8092 |  |  |
|  | TCGA-64-5774 |  |  |
|  | TCGA-73-A9RS |  |  |
|  | TCGA-55-6984 |  |  |
|  | TCGA-95-A4VN |  |  |
|  | TCGA-55-1594 |  |  |
|  | TCGA-50-5930 |  |  |
|  | TCGA-55-8511 |  |  |
|  | TCGA-55-7913 |  |  |
|  | TCGA-55-A493 |  |  |
|  | TCGA-93-8067 |  |  |
|  | TCGA-97-7552 |  |  |
|  | TCGA-05-4389 |  |  |
|  | TCGA-05-5425 |  |  |
|  | TCGA-05-4434 |  |  |
|  | TCGA-53-A4EZ |  |  |
|  | TCGA-78-7166 |  |  |
|  | TCGA-38-A44F |  |  |
|  | TCGA-44-6778 |  |  |
|  | TCGA-55-A490 |  |  |
|  | TCGA-91-6828 |  |  |
|  | TCGA-L9-A7SV |  |  |
|  | TCGA-55-7576 |  |  |
|  | TCGA-55-8302 |  |  |
|  | TCGA-78-7540 |  |  |
|  | TCGA-49-6761 |  |  |
|  | TCGA-50-6592 |  |  |
|  | TCGA-4B-A93V |  |  |
|  | TCGA-50-6591 |  |  |
|  | TCGA-62-A46O |  |  |
|  | TCGA-62-8398 |  |  |
|  | TCGA-55-6975 |  |  |
|  | TCGA-78-7154 |  |  |
|  | TCGA-MP-A4TE |  |  |
|  | TCGA-05-4403 |  |  |
|  | TCGA-53-7624 |  |  |
|  | TCGA-62-8394 |  |  |
|  | TCGA-55-8505 |  |  |
|  | TCGA-05-4433 |  |  |
|  | TCGA-05-4425 |  |  |
|  | TCGA-62-8402 |  |  |
|  | TCGA-38-4631 |  |  |
|  | TCGA-MP-A4T8 |  |  |
|  | TCGA-44-6779 |  |  |
|  | TCGA-73-4666 |  |  |
|  | TCGA-69-8255 |  |  |
|  | TCGA-78-7220 |  |  |
|  | TCGA-78-8660 |  |  |
|  | TCGA-69-7761 |  |  |
|  | TCGA-05-4390 |  |  |
|  | TCGA-95-A4VK |  |  |
|  | TCGA-44-7661 |  |  |
|  | TCGA-05-4250 |  |  |
|  | TCGA-05-4418 |  |  |
|  | TCGA-49-4507 |  |  |
|  | TCGA-62-A471 |  |  |
|  | TCGA-55-7284 |  |  |
|  | TCGA-78-7150 |  |  |
|  | TCGA-55-8089 |  |  |
|  | TCGA-05-4397 |  |  |
|  | TCGA-95-8494 |  |  |
|  | TCGA-49-AAR9 |  |  |
|  | TCGA-44-2668 |  |  |
|  | TCGA-50-5072 |  |  |
|  | TCGA-49-6742 |  |  |
|  | TCGA-55-A494 |  |  |
|  | TCGA-73-4670 |  |  |
|  | TCGA-MP-A4TA |  |  |
|  | TCGA-05-5429 |  |  |
|  | TCGA-55-8620 |  |  |
|  | TCGA-L9-A5IP |  |  |

**Supplementary Table 5. lncRNA-mRNA co-expression data.**

| **lncRNA** | **mRNA** | **correlation coefficient** | **P value** |
| --- | --- | --- | --- |
| AC026355.1 | AAK1 | 0.700980473 | 2.80E-80 |
| AL606489.1 | AAK1 | 0.511150619 | 6.00E-37 |
| AC026355.1 | ABCA1 | 0.576768451 | 9.20E-49 |
| AC026355.1 | ABCA5 | 0.577313766 | 7.15E-49 |
| AC026355.1 | ABCA6 | 0.656641988 | 2.64E-67 |
| AC026355.1 | ABCA9 | 0.634451208 | 1.33E-61 |
| AC026355.1 | ABCC9 | 0.526816232 | 1.55E-39 |
| AC026355.1 | ABHD13 | 0.50282653 | 1.26E-35 |
| AC026355.1 | ABHD18 | 0.529393682 | 5.63E-40 |
| AC026355.1 | ABL2 | 0.640430055 | 4.30E-63 |
| AC026355.1 | ABRAXAS1 | 0.509508477 | 1.10E-36 |
| AL606489.1 | ACOT11 | 0.533338661 | 1.18E-40 |
| AC026355.1 | ACTR3C | 0.689153658 | 1.37E-76 |
| AC026355.1 | ACVR2A | 0.529036598 | 6.48E-40 |
| AC026355.1 | AFF1 | 0.59905834 | 2.04E-53 |
| AL606489.1 | AFF1 | 0.507560849 | 2.25E-36 |
| AC026355.1 | AFF4 | 0.693874315 | 4.85E-78 |
| AC026355.1 | AGO2 | 0.507131855 | 2.64E-36 |
| AC026355.1 | AGO3 | 0.744858857 | 1.00E-95 |
| AC026355.1 | AHI1 | 0.694020853 | 4.36E-78 |
| AC026355.1 | AKAP11 | 0.620140175 | 3.63E-58 |
| AC026355.1 | AKAP9 | 0.634190566 | 1.54E-61 |
| AL606489.1 | AKAP9 | 0.500787678 | 2.62E-35 |
| AC026355.1 | ALG10 | 0.527715849 | 1.09E-39 |
| AC026355.1 | ALG10B | 0.604859176 | 1.09E-54 |
| AL606489.1 | ALG11 | 0.508029831 | 1.90E-36 |
| AC026355.1 | ALG13 | 0.720357413 | 9.82E-87 |
| AC026355.1 | ALMS1 | 0.631669958 | 6.39E-61 |
| AC026355.1 | ALPK1 | 0.512132841 | 4.17E-37 |
| AC026355.1 | ANAPC4 | 0.540341583 | 6.96E-42 |
| AC026355.1 | ANGEL2 | 0.616511305 | 2.53E-57 |
| AC026355.1 | ANGPTL1 | 0.66219958 | 8.25E-69 |
| AC026355.1 | ANKAR | 0.707473327 | 2.20E-82 |
| AL606489.1 | ANKAR | 0.528564922 | 7.79E-40 |
| AC026355.1 | ANKHD1 | 0.746434048 | 2.45E-96 |
| AC026355.1 | ANKRD12 | 0.604063946 | 1.64E-54 |
| AC026355.1 | ANKRD26 | 0.616227347 | 2.94E-57 |
| AC026355.1 | ANKRD28 | 0.541390133 | 4.53E-42 |
| AC026355.1 | ANKRD36 | 0.809342132 | 2.80E-125 |
| AL606489.1 | ANKRD36 | 0.523756326 | 5.08E-39 |
| AC026355.1 | ANKRD36C | 0.533539669 | 1.09E-40 |
| AC026355.1 | ANKRD44 | 0.599407535 | 1.72E-53 |
| AC026355.1 | ANKRD50 | 0.669676587 | 6.92E-71 |
| AC026355.1 | ANKRD61 | 0.702397789 | 9.83E-81 |
| AL606489.1 | ANKRD61 | 0.50018241 | 3.25E-35 |
| AC026355.1 | AP001931.1 | 0.593663704 | 2.95E-52 |
| AL606489.1 | AP001931.1 | 0.524498598 | 3.81E-39 |
| AC026355.1 | AP4E1 | 0.547141301 | 4.19E-43 |
| AC026355.1 | AP5M1 | 0.558848347 | 2.85E-45 |
| AC026355.1 | APC | 0.631106085 | 8.77E-61 |
| AC026355.1 | APPBP2 | 0.509627384 | 1.05E-36 |
| AC026355.1 | AQP11 | 0.580034333 | 2.02E-49 |
| AC026355.1 | ARAP2 | 0.599965833 | 1.30E-53 |
| AL606489.1 | ARAP2 | 0.551758842 | 5.99E-44 |
| AL606489.1 | ARFGEF2 | 0.513974615 | 2.10E-37 |
| AL606489.1 | ARHGAP32 | 0.518881346 | 3.29E-38 |
| AC026355.1 | ARHGAP5 | 0.555954951 | 9.96E-45 |
| AC026355.1 | ARHGEF38 | 0.512049119 | 4.30E-37 |
| AC026355.1 | ARID2 | 0.651602356 | 5.73E-66 |
| AL606489.1 | ARID2 | 0.536286891 | 3.61E-41 |
| AC026355.1 | ARIH1 | 0.603345842 | 2.36E-54 |
| LINC01843 | ARL14 | 0.542467739 | 2.91E-42 |
| AC026355.1 | ARL5A | 0.546115249 | 6.43E-43 |
| AC026355.1 | ARMC8 | 0.504138055 | 7.84E-36 |
| AL606489.1 | ASAP2 | 0.53093636 | 3.06E-40 |
| AC026355.1 | ASH1L | 0.648542426 | 3.61E-65 |
| AC026355.1 | ASXL2 | 0.523664961 | 5.26E-39 |
| AC026355.1 | ATAD2B | 0.715783043 | 3.67E-85 |
| AL606489.1 | ATAD2B | 0.520984251 | 1.48E-38 |
| AC026355.1 | ATAD5 | 0.585805391 | 1.32E-50 |
| AC026355.1 | ATF7IP | 0.553112635 | 3.37E-44 |
| AC026355.1 | ATF7IP2 | 0.626949133 | 8.86E-60 |
| AC026355.1 | ATG12 | 0.614866559 | 6.05E-57 |
| AC026355.1 | ATG2B | 0.565860652 | 1.30E-46 |
| AC026355.1 | ATM | 0.688099768 | 2.86E-76 |
| AL606489.1 | ATP11B | 0.630433934 | 1.28E-60 |
| AC026355.1 | ATP6AP1L | 0.524823052 | 3.36E-39 |
| AC026355.1 | ATP8B4 | 0.701945412 | 1.37E-80 |
| AC026355.1 | ATP9B | 0.60115275 | 7.15E-54 |
| AC026355.1 | ATR | 0.597253656 | 5.02E-53 |
| AL606489.1 | ATR | 0.527432724 | 1.22E-39 |
| AC026355.1 | ATRX | 0.630615389 | 1.15E-60 |
| AC026355.1 | ATXN1 | 0.698850929 | 1.33E-79 |
| AC026355.1 | ATXN2 | 0.554126505 | 2.18E-44 |
| AC026355.1 | ATXN3 | 0.611965965 | 2.78E-56 |
| AC026355.1 | ATXN7 | 0.639856411 | 5.99E-63 |
| AC026355.1 | AVL9 | 0.522564734 | 8.04E-39 |
| AC026355.1 | BAZ2B | 0.686884952 | 6.67E-76 |
| AL606489.1 | BAZ2B | 0.52444783 | 3.88E-39 |
| AC026355.1 | BBIP1 | 0.698601243 | 1.60E-79 |
| AC026355.1 | BBX | 0.522973127 | 6.87E-39 |
| AC026355.1 | BCLAF1 | 0.547220742 | 4.05E-43 |
| AC026355.1 | BCLAF3 | 0.555722359 | 1.10E-44 |
| AC026355.1 | BDP1 | 0.723217802 | 9.84E-88 |
| AC026355.1 | BIRC6 | 0.641591266 | 2.19E-63 |
| AL606489.1 | BIRC6 | 0.558511895 | 3.29E-45 |
| AC026355.1 | BMP2K | 0.603067574 | 2.72E-54 |
| AC026355.1 | BMPR2 | 0.574677057 | 2.41E-48 |
| AC026355.1 | BNIP2 | 0.549952811 | 1.29E-43 |
| AC026355.1 | BOD1L1 | 0.510908173 | 6.57E-37 |
| AC026355.1 | BPTF | 0.598493486 | 2.71E-53 |
| AC026355.1 | BRAF | 0.680602351 | 4.96E-74 |
| AC026355.1 | BRCA2 | 0.585419987 | 1.59E-50 |
| AL606489.1 | BRCA2 | 0.500612885 | 2.79E-35 |
| AC026355.1 | BRWD1 | 0.736031951 | 2.27E-92 |
| AC026355.1 | BRWD3 | 0.712645157 | 4.22E-84 |
| AC026355.1 | BTAF1 | 0.638384833 | 1.40E-62 |
| AC026355.1 | BTLA | 0.558235286 | 3.71E-45 |
| AC026355.1 | C16orf72 | 0.63189304 | 5.64E-61 |
| AC026355.1 | C18orf25 | 0.590714701 | 1.24E-51 |
| AC026355.1 | C2orf88 | 0.686121663 | 1.13E-75 |
| AC026355.1 | C5orf63 | 0.660651594 | 2.18E-68 |
| AC026355.1 | C8orf44 | 0.571758257 | 9.15E-48 |
| AC026355.1 | C9orf72 | 0.582931302 | 5.17E-50 |
| AC026355.1 | C9orf84 | 0.731233462 | 1.33E-90 |
| AC026355.1 | CAMKMT | 0.562591379 | 5.53E-46 |
| AC026355.1 | CAMSAP2 | 0.541499059 | 4.33E-42 |
| AC026355.1 | CAPN7 | 0.614388268 | 7.79E-57 |
| AC026355.1 | CARD8 | 0.591109696 | 1.03E-51 |
| AC026355.1 | CARF | 0.605047773 | 9.93E-55 |
| AC026355.1 | CASP8AP2 | 0.54665449 | 5.13E-43 |
| AC026355.1 | CBLB | 0.584877908 | 2.05E-50 |
| AC026355.1 | CBR4 | 0.584795284 | 2.14E-50 |
| AC026355.1 | CCDC102B | 0.523943342 | 4.72E-39 |
| AC026355.1 | CCDC122 | 0.561748442 | 8.01E-46 |
| AC026355.1 | CCDC14 | 0.552008463 | 5.39E-44 |
| AC026355.1 | CCDC18 | 0.6921676 | 1.63E-77 |
| AC026355.1 | CCDC200 | 0.62910917 | 2.67E-60 |
| AL606489.1 | CCDC200 | 0.570367454 | 1.72E-47 |
| AC026355.1 | CCDC39 | 0.540171839 | 7.46E-42 |
| AC026355.1 | CCDC66 | 0.608209674 | 1.96E-55 |
| AC026355.1 | CCDC82 | 0.514845011 | 1.51E-37 |
| AC026355.1 | CCDC88A | 0.571353821 | 1.10E-47 |
| AC026355.1 | CCNH | 0.695114132 | 1.99E-78 |
| AC026355.1 | CCNT2 | 0.638693861 | 1.17E-62 |
| AC026355.1 | CCSAP | 0.535796762 | 4.40E-41 |
| AC026355.1 | CCSER1 | 0.656858901 | 2.31E-67 |
| AC026355.1 | CDC73 | 0.551836085 | 5.80E-44 |
| AC026355.1 | CDK17 | 0.653786006 | 1.52E-66 |
| AC026355.1 | CENPC | 0.643643701 | 6.58E-64 |
| AC026355.1 | CEP120 | 0.525687487 | 2.40E-39 |
| AC026355.1 | CEP135 | 0.537176674 | 2.52E-41 |
| AC026355.1 | CEP152 | 0.525903748 | 2.21E-39 |
| AC026355.1 | CEP170 | 0.506020608 | 3.96E-36 |
| AC026355.1 | CEP192 | 0.523459093 | 5.69E-39 |
| AC026355.1 | CEP290 | 0.573000659 | 5.20E-48 |
| AC026355.1 | CEP295 | 0.568683222 | 3.67E-47 |
| AC026355.1 | CEP350 | 0.659744604 | 3.85E-68 |
| AC026355.1 | CEP57L1 | 0.514094458 | 2.00E-37 |
| AC026355.1 | CEP68 | 0.5509641 | 8.39E-44 |
| AC026355.1 | CEP83 | 0.54495171 | 1.04E-42 |
| AC026355.1 | CEP85L | 0.637710164 | 2.07E-62 |
| AL606489.1 | CEP85L | 0.525447702 | 2.64E-39 |
| AC026355.1 | CEP97 | 0.503971083 | 8.33E-36 |
| AC026355.1 | CEPT1 | 0.586446034 | 9.72E-51 |
| AC026355.1 | CFAP69 | 0.610171133 | 7.09E-56 |
| AC026355.1 | CHD1 | 0.674379636 | 3.19E-72 |
| AC026355.1 | CHD2 | 0.564905418 | 1.99E-46 |
| AC026355.1 | CHD9 | 0.662931918 | 5.20E-69 |
| AL606489.1 | CHD9 | 0.527686086 | 1.10E-39 |
| AC026355.1 | CHIC1 | 0.686929954 | 6.46E-76 |
| AC026355.1 | CHORDC1 | 0.598638789 | 2.52E-53 |
| LINC01843 | CHST4 | 0.542467536 | 2.91E-42 |
| AC026355.1 | CLASP2 | 0.641182472 | 2.78E-63 |
| AC026355.1 | CLEC2D | 0.638794238 | 1.11E-62 |
| AC026355.1 | CLHC1 | 0.710769729 | 1.79E-83 |
| AC026355.1 | CLK4 | 0.542418591 | 2.97E-42 |
| AC026355.1 | CLOCK | 0.716465923 | 2.15E-85 |
| AC026355.1 | CNOT4 | 0.608500249 | 1.69E-55 |
| AC026355.1 | CNOT6L | 0.675181408 | 1.87E-72 |
| AC026355.1 | COX20 | 0.648945486 | 2.84E-65 |
| AC026355.1 | CPEB2 | 0.603342916 | 2.36E-54 |
| AC026355.1 | CPEB4 | 0.628848992 | 3.09E-60 |
| AC026355.1 | CPLANE1 | 0.642177654 | 1.55E-63 |
| AC026355.1 | CPSF6 | 0.534545403 | 7.27E-41 |
| AC026355.1 | CRBN | 0.545739605 | 7.52E-43 |
| AC026355.1 | CREB1 | 0.638801873 | 1.10E-62 |
| AC026355.1 | CREBRF | 0.634346403 | 1.41E-61 |
| AC026355.1 | CRLF3 | 0.537937921 | 1.85E-41 |
| AC026355.1 | CRYBG3 | 0.596349287 | 7.86E-53 |
| AC026355.1 | CSNK1G3 | 0.579469216 | 2.62E-49 |
| AC026355.1 | CTDSPL2 | 0.568114176 | 4.74E-47 |
| AC026355.1 | CUL5 | 0.53635283 | 3.51E-41 |
| AC026355.1 | DCAF10 | 0.521467728 | 1.23E-38 |
| AC026355.1 | DCAF17 | 0.513357242 | 2.64E-37 |
| AC026355.1 | DCP2 | 0.661411644 | 1.35E-68 |
| AC026355.1 | DCTN4 | 0.535274199 | 5.42E-41 |
| AC026355.1 | DCUN1D1 | 0.507919391 | 1.98E-36 |
| AC026355.1 | DCUN1D4 | 0.591599548 | 8.09E-52 |
| AC026355.1 | DDHD1 | 0.625217232 | 2.30E-59 |
| AL606489.1 | DDX60L | 0.585718656 | 1.38E-50 |
| AC026355.1 | DENND1B | 0.697748158 | 2.97E-79 |
| AC026355.1 | DENND4A | 0.634894486 | 1.03E-61 |
| AL606489.1 | DENND4A | 0.52058605 | 1.72E-38 |
| AC026355.1 | DENND4C | 0.552871226 | 3.73E-44 |
| AC026355.1 | DGKE | 0.581445123 | 1.04E-49 |
| AL606489.1 | DGKE | 0.52623967 | 1.94E-39 |
| AC026355.1 | DGKH | 0.699214146 | 1.02E-79 |
| AL606489.1 | DGKH | 0.566960653 | 7.96E-47 |
| AC026355.1 | DHX36 | 0.525017497 | 3.11E-39 |
| AC026355.1 | DICER1 | 0.619790523 | 4.38E-58 |
| AL606489.1 | DIP2B | 0.518836023 | 3.35E-38 |
| AC026355.1 | DMTF1 | 0.724910485 | 2.49E-88 |
| AC026355.1 | DMXL1 | 0.674628356 | 2.70E-72 |
| AC026355.1 | DMXL2 | 0.667563026 | 2.71E-70 |
| AL606489.1 | DMXL2 | 0.504704168 | 6.38E-36 |
| AC026355.1 | DNAH14 | 0.602835555 | 3.06E-54 |
| AC026355.1 | DNAJB14 | 0.705683339 | 8.49E-82 |
| AC026355.1 | DNAJC13 | 0.500668238 | 2.74E-35 |
| AC026355.1 | DNAJC24 | 0.60622079 | 5.45E-55 |
| AC026355.1 | DOCK10 | 0.528750784 | 7.25E-40 |
| AC026355.1 | DOCK4 | 0.600365382 | 1.06E-53 |
| AC026355.1 | DOP1A | 0.58892765 | 2.95E-51 |
| AC026355.1 | DPH6 | 0.600307362 | 1.09E-53 |
| AC026355.1 | DPP8 | 0.60521716 | 9.11E-55 |
| AC026355.1 | DPY19L3 | 0.523362367 | 5.91E-39 |
| AC026355.1 | DTWD1 | 0.661949327 | 9.66E-69 |
| AC026355.1 | DTWD2 | 0.515673728 | 1.11E-37 |
| AC026355.1 | DYRK1A | 0.565385461 | 1.61E-46 |
| AC026355.1 | EBLN2 | 0.637844519 | 1.91E-62 |
| AL606489.1 | EDEM3 | 0.547029137 | 4.39E-43 |
| AC026355.1 | EEA1 | 0.577975319 | 5.26E-49 |
| AC026355.1 | EFCAB13 | 0.781675402 | 2.30E-111 |
| AC026355.1 | ELF2 | 0.586175603 | 1.11E-50 |
| AC026355.1 | ELK4 | 0.707192235 | 2.73E-82 |
| AC026355.1 | EMSY | 0.509830396 | 9.78E-37 |
| AC026355.1 | ENTPD1 | 0.524155474 | 4.35E-39 |
| AC026355.1 | EPC1 | 0.650292948 | 1.26E-65 |
| AC026355.1 | EPG5 | 0.502977059 | 1.19E-35 |
| AC026355.1 | EPM2AIP1 | 0.59741129 | 4.64E-53 |
| AC026355.1 | EPS15 | 0.563668779 | 3.44E-46 |
| AC026355.1 | ERBIN | 0.662948158 | 5.14E-69 |
| AC026355.1 | ERCC4 | 0.538248514 | 1.63E-41 |
| AC026355.1 | ERCC6 | 0.63327287 | 2.59E-61 |
| AC026355.1 | ERCC6L2 | 0.652872386 | 2.65E-66 |
| AL606489.1 | ERCC6L2 | 0.53182081 | 2.15E-40 |
| AC026355.1 | EVI5 | 0.566780188 | 8.62E-47 |
| AC026355.1 | EXOC8 | 0.534485789 | 7.44E-41 |
| AC026355.1 | EXOG | 0.608471308 | 1.71E-55 |
| AC026355.1 | FAM126B | 0.644512085 | 3.95E-64 |
| AC026355.1 | FAM135A | 0.533719024 | 1.01E-40 |
| AL606489.1 | FAM135A | 0.585221565 | 1.74E-50 |
| AC026355.1 | FAM13B | 0.591098154 | 1.03E-51 |
| AC026355.1 | FAM160B1 | 0.58316364 | 4.63E-50 |
| AC026355.1 | FAM172A | 0.552739005 | 3.95E-44 |
| AC026355.1 | FAM185A | 0.643351821 | 7.82E-64 |
| AC026355.1 | FAM208A | 0.609946652 | 7.97E-56 |
| AC026355.1 | FAM208B | 0.525613623 | 2.47E-39 |
| AC026355.1 | FAM214A | 0.671237087 | 2.51E-71 |
| AC026355.1 | FAM76B | 0.516158073 | 9.23E-38 |
| AC026355.1 | FAM92A | 0.531673609 | 2.28E-40 |
| AC026355.1 | FANCC | 0.511596387 | 5.09E-37 |
| AL606489.1 | FARP2 | 0.565881175 | 1.29E-46 |
| AC026355.1 | FAT4 | 0.506089363 | 3.86E-36 |
| AC026355.1 | FBXL13 | 0.606331337 | 5.15E-55 |
| AC026355.1 | FBXL2 | 0.541916047 | 3.65E-42 |
| AC026355.1 | FBXL20 | 0.517849409 | 4.87E-38 |
| AC026355.1 | FBXL4 | 0.635882113 | 5.88E-62 |
| AC026355.1 | FBXO11 | 0.553587811 | 2.75E-44 |
| AC026355.1 | FBXO30 | 0.527211308 | 1.32E-39 |
| AC026355.1 | FBXW7 | 0.565175589 | 1.76E-46 |
| AC026355.1 | FEM1C | 0.523782751 | 5.03E-39 |
| AC026355.1 | FER | 0.726975701 | 4.58E-89 |
| AL606489.1 | FGD4 | 0.600738335 | 8.81E-54 |
| AC026355.1 | FGFR1OP | 0.504108315 | 7.92E-36 |
| AC026355.1 | FKTN | 0.540529204 | 6.45E-42 |
| AL606489.1 | FMN1 | 0.529310713 | 5.81E-40 |
| AC026355.1 | FMR1 | 0.501525898 | 2.01E-35 |
| AC026355.1 | FNIP1 | 0.673618323 | 5.26E-72 |
| AC026355.1 | FOXN2 | 0.538879756 | 1.26E-41 |
| AC026355.1 | FRA10AC1 | 0.565961987 | 1.24E-46 |
| AC026355.1 | FRK | 0.524195335 | 4.28E-39 |
| AL606489.1 | FRK | 0.500639238 | 2.76E-35 |
| AC026355.1 | FRYL | 0.583305224 | 4.33E-50 |
| AL606489.1 | FRYL | 0.601529161 | 5.91E-54 |
| AL606489.1 | FUT4 | 0.526770218 | 1.57E-39 |
| AC026355.1 | G3BP1 | 0.526283822 | 1.90E-39 |
| AC026355.1 | GAB1 | 0.564384152 | 2.50E-46 |
| AC026355.1 | GABPB1 | 0.688313279 | 2.47E-76 |
| AC026355.1 | GAN | 0.559827585 | 1.86E-45 |
| AL606489.1 | GAN | 0.500920358 | 2.50E-35 |
| AL606489.1 | GCC2 | 0.554610071 | 1.77E-44 |
| AC026355.1 | GDAP2 | 0.733574628 | 1.85E-91 |
| AC026355.1 | GEN1 | 0.537178861 | 2.52E-41 |
| AC026355.1 | GIMAP5 | 0.539880779 | 8.40E-42 |
| AC026355.1 | GIT2 | 0.536667383 | 3.10E-41 |
| AC026355.1 | GNPDA2 | 0.596286336 | 8.11E-53 |
| AC026355.1 | GNRH1 | 0.57245433 | 6.67E-48 |
| AL606489.1 | GOLGA4 | 0.501229642 | 2.24E-35 |
| AL606489.1 | GOLGB1 | 0.501990231 | 1.70E-35 |
| AC026355.1 | GPATCH2 | 0.628246941 | 4.32E-60 |
| AC026355.1 | GPATCH2L | 0.729276058 | 6.82E-90 |
| AL606489.1 | GPATCH2L | 0.520401766 | 1.84E-38 |
| AC026355.1 | GPR174 | 0.564564019 | 2.31E-46 |
| AC026355.1 | GPR18 | 0.787406458 | 4.48E-114 |
| AC026355.1 | GPR82 | 0.778063465 | 1.07E-109 |
| AC026355.1 | GPRIN3 | 0.561026817 | 1.10E-45 |
| AC026355.1 | GSAP | 0.531190099 | 2.77E-40 |
| AC026355.1 | GTPBP10 | 0.501012565 | 2.42E-35 |
| AC026355.1 | GUCY1A2 | 0.601202792 | 6.97E-54 |
| AC026355.1 | GXYLT2 | 0.533513673 | 1.10E-40 |
| AC026355.1 | HAUS3 | 0.504757694 | 6.26E-36 |
| AC026355.1 | HCFC2 | 0.589456779 | 2.29E-51 |
| AC026355.1 | HECTD2 | 0.705835777 | 7.57E-82 |
| AC026355.1 | HELB | 0.691594067 | 2.45E-77 |
| AL606489.1 | HELB | 0.506907915 | 2.86E-36 |
| AC026355.1 | HELZ | 0.683885761 | 5.29E-75 |
| AC026355.1 | HERC1 | 0.58896445 | 2.90E-51 |
| AC026355.1 | HERC4 | 0.664234507 | 2.28E-69 |
| AC026355.1 | HIPK3 | 0.551564769 | 6.50E-44 |
| AC026355.1 | HIVEP1 | 0.582166014 | 7.41E-50 |
| AC026355.1 | HMBOX1 | 0.729029549 | 8.37E-90 |
| AC026355.1 | HNRNPH1 | 0.516297494 | 8.76E-38 |
| AC026355.1 | HOOK3 | 0.544552936 | 1.23E-42 |
| LINC01843 | HSD17B2 | 0.502857113 | 1.25E-35 |
| AL606489.1 | IBTK | 0.504513076 | 6.84E-36 |
| AC026355.1 | ICE2 | 0.604070736 | 1.63E-54 |
| AC026355.1 | IGIP | 0.610189453 | 7.03E-56 |
| AC026355.1 | IKZF2 | 0.700737978 | 3.35E-80 |
| AC026355.1 | IL15 | 0.546685591 | 5.07E-43 |
| LINC01843 | IL1R2 | 0.544791291 | 1.11E-42 |
| AC026355.1 | IL6ST | 0.557959981 | 4.19E-45 |
| AC026355.1 | INO80D | 0.720341253 | 9.95E-87 |
| AL606489.1 | INO80D | 0.558547274 | 3.24E-45 |
| AC026355.1 | INSYN2 | 0.644891214 | 3.16E-64 |
| AC026355.1 | INTS6 | 0.730102658 | 3.43E-90 |
| AL606489.1 | INTS6 | 0.554390489 | 1.95E-44 |
| AC026355.1 | INTS6L | 0.532425937 | 1.69E-40 |
| AC026355.1 | INTU | 0.764501056 | 1.03E-103 |
| AC026355.1 | INVS | 0.562284847 | 6.33E-46 |
| AL606489.1 | INVS | 0.529552388 | 5.29E-40 |
| AC026355.1 | IREB2 | 0.501145908 | 2.31E-35 |
| AC026355.1 | ITSN2 | 0.50556802 | 4.66E-36 |
| AC026355.1 | JAK2 | 0.503702144 | 9.18E-36 |
| AC026355.1 | JMJD1C | 0.595562386 | 1.16E-52 |
| AL606489.1 | JMJD1C | 0.521115944 | 1.40E-38 |
| AC026355.1 | JMY | 0.61946054 | 5.23E-58 |
| AC026355.1 | KANSL1 | 0.565273757 | 1.69E-46 |
| AC026355.1 | KANSL1L | 0.631480303 | 7.11E-61 |
| AC026355.1 | KATNAL1 | 0.541662342 | 4.05E-42 |
| AC026355.1 | KCNAB1 | 0.634667544 | 1.17E-61 |
| AC026355.1 | KCNRG | 0.530349534 | 3.86E-40 |
| AC026355.1 | KDM4C | 0.622401012 | 1.07E-58 |
| AC026355.1 | KDM7A | 0.659467334 | 4.57E-68 |
| AC026355.1 | KIAA1109 | 0.680093152 | 7.00E-74 |
| AC026355.1 | KIAA1551 | 0.567422896 | 6.47E-47 |
| AL606489.1 | KIAA1551 | 0.529870836 | 4.66E-40 |
| AC026355.1 | KIAA2026 | 0.674784251 | 2.44E-72 |
| AC026355.1 | KLC1 | 0.530980633 | 3.01E-40 |
| AC026355.1 | KLF12 | 0.579631248 | 2.43E-49 |
| AC026355.1 | KLF8 | 0.523177664 | 6.35E-39 |
| AC026355.1 | KLHDC1 | 0.633616048 | 2.13E-61 |
| AC026355.1 | KLHL15 | 0.535082303 | 5.86E-41 |
| AC026355.1 | KLHL24 | 0.515105581 | 1.37E-37 |
| AC026355.1 | KLHL28 | 0.517996522 | 4.61E-38 |
| AC026355.1 | KMT2A | 0.552384385 | 4.59E-44 |
| AL606489.1 | KMT2A | 0.516718508 | 7.47E-38 |
| AC026355.1 | KMT2C | 0.712463328 | 4.86E-84 |
| AC026355.1 | KMT2E | 0.537286749 | 2.41E-41 |
| AC026355.1 | KPNA5 | 0.589508303 | 2.23E-51 |
| AC026355.1 | KRIT1 | 0.521762173 | 1.09E-38 |
| AC026355.1 | LARP4 | 0.569508516 | 2.53E-47 |
| AC026355.1 | LATS1 | 0.585936072 | 1.24E-50 |
| AC026355.1 | LCOR | 0.632705891 | 3.57E-61 |
| AL606489.1 | LCOR | 0.544667439 | 1.17E-42 |
| AC026355.1 | LCORL | 0.679689842 | 9.19E-74 |
| AC026355.1 | LIN54 | 0.504968261 | 5.80E-36 |
| AC026355.1 | LIN7C | 0.614489579 | 7.39E-57 |
| AC026355.1 | LNPEP | 0.695455018 | 1.56E-78 |
| AC026355.1 | LPP | 0.759809862 | 9.81E-102 |
| AL606489.1 | LRCH1 | 0.537829989 | 1.93E-41 |
| AC026355.1 | LRCH3 | 0.60371309 | 1.96E-54 |
| AL606489.1 | LRCH3 | 0.556050363 | 9.55E-45 |
| AC026355.1 | LRIG2 | 0.637760113 | 2.01E-62 |
| AC026355.1 | LRP2BP | 0.678919308 | 1.54E-73 |
| AC026355.1 | LRP6 | 0.535525235 | 4.90E-41 |
| AC026355.1 | LRRFIP2 | 0.528821379 | 7.05E-40 |
| AC026355.1 | LRRN3 | 0.624507988 | 3.39E-59 |
| AC026355.1 | LSMEM1 | 0.551384415 | 7.02E-44 |
| AC026355.1 | LTN1 | 0.616285377 | 2.85E-57 |
| AC026355.1 | LUC7L2 | 0.572075917 | 7.92E-48 |
| AC026355.1 | LYRM7 | 0.607364241 | 3.03E-55 |
| AC026355.1 | LYSMD3 | 0.564456688 | 2.43E-46 |
| AC026355.1 | LYST | 0.717022652 | 1.39E-85 |
| AC026355.1 | MACF1 | 0.580327341 | 1.76E-49 |
| AC026355.1 | MAK | 0.660353834 | 2.63E-68 |
| AC026355.1 | MAN1A2 | 0.502972075 | 1.19E-35 |
| AC026355.1 | MAP3K1 | 0.539206645 | 1.11E-41 |
| AC026355.1 | MAP3K2 | 0.661804272 | 1.06E-68 |
| AL606489.1 | MAP3K2 | 0.547801859 | 3.18E-43 |
| AC026355.1 | MBD5 | 0.640927279 | 3.22E-63 |
| AC026355.1 | MBLAC2 | 0.537931803 | 1.86E-41 |
| AC026355.1 | MBNL1 | 0.567216995 | 7.09E-47 |
| AC026355.1 | MBNL2 | 0.527521776 | 1.17E-39 |
| AC026355.1 | MBNL3 | 0.515623706 | 1.13E-37 |
| AC026355.1 | MBTD1 | 0.522950498 | 6.93E-39 |
| AC026355.1 | MCTP1 | 0.626959453 | 8.81E-60 |
| AC026355.1 | MCTP2 | 0.552658983 | 4.08E-44 |
| AC026355.1 | MDM4 | 0.75069027 | 5.11E-98 |
| AC026355.1 | MED13 | 0.550029209 | 1.25E-43 |
| AC026355.1 | MEF2A | 0.532955836 | 1.37E-40 |
| AC026355.1 | MEMO1 | 0.804728312 | 8.34E-123 |
| AC026355.1 | METTL15 | 0.590651562 | 1.28E-51 |
| AC026355.1 | MFSD4B | 0.638492166 | 1.32E-62 |
| AC026355.1 | MFSD8 | 0.527633825 | 1.12E-39 |
| AC026355.1 | MGA | 0.598105515 | 3.29E-53 |
| AC026355.1 | MICU3 | 0.616942666 | 2.01E-57 |
| AC026355.1 | MIER1 | 0.554034615 | 2.27E-44 |
| AC026355.1 | MINDY2 | 0.639377932 | 7.90E-63 |
| AC026355.1 | MKLN1 | 0.776165798 | 7.77E-109 |
| AL606489.1 | MKLN1 | 0.513759246 | 2.27E-37 |
| AC026355.1 | MLH3 | 0.633224488 | 2.66E-61 |
| AC026355.1 | MLLT10 | 0.518272335 | 4.15E-38 |
| AC026355.1 | MLYCD | 0.531951142 | 2.05E-40 |
| AC026355.1 | MMS22L | 0.552181912 | 5.00E-44 |
| AC026355.1 | MOB1B | 0.624450827 | 3.50E-59 |
| AC026355.1 | MON2 | 0.691713715 | 2.25E-77 |
| AL606489.1 | MON2 | 0.53104772 | 2.93E-40 |
| AC026355.1 | MORC3 | 0.5504484 | 1.04E-43 |
| AC026355.1 | MOSMO | 0.601200942 | 6.98E-54 |
| AC026355.1 | MPHOSPH9 | 0.573215885 | 4.71E-48 |
| AC026355.1 | MPP6 | 0.506729922 | 3.05E-36 |
| AC026355.1 | MRTFB | 0.714012663 | 1.46E-84 |
| AC026355.1 | MTF2 | 0.527194353 | 1.33E-39 |
| AC026355.1 | MTHFD2L | 0.663797896 | 3.00E-69 |
| AC026355.1 | MTMR9 | 0.602736682 | 3.21E-54 |
| AC026355.1 | MTX3 | 0.70049482 | 4.00E-80 |
| AL606489.1 | MXD1 | 0.574773704 | 2.31E-48 |
| AC026355.1 | MYCBP2 | 0.602191185 | 4.23E-54 |
| AC026355.1 | MYO9A | 0.646946448 | 9.35E-65 |
| AC026355.1 | MYSM1 | 0.692664721 | 1.15E-77 |
| AC026355.1 | N4BP2 | 0.567942999 | 5.12E-47 |
| AC026355.1 | N4BP2L2 | 0.745454729 | 5.89E-96 |
| AC026355.1 | NAA25 | 0.531797318 | 2.17E-40 |
| AC026355.1 | NABP1 | 0.593160059 | 3.78E-52 |
| AC026355.1 | NAPB | 0.547940123 | 3.00E-43 |
| AC026355.1 | NAPEPLD | 0.520279747 | 1.93E-38 |
| AC026355.1 | NBEAL1 | 0.665713556 | 8.88E-70 |
| AL606489.1 | NBEAL1 | 0.531836917 | 2.14E-40 |
| AC026355.1 | NEK7 | 0.510830439 | 6.76E-37 |
| AC026355.1 | NEMF | 0.504501842 | 6.87E-36 |
| AC026355.1 | NEMP2 | 0.523912619 | 4.78E-39 |
| AL606489.1 | NEMP2 | 0.514471517 | 1.74E-37 |
| AC026355.1 | NF1 | 0.668857875 | 1.18E-70 |
| AC026355.1 | NFAT5 | 0.692934603 | 9.47E-78 |
| AC026355.1 | NFIA | 0.567262431 | 6.95E-47 |
| AC026355.1 | NFIB | 0.612854679 | 1.75E-56 |
| AC026355.1 | NHLRC2 | 0.631742068 | 6.13E-61 |
| AC026355.1 | NIN | 0.505634762 | 4.55E-36 |
| AC026355.1 | NKTR | 0.71143878 | 1.07E-83 |
| AC026355.1 | NPAT | 0.597761627 | 3.90E-53 |
| AC026355.1 | NPHP3 | 0.52659335 | 1.69E-39 |
| AC026355.1 | NR2C2 | 0.56458865 | 2.29E-46 |
| AC026355.1 | NRIP1 | 0.559406363 | 2.23E-45 |
| AC026355.1 | NSD1 | 0.527595344 | 1.14E-39 |
| AC026355.1 | NSUN3 | 0.585527986 | 1.51E-50 |
| AC026355.1 | NUDT13 | 0.669992487 | 5.64E-71 |
| AC026355.1 | NUFIP2 | 0.693448925 | 6.57E-78 |
| AC026355.1 | OCLM | 0.750542985 | 5.85E-98 |
| AC026355.1 | ODF2L | 0.645281072 | 2.51E-64 |
| AC026355.1 | OGT | 0.525233109 | 2.86E-39 |
| AC026355.1 | OMG | 0.547620721 | 3.43E-43 |
| AC026355.1 | OSBPL8 | 0.60749166 | 2.84E-55 |
| AC026355.1 | OTUD4 | 0.568205273 | 4.55E-47 |
| AC026355.1 | OTULIN | 0.50967729 | 1.03E-36 |
| AC026355.1 | OXNAD1 | 0.670861143 | 3.21E-71 |
| AC026355.1 | PACRGL | 0.53901831 | 1.19E-41 |
| AC026355.1 | PAN3 | 0.585078713 | 1.87E-50 |
| AL606489.1 | PAN3 | 0.51445359 | 1.75E-37 |
| AC026355.1 | PANK3 | 0.684784575 | 2.85E-75 |
| AC026355.1 | PAPOLG | 0.591503952 | 8.48E-52 |
| AC026355.1 | PARP8 | 0.677030914 | 5.48E-73 |
| AC026355.1 | PATJ | 0.514082424 | 2.01E-37 |
| AC026355.1 | PAXBP1 | 0.593584646 | 3.07E-52 |
| AC026355.1 | PBRM1 | 0.530503425 | 3.63E-40 |
| AC026355.1 | PCBD2 | 0.573026591 | 5.13E-48 |
| AC026355.1 | PCDHB12 | 0.626296645 | 1.27E-59 |
| AC026355.1 | PCDHB13 | 0.530265088 | 3.99E-40 |
| AC026355.1 | PCF11 | 0.528887134 | 6.87E-40 |
| AC026355.1 | PCMTD1 | 0.559493632 | 2.15E-45 |
| AC026355.1 | PCNX1 | 0.663880816 | 2.85E-69 |
| AC026355.1 | PCNX4 | 0.561890487 | 7.53E-46 |
| AL606489.1 | PCSK7 | 0.54934451 | 1.66E-43 |
| AC026355.1 | PDE5A | 0.640204039 | 4.90E-63 |
| AC026355.1 | PDK1 | 0.505770376 | 4.33E-36 |
| AC026355.1 | PDS5B | 0.549013037 | 1.91E-43 |
| AL606489.1 | PDZD8 | 0.578400735 | 4.32E-49 |
| AC026355.1 | PGAP1 | 0.639066148 | 9.46E-63 |
| AC026355.1 | PGGT1B | 0.702900637 | 6.78E-81 |
| AC026355.1 | PHC3 | 0.712403274 | 5.09E-84 |
| AL606489.1 | PHC3 | 0.554627484 | 1.76E-44 |
| AC026355.1 | PHF20L1 | 0.63111954 | 8.70E-61 |
| AC026355.1 | PHF3 | 0.501416399 | 2.09E-35 |
| AC026355.1 | PHIP | 0.668187906 | 1.81E-70 |
| AC026355.1 | PHOSPHO2 | 0.654171786 | 1.20E-66 |
| AC026355.1 | PIAS1 | 0.544774268 | 1.12E-42 |
| AC026355.1 | PIAS2 | 0.688297664 | 2.49E-76 |
| AC026355.1 | PIGB | 0.591845867 | 7.18E-52 |
| AC026355.1 | PIK3C2A | 0.626288036 | 1.28E-59 |
| AL606489.1 | PIK3C2B | 0.501306021 | 2.18E-35 |
| AC026355.1 | PIK3CA | 0.562778653 | 5.09E-46 |
| AL606489.1 | PIK3CA | 0.512829983 | 3.21E-37 |
| AL606489.1 | PIK3CB | 0.514309622 | 1.85E-37 |
| AC026355.1 | PIK3R1 | 0.535891243 | 4.23E-41 |
| AC026355.1 | PIKFYVE | 0.644227524 | 4.67E-64 |
| AC026355.1 | PLEKHA3 | 0.725256796 | 1.87E-88 |
| AC026355.1 | PLEKHA8 | 0.591570727 | 8.21E-52 |
| AC026355.1 | PLEKHM3 | 0.568967463 | 3.23E-47 |
| AC026355.1 | PLXDC2 | 0.522771867 | 7.42E-39 |
| AC026355.1 | PMM2 | 0.536180487 | 3.77E-41 |
| AL606489.1 | PMM2 | 0.550885662 | 8.67E-44 |
| AC026355.1 | PNISR | 0.523474943 | 5.66E-39 |
| LINC01843 | POLD4 | 0.504945162 | 5.85E-36 |
| AC026355.1 | POLI | 0.556668081 | 7.32E-45 |
| AC026355.1 | POLK | 0.646667964 | 1.10E-64 |
| AC026355.1 | POU2F1 | 0.618398572 | 9.25E-58 |
| AC026355.1 | PPIP5K2 | 0.683340441 | 7.68E-75 |
| AC026355.1 | PPM1A | 0.56593236 | 1.26E-46 |
| AC026355.1 | PPP1R12A | 0.524798702 | 3.39E-39 |
| AL606489.1 | PPP1R15B | 0.501841326 | 1.80E-35 |
| AC026355.1 | PPP2R3A | 0.500166266 | 3.27E-35 |
| AC026355.1 | PPWD1 | 0.590406676 | 1.44E-51 |
| AC026355.1 | PREX2 | 0.592000425 | 6.66E-52 |
| AC026355.1 | PRKCE | 0.554611382 | 1.77E-44 |
| AC026355.1 | PRPF4B | 0.507819292 | 2.05E-36 |
| AC026355.1 | PSMG4 | 0.516615776 | 7.77E-38 |
| AC026355.1 | PTAR1 | 0.664791887 | 1.60E-69 |
| AC026355.1 | PTBP2 | 0.57907799 | 3.15E-49 |
| AL606489.1 | PTBP3 | 0.540056327 | 7.82E-42 |
| AC026355.1 | PTEN | 0.630720893 | 1.09E-60 |
| AC026355.1 | PTPDC1 | 0.578627522 | 3.89E-49 |
| AC026355.1 | PTPN4 | 0.569798711 | 2.22E-47 |
| AC026355.1 | PURA | 0.778120029 | 1.00E-109 |
| AC026355.1 | PURB | 0.566992867 | 7.84E-47 |
| AC026355.1 | PUS10 | 0.739441964 | 1.19E-93 |
| AC026355.1 | PUS7L | 0.742355929 | 9.27E-95 |
| AC026355.1 | PWWP2A | 0.551951545 | 5.52E-44 |
| AC026355.1 | QKI | 0.5993426 | 1.77E-53 |
| AC026355.1 | QSER1 | 0.503308326 | 1.06E-35 |
| AC026355.1 | RAD51B | 0.520392419 | 1.85E-38 |
| AC026355.1 | RALGPS2 | 0.550570258 | 9.91E-44 |
| AC026355.1 | RANBP17 | 0.632452145 | 4.11E-61 |
| AC026355.1 | RAPGEF6 | 0.628884931 | 3.03E-60 |
| AC026355.1 | RAPH1 | 0.580502342 | 1.62E-49 |
| AC026355.1 | RASA1 | 0.601316075 | 6.58E-54 |
| AC026355.1 | RASA2 | 0.645294787 | 2.49E-64 |
| AL606489.1 | RASA2 | 0.514549976 | 1.69E-37 |
| AC026355.1 | RASAL2 | 0.533529068 | 1.09E-40 |
| AC026355.1 | RASGEF1B | 0.515642865 | 1.12E-37 |
| AC026355.1 | RBAK | 0.531149203 | 2.81E-40 |
| AC026355.1 | RBM12B | 0.664535157 | 1.88E-69 |
| AC026355.1 | RBM14-RBM4 | 0.718387218 | 4.71E-86 |
| AC026355.1 | RBM15 | 0.598367756 | 2.88E-53 |
| AL606489.1 | RBM15 | 0.500884617 | 2.53E-35 |
| AC026355.1 | RBM25 | 0.504614038 | 6.60E-36 |
| AC026355.1 | RBM26 | 0.58296417 | 5.09E-50 |
| AC026355.1 | RBM27 | 0.585966797 | 1.22E-50 |
| AC026355.1 | RBM33 | 0.57616651 | 1.22E-48 |
| AC026355.1 | RBM39 | 0.56878838 | 3.50E-47 |
| AC026355.1 | RBM4 | 0.507785611 | 2.07E-36 |
| AC026355.1 | RBM41 | 0.566024583 | 1.21E-46 |
| AC026355.1 | RBMS1 | 0.586035414 | 1.18E-50 |
| AC026355.1 | RBMS3 | 0.536844181 | 2.88E-41 |
| AC026355.1 | RC3H1 | 0.746237248 | 2.92E-96 |
| AL606489.1 | RC3H1 | 0.529911821 | 4.59E-40 |
| AC026355.1 | RC3H2 | 0.577430195 | 6.78E-49 |
| AC026355.1 | RCOR3 | 0.57928745 | 2.86E-49 |
| LINC01843 | REG4 | 0.542040798 | 3.47E-42 |
| AC026355.1 | REL | 0.76214767 | 1.03E-102 |
| AC026355.1 | RELCH | 0.61008068 | 7.44E-56 |
| AC026355.1 | REST | 0.603180061 | 2.57E-54 |
| AC026355.1 | REV3L | 0.683453275 | 7.11E-75 |
| AC026355.1 | RFX3 | 0.589018433 | 2.83E-51 |
| AC026355.1 | RFX7 | 0.630651337 | 1.13E-60 |
| AC026355.1 | RIC1 | 0.67966686 | 9.33E-74 |
| AC026355.1 | RICTOR | 0.600510695 | 9.87E-54 |
| AC026355.1 | RIF1 | 0.616175898 | 3.03E-57 |
| AL606489.1 | RIF1 | 0.531935504 | 2.06E-40 |
| AC026355.1 | RLF | 0.508691639 | 1.49E-36 |
| AC026355.1 | RLIM | 0.659991302 | 3.30E-68 |
| AC026355.1 | RNF152 | 0.555927273 | 1.01E-44 |
| AC026355.1 | RNF169 | 0.643659891 | 6.52E-64 |
| AC026355.1 | RNF217 | 0.634764775 | 1.11E-61 |
| AC026355.1 | RNPC3 | 0.596719319 | 6.55E-53 |
| AL606489.1 | ROCK2 | 0.533961738 | 9.18E-41 |
| AC026355.1 | RORA | 0.720033405 | 1.27E-86 |
| AC026355.1 | RPAP2 | 0.653867297 | 1.45E-66 |
| AC026355.1 | RPS6KA3 | 0.504983621 | 5.77E-36 |
| AC026355.1 | RSBN1 | 0.516578409 | 7.88E-38 |
| AC026355.1 | RSBN1L | 0.530570891 | 3.54E-40 |
| AC026355.1 | RTTN | 0.520644779 | 1.68E-38 |
| AC026355.1 | RUFY2 | 0.651156395 | 7.50E-66 |
| AC026355.1 | RUFY3 | 0.662573401 | 6.52E-69 |
| AC026355.1 | RUNX1 | 0.513516875 | 2.49E-37 |
| AC026355.1 | RUNX1T1 | 0.601357261 | 6.45E-54 |
| AC026355.1 | S100PBP | 0.630967293 | 9.48E-61 |
| AC026355.1 | SAMD12 | 0.602730659 | 3.22E-54 |
| AC026355.1 | SAMD8 | 0.580554118 | 1.58E-49 |
| AC026355.1 | SARNP | 0.637311406 | 2.60E-62 |
| AC026355.1 | SCAF11 | 0.599111728 | 1.99E-53 |
| AL606489.1 | SCAF11 | 0.519763173 | 2.35E-38 |
| AC026355.1 | SCAF8 | 0.562180912 | 6.63E-46 |
| AC026355.1 | SCAI | 0.628574972 | 3.60E-60 |
| AC026355.1 | SCAPER | 0.520661196 | 1.67E-38 |
| AC026355.1 | SCLT1 | 0.61823232 | 1.01E-57 |
| AC026355.1 | SEC24A | 0.543437357 | 1.95E-42 |
| AC026355.1 | SECISBP2 | 0.531524209 | 2.42E-40 |
| AC026355.1 | SENP5 | 0.511726295 | 4.85E-37 |
| AC026355.1 | SENP6 | 0.514562204 | 1.68E-37 |
| AC026355.1 | SENP7 | 0.591028831 | 1.07E-51 |
| AC026355.1 | SEPSECS | 0.510012175 | 9.14E-37 |
| AC026355.1 | SESN3 | 0.535810366 | 4.37E-41 |
| AC026355.1 | SETD2 | 0.578377399 | 4.36E-49 |
| AC026355.1 | SETX | 0.616261912 | 2.89E-57 |
| AL606489.1 | SETX | 0.507506971 | 2.30E-36 |
| AC026355.1 | SFT2D2 | 0.680706238 | 4.62E-74 |
| AC026355.1 | SGIP1 | 0.61940862 | 5.38E-58 |
| AC026355.1 | SHPRH | 0.742687472 | 6.91E-95 |
| AL606489.1 | SHPRH | 0.511999011 | 4.38E-37 |
| AC026355.1 | SIAH1 | 0.511714566 | 4.87E-37 |
| AC026355.1 | SIKE1 | 0.517612282 | 5.33E-38 |
| AC026355.1 | SKIL | 0.540620373 | 6.21E-42 |
| AC026355.1 | SLC10A7 | 0.526960367 | 1.46E-39 |
| AC026355.1 | SLC25A36 | 0.617932402 | 1.19E-57 |
| AC026355.1 | SLC25A46 | 0.588830297 | 3.09E-51 |
| AC026355.1 | SLC30A4 | 0.657553675 | 1.50E-67 |
| AL606489.1 | SLC30A4 | 0.528620844 | 7.63E-40 |
| AC026355.1 | SLC30A7 | 0.574944684 | 2.13E-48 |
| AL606489.1 | SLC30A7 | 0.510738238 | 6.99E-37 |
| AC026355.1 | SLC35F5 | 0.510353104 | 8.06E-37 |
| AC026355.1 | SLC38A6 | 0.565365502 | 1.62E-46 |
| AC026355.1 | SLC4A7 | 0.689525365 | 1.05E-76 |
| AC026355.1 | SLC5A3 | 0.579011996 | 3.25E-49 |
| AC026355.1 | SLC8A1 | 0.559153556 | 2.49E-45 |
| AC026355.1 | SLF1 | 0.503507783 | 9.85E-36 |
| AC026355.1 | SLF2 | 0.645945177 | 1.69E-64 |
| AC026355.1 | SLFN12L | 0.626307253 | 1.26E-59 |
| AL606489.1 | SLMAP | 0.636246089 | 4.78E-62 |
| AC026355.1 | SMAD2 | 0.601884252 | 4.94E-54 |
| AC026355.1 | SMAD4 | 0.57713162 | 7.78E-49 |
| AC026355.1 | SMAD5 | 0.591595771 | 8.11E-52 |
| AL606489.1 | SMC5 | 0.520578953 | 1.72E-38 |
| AC026355.1 | SMCHD1 | 0.597666373 | 4.09E-53 |
| AC026355.1 | SMG1 | 0.608716627 | 1.51E-55 |
| AL606489.1 | SMG1 | 0.533478093 | 1.11E-40 |
| AC026355.1 | SMIM8 | 0.648851629 | 3.00E-65 |
| AC026355.1 | SMYD3 | 0.57286805 | 5.52E-48 |
| AC026355.1 | SNAPC3 | 0.544735257 | 1.14E-42 |
| AC026355.1 | SNTB2 | 0.557590858 | 4.91E-45 |
| AC026355.1 | SNX13 | 0.649547683 | 1.98E-65 |
| AC026355.1 | SOCS4 | 0.594662565 | 1.81E-52 |
| AC026355.1 | SOCS5 | 0.564440272 | 2.44E-46 |
| AC026355.1 | SOS1 | 0.554478338 | 1.88E-44 |
| AC026355.1 | SOS2 | 0.501690977 | 1.90E-35 |
| AL606489.1 | SP1 | 0.542687233 | 2.66E-42 |
| AC026355.1 | SP3 | 0.519657343 | 2.45E-38 |
| AL606489.1 | SPATA5 | 0.547969645 | 2.96E-43 |
| AC026355.1 | SPATA6L | 0.597080749 | 5.47E-53 |
| AC026355.1 | SPG11 | 0.56166687 | 8.30E-46 |
| AL606489.1 | SPG11 | 0.507704953 | 2.14E-36 |
| AC026355.1 | SPICE1 | 0.568146411 | 4.68E-47 |
| AC026355.1 | SPOPL | 0.518532783 | 3.76E-38 |
| AC026355.1 | SREK1 | 0.727408413 | 3.20E-89 |
| AC026355.1 | SRFBP1 | 0.506832974 | 2.94E-36 |
| AC026355.1 | SRSF10 | 0.538319324 | 1.59E-41 |
| AC026355.1 | SSBP2 | 0.538205294 | 1.66E-41 |
| AC026355.1 | SSH2 | 0.572374149 | 6.91E-48 |
| AC026355.1 | ST7L | 0.621594609 | 1.66E-58 |
| AC026355.1 | STEAP2 | 0.511358276 | 5.56E-37 |
| AC026355.1 | STRADA | 0.555980691 | 9.85E-45 |
| AC026355.1 | STRN | 0.5407006 | 6.01E-42 |
| AC026355.1 | STX17 | 0.612101993 | 2.59E-56 |
| AL606489.1 | STX17 | 0.52165389 | 1.14E-38 |
| AC026355.1 | STXBP4 | 0.569070956 | 3.09E-47 |
| AC026355.1 | STXBP5 | 0.641897107 | 1.83E-63 |
| AC026355.1 | STYX | 0.533379602 | 1.16E-40 |
| AC026355.1 | SUGT1 | 0.590311685 | 1.51E-51 |
| AC026355.1 | SUPT20H | 0.591740796 | 7.55E-52 |
| AC026355.1 | SWT1 | 0.516673225 | 7.60E-38 |
| AC026355.1 | SYNJ1 | 0.521827761 | 1.07E-38 |
| AC026355.1 | SYNPO2 | 0.515405144 | 1.23E-37 |
| AC026355.1 | TAF1D | 0.514713589 | 1.59E-37 |
| AC026355.1 | TAMM41 | 0.624623928 | 3.18E-59 |
| AC026355.1 | TANC2 | 0.527880844 | 1.02E-39 |
| AC026355.1 | TAOK1 | 0.671227982 | 2.52E-71 |
| AC026355.1 | TARBP1 | 0.505789087 | 4.30E-36 |
| AC026355.1 | TAS2R14 | 0.746708864 | 1.91E-96 |
| AC026355.1 | TAS2R20 | 0.723844368 | 5.92E-88 |
| AC026355.1 | TBC1D12 | 0.595760072 | 1.05E-52 |
| AC026355.1 | TBC1D19 | 0.608770703 | 1.47E-55 |
| AC026355.1 | TBC1D32 | 0.616717879 | 2.27E-57 |
| AC026355.1 | TBCEL | 0.57172588 | 9.29E-48 |
| AC026355.1 | TBCK | 0.636129001 | 5.11E-62 |
| AC026355.1 | TBL1XR1 | 0.644823107 | 3.29E-64 |
| AC026355.1 | TCERG1 | 0.553403991 | 2.97E-44 |
| AC026355.1 | TCF4 | 0.54699212 | 4.46E-43 |
| AC026355.1 | TDRD3 | 0.536903723 | 2.81E-41 |
| AC026355.1 | TENT2 | 0.702333609 | 1.03E-80 |
| AC026355.1 | TET2 | 0.736193317 | 1.98E-92 |
| AC026355.1 | THAP6 | 0.657433071 | 1.62E-67 |
| AC026355.1 | TIA1 | 0.507549131 | 2.26E-36 |
| AL606489.1 | TM9SF3 | 0.521464607 | 1.23E-38 |
| AC026355.1 | TMEM161B | 0.724258457 | 4.23E-88 |
| AC026355.1 | TMEM168 | 0.584517213 | 2.44E-50 |
| AC026355.1 | TMEM170B | 0.559880779 | 1.81E-45 |
| AC026355.1 | TMEM33 | 0.500007707 | 3.46E-35 |
| AL606489.1 | TMEM87B | 0.508646029 | 1.51E-36 |
| AC026355.1 | TMPPE | 0.609910175 | 8.12E-56 |
| AL606489.1 | TMPPE | 0.595814206 | 1.02E-52 |
| AC026355.1 | TNPO1 | 0.57681757 | 9.00E-49 |
| AC026355.1 | TNRC6A | 0.560468439 | 1.40E-45 |
| AC026355.1 | TNRC6B | 0.665833835 | 8.22E-70 |
| AL606489.1 | TNRC6B | 0.525859095 | 2.25E-39 |
| AC026355.1 | TOR1AIP2 | 0.661305563 | 1.45E-68 |
| AC026355.1 | TRA2B | 0.596984159 | 5.74E-53 |
| AL606489.1 | TRA2B | 0.507637327 | 2.19E-36 |
| AC026355.1 | TRAF6 | 0.585065192 | 1.88E-50 |
| AL606489.1 | TRAF6 | 0.506014476 | 3.96E-36 |
| AC026355.1 | TRIM13 | 0.712729829 | 3.96E-84 |
| LINC01843 | TRIM15 | 0.521573854 | 1.18E-38 |
| AC026355.1 | TRIM23 | 0.540302896 | 7.07E-42 |
| AC026355.1 | TRIM33 | 0.568028049 | 4.93E-47 |
| AC026355.1 | TRIM38 | 0.500025191 | 3.44E-35 |
| AC026355.1 | TRIP11 | 0.57190355 | 8.57E-48 |
| AC026355.1 | TRMT13 | 0.568457831 | 4.07E-47 |
| AC026355.1 | TRNT1 | 0.530930993 | 3.07E-40 |
| AC026355.1 | TROVE2 | 0.708651509 | 9.02E-83 |
| AC026355.1 | TRPM7 | 0.646421712 | 1.28E-64 |
| AL606489.1 | TRPM7 | 0.524549407 | 3.73E-39 |
| AC026355.1 | TRPS1 | 0.636307222 | 4.62E-62 |
| AC026355.1 | TSSK4 | 0.633780362 | 1.94E-61 |
| AC026355.1 | TTBK2 | 0.682411837 | 1.45E-74 |
| AC026355.1 | TTC14 | 0.596368497 | 7.79E-53 |
| AC026355.1 | TTC3 | 0.528104257 | 9.34E-40 |
| AC026355.1 | TTC6 | 0.626865375 | 9.28E-60 |
| AC026355.1 | TUBE1 | 0.525221372 | 2.88E-39 |
| AC026355.1 | TULP4 | 0.620342101 | 3.26E-58 |
| AC026355.1 | TUT4 | 0.626432285 | 1.18E-59 |
| AC026355.1 | TUT7 | 0.502972259 | 1.19E-35 |
| AL606489.1 | TUT7 | 0.593382106 | 3.39E-52 |
| AC026355.1 | TVP23C | 0.719673319 | 1.70E-86 |
| AL606489.1 | TVP23C | 0.548288681 | 2.59E-43 |
| AL606489.1 | TXK | 0.504373376 | 7.20E-36 |
| AC026355.1 | TYW5 | 0.672680174 | 9.76E-72 |
| AL606489.1 | TYW5 | 0.528190934 | 9.03E-40 |
| AC026355.1 | UBA5 | 0.539858748 | 8.48E-42 |
| AC026355.1 | UBA6 | 0.526898364 | 1.50E-39 |
| AL606489.1 | UBA6 | 0.530582058 | 3.52E-40 |
| AC026355.1 | UBE2V1 | 0.672673058 | 9.80E-72 |
| AL606489.1 | UBE2V1 | 0.515586346 | 1.14E-37 |
| AC026355.1 | UBE3A | 0.543194589 | 2.16E-42 |
| AC026355.1 | UBN2 | 0.78839208 | 1.50E-114 |
| AC026355.1 | UBR1 | 0.52084475 | 1.56E-38 |
| AL606489.1 | UBR1 | 0.504488252 | 6.91E-36 |
| AC026355.1 | UBXN7 | 0.52880683 | 7.09E-40 |
| AC026355.1 | UCHL5 | 0.532988819 | 1.35E-40 |
| AC026355.1 | UGGT2 | 0.582356029 | 6.78E-50 |
| AC026355.1 | UHMK1 | 0.503120917 | 1.13E-35 |
| AC026355.1 | UHRF1BP1L | 0.510400232 | 7.92E-37 |
| AC026355.1 | ULK4 | 0.62268342 | 9.17E-59 |
| AC026355.1 | USF3 | 0.67864323 | 1.86E-73 |
| AL606489.1 | USF3 | 0.50924086 | 1.22E-36 |
| AC026355.1 | USP15 | 0.584939936 | 1.99E-50 |
| AL606489.1 | USP15 | 0.507301038 | 2.48E-36 |
| AC026355.1 | USP24 | 0.526364875 | 1.84E-39 |
| AL606489.1 | USP24 | 0.512943163 | 3.08E-37 |
| AC026355.1 | USP3 | 0.535390308 | 5.18E-41 |
| AC026355.1 | USP34 | 0.705933386 | 7.04E-82 |
| AL606489.1 | USP34 | 0.545131671 | 9.68E-43 |
| AC026355.1 | USP37 | 0.562544935 | 5.64E-46 |
| AC026355.1 | USP45 | 0.743896489 | 2.37E-95 |
| AC026355.1 | USP53 | 0.611703141 | 3.19E-56 |
| AC026355.1 | UTRN | 0.559992273 | 1.73E-45 |
| AC026355.1 | VAMP4 | 0.578799147 | 3.59E-49 |
| AC026355.1 | VCPIP1 | 0.584264794 | 2.75E-50 |
| AC026355.1 | VMP1 | 0.674024833 | 4.03E-72 |
| AC026355.1 | VN1R1 | 0.526846944 | 1.53E-39 |
| AC026355.1 | VPS13A | 0.624680156 | 3.08E-59 |
| AL606489.1 | VPS13A | 0.526022597 | 2.11E-39 |
| AC026355.1 | VPS13B | 0.697586404 | 3.34E-79 |
| AL606489.1 | VPS13B | 0.538166021 | 1.69E-41 |
| AC026355.1 | VPS13C | 0.702658351 | 8.11E-81 |
| AC026355.1 | VPS13D | 0.570908734 | 1.35E-47 |
| AC026355.1 | VPS8 | 0.63016416 | 1.49E-60 |
| AL606489.1 | VPS8 | 0.529629806 | 5.13E-40 |
| AC026355.1 | WASHC4 | 0.598765577 | 2.37E-53 |
| AC026355.1 | WDFY2 | 0.523792864 | 5.01E-39 |
| AC026355.1 | WDFY3 | 0.692774287 | 1.06E-77 |
| AL606489.1 | WDFY3 | 0.528518087 | 7.94E-40 |
| AC026355.1 | WDR11 | 0.512252145 | 3.99E-37 |
| AC026355.1 | WDR26 | 0.578259424 | 4.61E-49 |
| AC026355.1 | WDR27 | 0.550616646 | 9.72E-44 |
| AC026355.1 | WDR36 | 0.538993672 | 1.21E-41 |
| AC026355.1 | WDR7 | 0.580436536 | 1.67E-49 |
| AC026355.1 | XIAP | 0.512853954 | 3.19E-37 |
| AC026355.1 | XPO4 | 0.616279442 | 2.86E-57 |
| AL606489.1 | XPO4 | 0.516169523 | 9.19E-38 |
| AC026355.1 | XRN1 | 0.623862144 | 4.82E-59 |
| AL606489.1 | XRN1 | 0.554181134 | 2.13E-44 |
| AC026355.1 | YAF2 | 0.724689096 | 2.98E-88 |
| AC026355.1 | YTHDC2 | 0.609922864 | 8.07E-56 |
| AC026355.1 | ZBED6 | 0.761851144 | 1.37E-102 |
| AL606489.1 | ZBED6 | 0.522774681 | 7.42E-39 |
| AC026355.1 | ZBTB11 | 0.528684485 | 7.44E-40 |
| AC026355.1 | ZBTB20 | 0.724274066 | 4.17E-88 |
| AL606489.1 | ZBTB20 | 0.516260842 | 8.88E-38 |
| AC026355.1 | ZBTB21 | 0.519604669 | 2.50E-38 |
| AC026355.1 | ZBTB25 | 0.647982997 | 5.04E-65 |
| AC026355.1 | ZBTB37 | 0.832838644 | 5.08E-139 |
| AC026355.1 | ZBTB38 | 0.501590161 | 1.97E-35 |
| AC026355.1 | ZBTB41 | 0.660719038 | 2.09E-68 |
| AC026355.1 | ZBTB43 | 0.515692947 | 1.10E-37 |
| AC026355.1 | ZBTB44 | 0.565373523 | 1.61E-46 |
| AC026355.1 | ZC3H11A | 0.637332505 | 2.57E-62 |
| AL606489.1 | ZC3H12C | 0.516570577 | 7.90E-38 |
| AC026355.1 | ZC3H6 | 0.652057797 | 4.35E-66 |
| AC026355.1 | ZDHHC17 | 0.68627577 | 1.02E-75 |
| AL606489.1 | ZDHHC20 | 0.522257834 | 9.05E-39 |
| AC026355.1 | ZDHHC21 | 0.715622752 | 4.16E-85 |
| AC026355.1 | ZEB1 | 0.516908096 | 6.96E-38 |
| AC026355.1 | ZEB2 | 0.671555265 | 2.04E-71 |
| AC026355.1 | ZFC3H1 | 0.653636683 | 1.67E-66 |
| AL606489.1 | ZFC3H1 | 0.512942726 | 3.08E-37 |
| AC026355.1 | ZFP14 | 0.519236411 | 2.88E-38 |
| AC026355.1 | ZFX | 0.599825878 | 1.39E-53 |
| AC026355.1 | ZFYVE16 | 0.709313609 | 5.45E-83 |
| AC026355.1 | ZGRF1 | 0.622846866 | 8.39E-59 |
| AC026355.1 | ZMAT3 | 0.543047325 | 2.29E-42 |
| AC026355.1 | ZMYM1 | 0.54302172 | 2.32E-42 |
| AC026355.1 | ZMYM2 | 0.666868771 | 4.24E-70 |
| AL606489.1 | ZMYM2 | 0.528442432 | 8.18E-40 |
| AC026355.1 | ZMYM6 | 0.634443229 | 1.33E-61 |
| AC026355.1 | ZNF10 | 0.531253991 | 2.70E-40 |
| AC026355.1 | ZNF107 | 0.600356502 | 1.07E-53 |
| AC026355.1 | ZNF117 | 0.575104935 | 1.98E-48 |
| AC026355.1 | ZNF121 | 0.621213357 | 2.03E-58 |
| AC026355.1 | ZNF124 | 0.640843952 | 3.38E-63 |
| AC026355.1 | ZNF138 | 0.540790649 | 5.79E-42 |
| AC026355.1 | ZNF141 | 0.698928958 | 1.26E-79 |
| AC026355.1 | ZNF148 | 0.65527019 | 6.13E-67 |
| AC026355.1 | ZNF154 | 0.639247205 | 8.52E-63 |
| AC026355.1 | ZNF160 | 0.500643876 | 2.76E-35 |
| AC026355.1 | ZNF17 | 0.5386185 | 1.40E-41 |
| AC026355.1 | ZNF207 | 0.741788504 | 1.53E-94 |
| AC026355.1 | ZNF235 | 0.692526291 | 1.27E-77 |
| AC026355.1 | ZNF236 | 0.507539647 | 2.27E-36 |
| AC026355.1 | ZNF248 | 0.588262814 | 4.07E-51 |
| AC026355.1 | ZNF25 | 0.588404035 | 3.80E-51 |
| AC026355.1 | ZNF254 | 0.632454084 | 4.11E-61 |
| AC026355.1 | ZNF26 | 0.631578807 | 6.72E-61 |
| AC026355.1 | ZNF264 | 0.645381036 | 2.37E-64 |
| AC026355.1 | ZNF267 | 0.564593048 | 2.28E-46 |
| AC026355.1 | ZNF280C | 0.503688828 | 9.22E-36 |
| AC026355.1 | ZNF280D | 0.682533217 | 1.33E-74 |
| AC026355.1 | ZNF283 | 0.693279433 | 7.41E-78 |
| AC026355.1 | ZNF292 | 0.63478703 | 1.10E-61 |
| AC026355.1 | ZNF326 | 0.586053701 | 1.17E-50 |
| AC026355.1 | ZNF33A | 0.624635716 | 3.16E-59 |
| AC026355.1 | ZNF354B | 0.506532791 | 3.28E-36 |
| AC026355.1 | ZNF37A | 0.534004157 | 9.02E-41 |
| AC026355.1 | ZNF407 | 0.672726595 | 9.46E-72 |
| AL606489.1 | ZNF407 | 0.507904744 | 1.99E-36 |
| AC026355.1 | ZNF417 | 0.622965819 | 7.86E-59 |
| AC026355.1 | ZNF431 | 0.582261132 | 7.09E-50 |
| AC026355.1 | ZNF433 | 0.554620868 | 1.77E-44 |
| AC026355.1 | ZNF441 | 0.528867644 | 6.92E-40 |
| AC026355.1 | ZNF449 | 0.599329076 | 1.79E-53 |
| AC026355.1 | ZNF451 | 0.721606244 | 3.61E-87 |
| AC026355.1 | ZNF460 | 0.706664541 | 4.06E-82 |
| AC026355.1 | ZNF484 | 0.586673556 | 8.72E-51 |
| AC026355.1 | ZNF493 | 0.713251597 | 2.64E-84 |
| AC026355.1 | ZNF506 | 0.687826972 | 3.46E-76 |
| AC026355.1 | ZNF507 | 0.583062 | 4.86E-50 |
| AC026355.1 | ZNF510 | 0.63421109 | 1.52E-61 |
| AC026355.1 | ZNF518A | 0.622095371 | 1.26E-58 |
| AL606489.1 | ZNF518A | 0.561251047 | 9.97E-46 |
| AC026355.1 | ZNF528 | 0.518442543 | 3.89E-38 |
| AC026355.1 | ZNF529 | 0.540988285 | 5.34E-42 |
| AC026355.1 | ZNF543 | 0.522156067 | 9.41E-39 |
| AC026355.1 | ZNF546 | 0.736596001 | 1.40E-92 |
| AC026355.1 | ZNF551 | 0.542144266 | 3.33E-42 |
| AC026355.1 | ZNF562 | 0.600348981 | 1.07E-53 |
| AC026355.1 | ZNF571 | 0.58176872 | 8.94E-50 |
| AC026355.1 | ZNF573 | 0.630419515 | 1.29E-60 |
| AC026355.1 | ZNF586 | 0.654447666 | 1.01E-66 |
| AC026355.1 | ZNF587 | 0.551429521 | 6.89E-44 |
| AC026355.1 | ZNF587B | 0.702835867 | 7.11E-81 |
| AC026355.1 | ZNF611 | 0.612478487 | 2.13E-56 |
| AC026355.1 | ZNF619 | 0.511989078 | 4.40E-37 |
| AC026355.1 | ZNF638 | 0.643165522 | 8.72E-64 |
| AC026355.1 | ZNF644 | 0.61217246 | 2.50E-56 |
| AC026355.1 | ZNF652 | 0.596071213 | 9.02E-53 |
| AC026355.1 | ZNF654 | 0.643340294 | 7.87E-64 |
| AC026355.1 | ZNF655 | 0.56392483 | 3.07E-46 |
| AC026355.1 | ZNF66 | 0.614794034 | 6.29E-57 |
| AC026355.1 | ZNF678 | 0.752558283 | 9.13E-99 |
| AC026355.1 | ZNF680 | 0.58601344 | 1.20E-50 |
| AC026355.1 | ZNF700 | 0.56795827 | 5.09E-47 |
| AC026355.1 | ZNF708 | 0.601758113 | 5.27E-54 |
| AC026355.1 | ZNF717 | 0.689239294 | 1.29E-76 |
| AC026355.1 | ZNF718 | 0.632879517 | 3.23E-61 |
| AC026355.1 | ZNF720 | 0.718741455 | 3.56E-86 |
| AC026355.1 | ZNF721 | 0.668799224 | 1.22E-70 |
| AC026355.1 | ZNF736 | 0.631064686 | 8.97E-61 |
| AC026355.1 | ZNF772 | 0.505954044 | 4.05E-36 |
| AC026355.1 | ZNF780A | 0.669370344 | 8.44E-71 |
| AC026355.1 | ZNF780B | 0.692070857 | 1.75E-77 |
| AC026355.1 | ZNF782 | 0.675659506 | 1.36E-72 |
| AL606489.1 | ZNF782 | 0.524929957 | 3.22E-39 |
| AC026355.1 | ZNF789 | 0.572268274 | 7.26E-48 |
| AC026355.1 | ZNF790 | 0.654540396 | 9.59E-67 |
| AC026355.1 | ZNF791 | 0.515951318 | 9.98E-38 |
| AC026355.1 | ZNF800 | 0.70154605 | 1.85E-80 |
| AC026355.1 | ZNF805 | 0.590643592 | 1.29E-51 |
| AC026355.1 | ZNF808 | 0.660244647 | 2.81E-68 |
| AC026355.1 | ZNF81 | 0.649137017 | 2.53E-65 |
| AL606489.1 | ZNF81 | 0.512153895 | 4.13E-37 |
| AC026355.1 | ZNF814 | 0.73845999 | 2.80E-93 |
| AC026355.1 | ZNF83 | 0.525252491 | 2.84E-39 |
| AC026355.1 | ZNF84 | 0.552164491 | 5.04E-44 |
| AC026355.1 | ZNF841 | 0.546421395 | 5.66E-43 |
| AL606489.1 | ZNF841 | 0.508647164 | 1.51E-36 |
| AC026355.1 | ZNF852 | 0.682509139 | 1.36E-74 |
| AC026355.1 | ZNF91 | 0.713173315 | 2.81E-84 |
| AC026355.1 | ZNRF2 | 0.512533154 | 3.59E-37 |
| AC026355.1 | ZRANB2 | 0.540726461 | 5.95E-42 |
| AC026355.1 | ZSWIM6 | 0.550112696 | 1.20E-43 |
| AC026355.1 | ZYG11B | 0.609304557 | 1.11E-55 |

Abbreviations: cor, correlation coefficient
